# Immunological maladaptation preceding spontaneous preterm birth in human pregnancies

**DOI:** 10.1038/s41467-026-75605-5

**Published:** 2026-07-27

**Authors:** Ina A. Stelzer, Joshua Gillard, Christopher Urbschat, Kristin Thiele, Dorien Feyaerts, Mirja Pagenkemper, Ann-Christin Tallarek, Bettina Hollwitz, Masaki Sato, Edward Ganio, Maïgane Diop, Kazuo Ando, Jakob Einhaus, Nima Aghaeepour, David K. Stevenson, Nicola Gagliani, Anna Woestemeier, Stefan Bonn, Anke Diemert, Petra C. Arck, Brice Gaudillière

**Affiliations:** 1 Department of Anesthesiology, Perioperative and Pain Medicine, Stanford University, Stanford, CA USA; 2 Department of Pathology, University of California San Diego, La Jolla, CA USA; 3 Department of Medicine, Division of Cardiovascular Medicine, Stanford University, Stanford, CA USA; 4 Department of Obstetrics and Prenatal Medicine, University Medical Center Hamburg-Eppendorf, Hamburg, Germany; 5 Hamburg Center for Translational Immunology, University Medical Center Hamburg-Eppendorf, Hamburg, Germany; 6 Department of Pediatrics, Stanford University, Stanford, CA USA; 7 Department of Biomedical Data Science, Stanford University, Stanford, CA USA; 8 Institute for Inflammation and Carcinogenesis, University Medical Center Hamburg-Eppendorf, Hamburg, Germany; 9 Department for General, Visceral, Thoracic and Vascular Surgery, University Hospital of Bonn, Bonn, Germany; 10 Institute of Medical Systems Bioinformatics, Center for Biomedical AI (bAIome), Center for Molecular Neurobiology Hamburg (ZMNH), University Medical Center Hamburg-Eppendorf, Hamburg, Germany; 11 German Center for Child and Adolescent Health, Hamburg, Germany

**Keywords:** Biomarkers, T-helper 17 cells, Gene expression analysis, Reproductive biology

## Abstract

The majority of spontaneous preterm births (sPTB) occurs without identifiable clinical indications or apparent risk factors. A dysregulated maternal immune adaptation at delivery has been associated with sPTB. Yet, a precise understanding of maternal immune dynamics preceding sPTB remains lacking. Here we show, in a nested case-control study within a low-risk, population-based pregnancy cohort, that an abnormal immune adaptation in mothers’ blood precedes sPTB by weeks to months and discriminates sPTB cases from term controls (AUROC: 0.7). Prominent features include enhanced immune cell responses to an adrenergic stimulus during the first and second trimesters, followed by increased production of pro-inflammatory cytokines in the third trimester in sPTB vs. term pregnancies. Transcriptome analysis of second trimester single-cell CD4^+^ T cells reveals a Th17-skewed, neuroactive-protein responsive phenotype in sPTB pregnancies. Our study provides a multi-omics resource and a conceptual framework for early identification of individuals at increased risk for sPTB with broad translational implications for advancing targeted preventive measures.

## Abstract (web summary)

Spontaneous preterm birth often occurs without any clinical warning sign and in the absence of identifiable risk factors. Here, the authors perform a multi-omics analysis of longitudinally collected peripheral blood samples from low-risk pregnancies and identify distinct immune parameters, such as increased adrenergic responsiveness and Th17-skewed CD4^+^ T cells, that precede idiopathic preterm birth.

## Introduction

Across the globe, around 10% of pregnancies end prematurely^1^, leading to more than 13 million infants being born too soon each year. This number has remained concerningly stable despite progress in prenatal care and advanced screening tools^1,2^. As a result, these infants are more likely to experience serious short-term and long-term health issues. In fact, preterm labor (PTL) and subsequent preterm birth (PTB) remain the leading causes of morbidity and mortality in children under the age of five^3^. Up to seventy percent of PTB cases occur spontaneously (sPTB) without identifiable clinical indications or apparent risk factors before its onset^4^. Consequently, reliable tools capable of predicting sPTB prior to its clinical manifestation, ideally during early pregnancy, are critically needed. Developing these predictive tools is hindered by our incomplete understanding of the pathobiological drivers that precede sPTB.

Maternal immune adaptation, characterized by a tolerogenic immune response both within the uterine microenvironment and systemically, is fundamental to reproductive success^5,6^. Dysregulation of this immune adaptation has been identified as a key component associated with the development of various pregnancy-related complications, including sPTB^7–13^. Earlier studies aiming to better understand underlying mechanisms measured levels of circulating proteins, metabolites, or lipids, or the gene expression of immune cells, often during PTL rather than in asymptomatic states before its onset, or in populations at high-risk for sPTB^14–16^. While some potential markers of sPTB were detected, no studies have as yet captured the trajectories of functional immune responses throughout pregnancy. Thus, the underlying immunological maladaptation that may precede and contribute to sPTB remains unclear.

Our latest research shows that tracking single-cell immune profiles throughout pregnancy is crucial for pinpointing factors that predict gestational age, preeclampsia, and the time to onset of spontaneous labor^17–20^. Further, the impact of particular lifestyle and environmental factors, e.g., climate change^21,22^, maternal psychosocial stress, and stressful life events^23–27^, is increasingly recognized as a risk factor for sPTB. Our prior preclinical research further underscores that elevated maternal stress perception in mice can impede the progression of pregnancy through pathways associated with inflammation^28^.

This study aims to determine whether immunological maladaptation preceding sPTB can be identified early, in low-risk pregnancies. We use maternal blood samples collected from a prospective, population-based study of low-risk pregnancies with close clinical monitoring. We perform longitudinal multi-omic analysis throughout gestation, combining single-cell mass cytometry, high-content aptamer-based proteomics, and single-cell RNA sequencing. This allows us to comprehensively profile adaptations in innate and adaptive immune cells (maternal immunome), circulating serum proteins (maternal proteome), and T cell gene expression (maternal transcriptome) in pregnancies resulting in sPTB or demographically-matched term birth (TB). The main study objective is to model alterations of maternal immune adaptation to pregnancy across all trimesters before the onset of sPTB. Our study also offers a comprehensive multi-omics resource for use in frameworks designed to understand the dynamics of immune responses during pregnancy, ultimately aiding in the design of preventative and therapeutic strategies.

## Results

### Longitudinal multi-omic monitoring preceding sPTB in a low-risk cohort

**Fig. 1:**
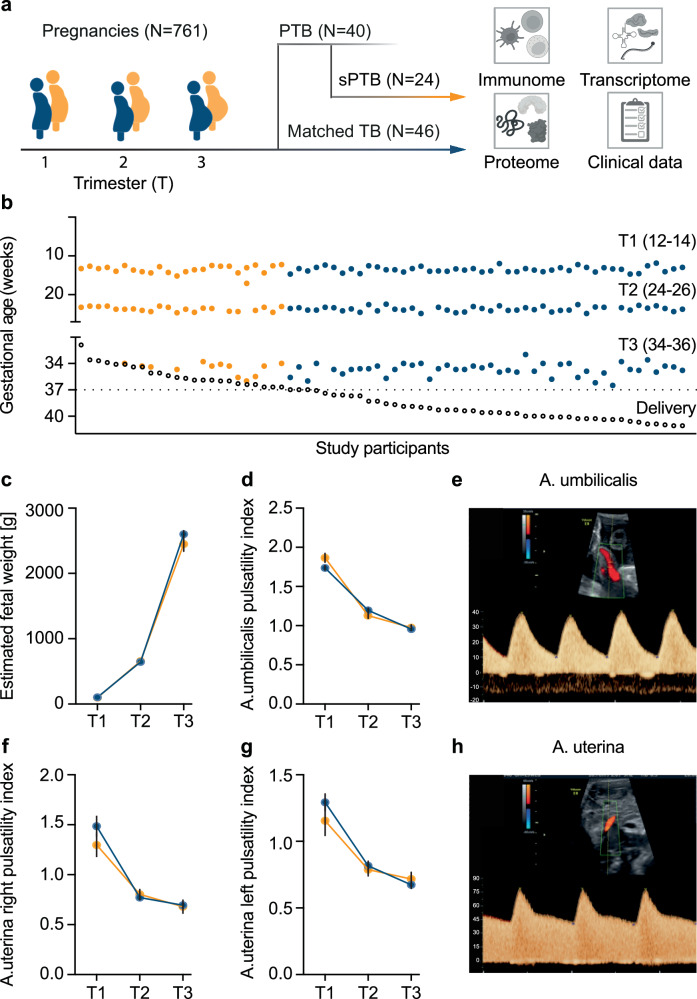
Multi-modal assessment of maternal immunome, proteome, and transcriptome in deeply phenotyped pregnancies. **a** To identify immune maladaptation preceding and predicting sPTB across all trimesters (T1–T3), PBMC were used for the analysis of the systemic immunome (mass cytometry), serum samples were used for the analysis of the circulating proteome (aptamer-based platform). CD3^+^ T cells sorted from PBMC at T2 were used to analyze the single-cell transcriptome (scRNAseq). **b** Pregnant women completed a study visit at one timepoint in each trimester (T, filled circles) at indicated GAs (weeks) (sPTB, *N* = 24, *n* = 60 samples; TB, *N* = 46, *n* = 135). Open circles indicate GA at delivery. See Supplementary Fig. 1 for sample number overviews of PBMC, serum, and single T-cells. **c**–**f** Ultrasound examinations show no differences in fetal growth (**c**), umbilical (**d**), or average placental perfusion (**f**, **g**) across pregnancy. Data shown as Mean ± SEM. **e**, **h** Representative ultrasound images. Cliparts created in BioRender. Stelzer, I. (2026) https://BioRender.com/qgmglce. Source data are provided as a Source Data file.

We conducted a study utilizing peripheral blood samples and accompanying metadata gathered from the prospective, longitudinal, population-based pregnancy study PRINCE (Prenatal Identification of Children’s Health; *N* = 761), hosted at the University Medical Center Hamburg-Eppendorf, Germany. Within this study, a total of *N* = 40 women experienced PTB (5.5%). While this corresponds to the general PTB rate in the city-state of Hamburg (5.9%), it is lower than the national average in Germany (8.3%) and might be explained by Hamburg’s generally high socioeconomic status relative to other German states. Cases with clinical conditions associated with iatrogenic PTB due to, e.g., worsening maternal health due to severe preeclampsia, vaginal bleeding, or confirmed vaginal or ascending uterine infections were excluded. Notably, relevant and significant risk factors for PTB, such as twins, chronic maternal infections, or severe maternal disease, were exclusion criteria in the PRINCE study design and thus redundant for sample selection in the present study. This resulted in the construction of a cohort of women with sPTB (*N* = 24) pregnancies (median gestational age/GA at birth of 35.7 weeks; Fig. 1a, b and Table 1). This cohort was matched for maternal age, first-trimester body mass index, gravidity/parity, and fetal sex to a reference group of *N* = 46 participants with TB (median GA at birth: 39.6 weeks). Study visits including biospecimen collection occurred serially once each trimester in a narrow GA window before delivery (Fig. 1b), resulting in similar GA at sampling across sPTB and TB groups. Ultrasound examinations throughout pregnancy showed no differences between groups in estimated fetal weight, fetal umbilical and placental blood flow, or fetal growth metrics (Fig. 1c–h, Supplementary Fig. 1).

**Table 1:** Cohort overview

DEMOGRAPHICS	PRINCE (N = 761)	TB (N = 46)	sPTB (N = 24)	
	N (Percentage) or Median [Interquartile Range]			p-value (TB vs. sPTB)
**Maternal characteristics**				
Maternal age at delivery (years)	32,74 [30, 35]	31 [29, 33]	32 [30, 34]	0.5965
BMI 1^st^ trimester (kg/m^2^)	23.5 [21.6, 25.8	23.1 [21.3, 24.5]	23.6 [21.3, 27.1]	0.4221
Nulliparous	338 (44.4%)	20 (43.5%)	12 (50%)	0.6093
**Pregnancy details**				
GA at delivery (weeks)	39,9 [38.9, 40.4]	39.6 [38.5, 40.3]	35.7 [34.2, 36.2]	<0.0001
Vaginal birth	563 (75,8%)	34 (73.1%)	12 (50%)	0.0462
Infant sex	372 female (49.5%)	31 female (67.4%)	12 female (50%)	0.1605
Birthweight (g)	3505 [3150, 3820]	3360 [3086, 3740]	2547 [2348, 2801]	<0.0001
5-min Apgar score	10 [10, 10]	10 [9.5, 10]	9.5 [8.0, 10]	0.0280
History of preterm birth	18 (4.3%)	4 (15.4%)	2 (16.7%)	0.9224
**Pregnancy complications**				
Gestational diabetes	34 (4.5%)	3 (6.5%)	0 (0%)	0.2065
Hyper-/Hypothyroidism	77 (10.1%)	3 (6.5%)	1 (4.2%)	0.6922
Pre-existing hypertension	6 (0.8%)	1 (2.2%)	0 (0%)	0.4741

TB and sPTB groups were compared using a *t*-test. Two-sided p-values are provided.

Serial peripheral blood mononuclear cells (PBMC) and serum samples were collected to evaluate the single-cell immunome by mass cytometry and the serum proteome using an aptamer-based platform. PBMC samples were analyzed at baseline (unstimulated) as well as in response to stimulation with an array of inflammatory (mimicking infectious and pro-inflammatory stimuli) and adrenergic (mimicking a stressful stimulus) ligands, providing a comprehensive assessment of maternal immune cell phenotype and function. In a cross-sectional subcohort of women with sPTB (*N* = 10) or TB (*N* = 10), second-trimester CD3^+^ T cells were analyzed by single-cell RNA sequencing (scRNAseq), providing an orthogonal transcriptomic dataset complementing the immunome and proteome data.

### Integrative modeling reveals gestational timepoint-dependent immunopathobiology preceding sPTB

**Fig. 2:**
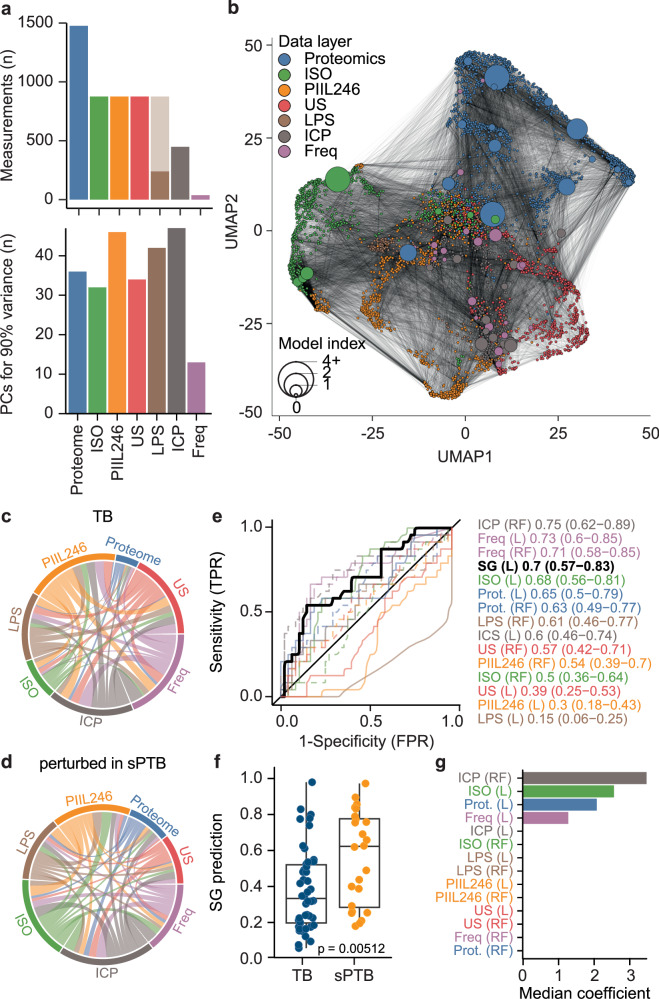
Integrative, time-dependent maternal immunome and proteome modeling differentiates sPTB from TB pregnancies. **a** Integration of the maternal immunome and proteome from T1–T3 to model immunopathology preceding sPTB generated 4826 features per sample. Bar plot showing the number of features split into data layers (omics). Light shaded LPSCL097 responses were penalized during data preprocessing. Degree of internal correlation between features in each omic, represented by the number of principal components (PC). **b** UMAP of the correlation matrix of the feature space. Each node represents a biological feature. Black edges represent correlations that are significantly different in sPTB (FDR < 0.05) compared to TB. Dot size indicates the feature importance (by model index) for predicting sPTB, SG model. **c** Chord diagrams of significant inter-omic (between data layers) correlations in TB and **d** correlations that are significantly different from TB in sPTB. The size of the links is proportional to the total number of significant correlations normalized by the total number of possible correlations. **e** ROC curve and AUC of each single-omic model and the stacked multi-omic SG model (bold black). **f** Prediction scores of the SG model distinguishing sPTB (*N* = 24) from TB (*N* = 46). p: two-sided Wilcoxon rank sum test of prediction scores. Data presented as box plots, with bounds from 25 to 75th percentile, median line, and whiskers extending to the largest or smallest value no further than 1.5*interquartile range. **g** Bar graph showing the weights of each single-omic submodel in the stacked model. Submodel weights were calculated as the median coefficient across cross-validation folds. Source data are provided as a Source Data file.

Major innate and adaptive immune cell phenotypes, their frequencies (Freq) and intracellular functional responses at baseline (unstimulated, US) and following receptor-specific stimulation were analyzed in cryopreserved PBMC using high-parameter single-cell mass cytometry assays (Fig. 2a, Supplementary Figs. 2, 3 and Supplementary Table 1, 2). Stimulants were chosen to mimic sPTB-associated environments, including (i) bacteria-derived TLR-4 agonist lipopolysaccharide (LPS) plus viral RNA mimicking TLR-7/8 agonist CL097 (LPSCL097), (ii) a pro-inflammatory cytokine milieu of Interleukins (IL)2/4/6 plus calcium-releasing phorbol 12-myristate-13-acetate/ionomycin (PIIL246), and, (iii) stress-response associated β-adrenergic agonist isoproterenol (ISO). To test cell function, intracellular cytokine production (ICP) was measured at baseline and following PI stimulation. A total of 4826 single-cell immune features were extracted from each sample and timepoint. In parallel, the proteomic immune cell environment was assessed using an aptamer-based approach, yielding 1476 serum proteomic features per sample and timepoint (Fig. 2a). We assessed intra-omic modularity by calculating the number of principal components (PCs) necessary to explain 90% of the variance within each omic. This analysis revealed many PCs reflecting independent axes of intercorrelated immune and protein analytes and indicated rich information content across the individual immunome (US, LPSCL097, PIIL246, ISO, ICP, Freq) and proteome data layers (Fig. 2a). To visualize immunological re-wiring of the maternal immunome and proteome in sPTB, we calculated correlations between all features at all timepoints in TB pregnancies and visualized this matrix of feature correlations in a two-dimensional network map (Fig. 2b). Correlation coefficients significantly different between sPTB and TB pregnancies (FDR < 0.05) were highlighted (Fig. 2b). Importantly, quantification of the magnitude of change in these correlation coefficients revealed that the absolute difference between sPTB and TB correlation coefficients surpassed 0.5, suggesting marked disruptions in the data correlation structure in sPTB (Supplementary Fig. 4a). Inter-omic correlations that differed between sPTB and TB predominantly concentrated among features related to proteome and ISO data layers (Fig. 2c, d).

This analysis of correlations between data layers (the interactome) over the full course of gestation revealed significant disruptions in immunome–proteome interactions in sPTB compared to TB but did not account for the timing of measurements, emphasizing the need for an approach sensitive to GA variability in maternal immune responses and proteomic environment. To address this, we employed a time-dependent, integrative modeling strategy that incorporated features from all sampling time points (T1 + T2 + T3) and omics data layers for each participant to classify sPTB and TB samples (Supplementary Fig. 4b). This multivariable approach accommodated the high dimensionality of the data while also leveraging the paired samples and longitudinal design of the cohort in the absence of data leakage between modeling steps. We assessed the predictive performance of submodels trained on individual omic datasets using least absolute shrinkage and selection operator (LASSO) and random forest (RF). These submodels were combined into an ensemble model through stacked generalization (SG), which integrates complementary predictive signals across omics layers (Supplementary Fig. 4b). The resulting SG model classified sPTB and TB samples (area under the receiver operator curve [AUROC] = 0.7, 95% confidence interval (CI) [0.57 to 0.83], Wilcoxon rank sum test *p*-value = 0.00512, sensitivity/specificity Supplementary Table 3) (Fig. 2e, f). While the model built from cytokine production (ICP) alone reached a nominally higher AUROC (0.75) compared to the multivariate SG (LASSO) (0.7), these differences were not statistically significant (*p* = 0.36). In fact, no single-omic model significantly outperformed the multi-omic model (all *p* > 0.72) (Supplementary Table 3). The SG (LASSO) model demonstrated the highest specificity (84.8%) among all tested models, making it the most robust choice for minimizing false positives, while capturing cross-omic interactions and providing a systems-level view of sPTB. We also assessed how well the predicted risk corresponded to the observed outcomes for the top four models using calibration curves, revealing that ICP (RF) was indeed well-calibrated, whereas the SG (LASSO) model displayed a slight tendency towards overconfidence (Supplementary Fig. 4c). A confounder analysis ascertained that the accuracy of the SG model was not correlated with various clinical or demographic variables, including maternal age, BMI, gravidity/parity, history of prior PTB, and fetal sex (Supplementary Table 4). Lastly, we performed a post-hoc power analysis using the method of Obuchowski et al. as implemented in the pROC R package^29,30^. With 24 cases and 46 controls, our study had 80% power (α = 0.05, two-sided) to detect an AUC of 0.697 as significantly different from chance (AUC = 0.5). Our observed leave-one-out cross-validated AUC of 0.7 corresponds to a power of 81.2%, exceeding the conventional 80% threshold. Further, as an alternative approach to the classification of sPTB vs. TB based on the clinical cut-off of 37 weeks’ gestation, we predicted the continuous variable GA at delivery from the same data layers with modest, yet significant regression performance between true and predicted age at delivery (Spearman *r* = 0.358, *p*-value = 0.002, Root Mean Standard Error/RMSE = 2.1 weeks) (Supplementary Fig. 4d). We concluded that, for the prediction of risk for sPTB, grouping clinical outcomes based on the clinical cut-off shows superior performance to predicting gestational age at delivery.

Finally, we evaluated the performance of this model in an external, publicly available data set of whole blood samples serially collected from sPTB and TB pregnancies^19^, reaching an AUC of 0.92 in the integrated model (Supplementary Fig. 4e–g, Supplementary Table 5,6). While the results require additional validation in a larger patient cohort, the magnitude and consistency in effect direction observed in the model scores on the test set provided supportive evidence for the external validity of our multi-omic model.

We continued to examine the unbiased set of features that informed the classification of sPTB vs. TB (Fig. 2e, f). The most informative data layers for predicting the SG model were the omic data layer of cytokine production (ICP), followed by the adrenergic response to ISO, the serum proteome, and immune cell subset frequencies (Freq) (Fig. 2g).

### Immunological maladaptation before the onset of sPTB involves enhanced immune cell responses to an adrenergic stimulus during the first and second trimester

**Fig. 3:**
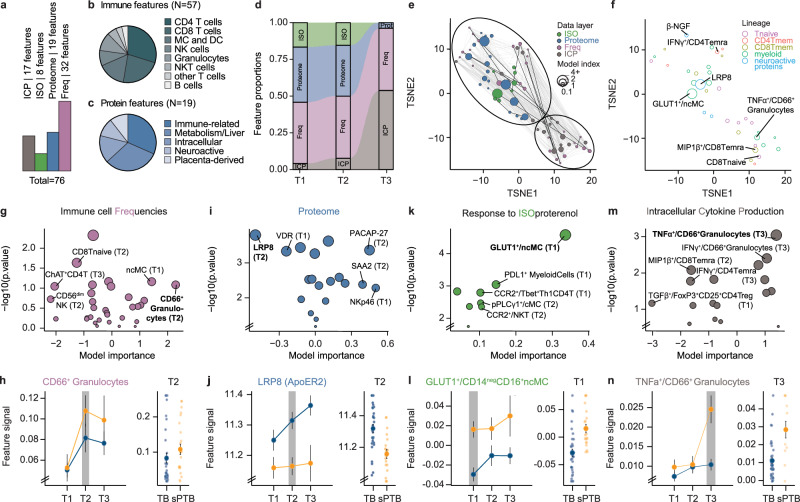
Time-dependent immunological maladaptation preceding sPTB. **a** 76 model features informed the classification into sPTB and TB. **b** The 57 immune features predominantly consist of CD4 (17 features), CD8 (13 features), and monocyte/DC features (nine features), while **c** the majority of proteome features (12 out of 19) are categorized into immune- and metabolism/liver-related proteins. **d** Proportion of all model features across trimesters. **e** t-SNE plot of the top informative features (nodes). Gray edges indicate correlations between features in TB, while correlations between features significantly different in sPTB (FDR < 0.05) are shown by black edges. Dot size reflects the overall model importance (index). Circles denote manually assigned clusters. **f** Immune cell lineage and protein mask overlaying the feature network in (**e**), highlighting selected immune cell subsets, and neuroactive proteins enriched via pathway enrichment analysis. **g**, **i**, **k**, **m** Volcano plots of features selected by submodels contributing to the stacked model, with the top five features annotated and overall feature importance for the stacked model displayed on the x-axis (model index). Y-axis represents the univariable, two-sided Wilcoxon p-value of a feature distinguishing sPTB from TB. **h**, **j**, **l**, **n** Trajectories of the top feature from each data layer/submodel contributing to the stacked model (left). The predictive timepoint selected (shaded in gray) by the submodel is illustrated as a scatter plot with individual patients’ values (right). **h**, **j** T2 PTB *N* = 23, TB *N* = 41; **l** T1 PTB *N* = 24, TB *N* = 42; **n** T3 PTB *N* = 13, TB *N* = 43; Data presented as the mean ± SE of winsorised (5th, 95th percentile) values. ncMC: CD14^neg^CD16^pos^ non-classical monocyte, cMC: CD14^pos^CD16^neg^ classical monocyte, Treg: regulatory, Temra_:_ effector memory re-expressing CD45RA T cell. Source data are provided as a Source Data file.

The time-dependent analysis of maternal immunome and proteome yielded a precise classification of sPTB vs. TB cases before the onset of labor, highlighting sPTB-related responses within distinct immunological and proteomic data layers. To facilitate the biological interpretation of the multivariable SG model, we extracted the features that contributed most to the model’s overall performance. These informative features included 17 ICP, eight ISO, 19 proteomic, and 32 immune cell frequency (Freq) features (Fig. 3a, Supplementary Data 1, Supplementary Figs. 5–8). The predominant characteristics observed within the immune cell population were associated with CD4 T cells, encompassing memory, naïve, and various Th subsets, which accounted for 17 features. This was followed by 13 memory, naïve, and effector CD8 T cell features. Additionally, monocytes and dendritic cells were collectively represented with nine features, while low-density granulocytes contributed six features (Fig. 3b). Among the 19 proteomic features, six features were categorized as immune-system/tumor necrosis factor (TNF)-related, six as metabolism/liver-related, and others as neuroactive, intracellular or placenta-derived (Fig. 3c, Supplementary Fig 7). Calculating the relative contribution of informative features to the prediction over time (i.e., trimesters), the adrenergic cell response to ISO and serum proteomics features informed the model performance in the first and second trimester, long before the onset of clinical symptoms. In contrast, pro-inflammatory cytokine profiles (ICP) primarily informed the model performance in the third trimester (Fig. 3d).

To understand the relationships between the set of informative features, a correlation network was calculated that segregated the features into two groups (Fig. 3e): in the group on the lower right, cytokine production (ICP) features clustered with frequency features of naïve T cells, while in the group on the upper left, ISO response and proteome features grouped myeloid and T cell responses with neuroactive proteins (tissue transglutaminase, low-density lipoprotein receptor-related protein 8 (LRP8), fibronectin, neuronal growth factor β (β–NGF) (Fig. 3e, f). Additional analyses revealed that these neuroactive proteins drove the enrichment of pathways related to nervous system function, including the regulation of nervous system development and the regulation of neurogenesis (Supplementary Table 7, Supplementary Fig. 9a).

Examination of the most informative model features within the proteomic and immunome data layers, derived from our unbiased approach, confirmed previously described biology and revealed new insights into the pathobiology preceding PTB (Fig. 3g-n).

Within the immunome data layers of Freq, ICP, and ISO (Supplementary Figs. 5, 6, 8), we found profound and cell-type-specific differences in innate and adaptive immune cell responses between sPTB and TB samples (Fig. 3g, k, m). In innate immune compartments, low-density granulocytes were more abundant from T2 onwards (Fig. 3g, h), coinciding with a relative decrease in CD4 and CD8 T cell proportions (Supplementary Fig. 10). While low-density granulocytes within the PBMC fraction do not necessarily linearly reflect the proportion of total granulocytes, this finding is consistent with prior studies suggesting PTB is associated with a high neutrophil-to-lymphocyte ratio (NLR) as determined using whole blood in clinical settings^31^. In parallel, low-density granulocytes from sPTB samples exhibited increased basal IL-12 production and heightened TNFα and IFNγ cytokine production in response to PI stimulation at T3 compared to the near absence of cytokine production in TB samples, aligning with previous observations of their pro-inflammatory state at the time of sPTB^32^ (Fig. 3m, n and Supplementary Fig. 10).

Remarkably, immune cell responses to adrenergic stimulation (ISO), including the expression of glucose transporter 1 (GLUT1) in non-classical monocytes, were elevated in sPTB compared to TB samples, as early as in T1 (Fig. 3k, l). Non-classical monocytes were also comparatively more abundant in sPTB samples (Supplementary Fig. 10). Other responses to adrenergic stimulation included the upregulation of PD-L1 and phosphorylation of PLCγ1 in myeloid cells (Fig. 3k and Supplementary Fig. 6).

Within adaptive immune cell compartments (Fig. 3b, k, m), Tbet^+^ Th1 CD4 T cells from sPTB samples exhibited increased CCR2 expression in response to ISO, indicating enhanced chemotactic capacity upon adrenergic stimulation (Supplementary Figs. 6, 10). In the Th2 compartment, frequencies of CRTH2^+^ Th2 CD4 T cells were reduced in sPTB compared to TB (Supplementary Figs. 8, 10). Hinting towards differences in Th17 function between TB vs. sPTB cases, IL-17A production was diminished in naïve CD4 T cells in sPTB (Supplementary Figs. 5, 10). Importantly, FoxP3^+^CD25^+^ regulatory CD4 T (Treg) cells released lower amounts of TGFβ in response to PI stimulation (Fig. 3m and Supplementary Fig. 10). This finding, together with the variable levels of IL-2 produced by CD4 and CD8 T cells throughout sPTB gestation (Supplementary Fig. 10), is consistent with previously reported instability of feto-maternal immune tolerance, which includes changes in Treg cell phenotype and function associated with PTL^33,34^.

To externally validate the findings within the single-immunome submodels, we identified a study among publicly available data sets of sPTB prediction that measured the bulk whole-blood transcriptome in the second and third trimester explicitly in sPTB vs. sTB (“Dream” study)^20^. This study analyzed blood at timepoints that overlapped with our study (Supplementary Fig. 9b). Using blood transcription modules, we identified patterns that reflected signatures we report in the top-predictive Freq and ICP single-omic models (Fig. 3g, m and Supplementary Fig. 9c). Specifically, granulocyte, T-cell activation, and monocyte inflammation modules confirmed our cytometric findings of increased monocyte, granulocyte, and CD8 effector frequencies and/or cytokine production.

When examining the proteome contributing to the classification of sPTB vs. TB (Supplementary Fig. 7), we found previously unrecognized metabolic and neuroendocrine protein features associated with sPTB, rather than an enrichment of the expected inflammatory proteins. The most informative protein was Low-Density Lipoprotein Receptor-Related Protein (LRP8), also known as Apolipoprotein E Receptor 2 (ApoER2), which was reduced in serum throughout gestation in sPTB compared to TB samples (Fig. 3i, j). LRP8 is a protein expressed in the placenta and associated with fetal growth restriction^35,36^, and one of several metabolic and liver-related predictive proteomic features, including serum amyloid A (SAA2), vitamin D receptor (VDR), and alanine aminotransferase (ALT). In addition to Nerve Growth Factor-β (β–NGF), neuroactive proteins included pituitary adenylate cyclase-activating peptide/PACAP-27 (Supplementary Fig. 7, Supplementary Data 1). These proteins collectively reveal previously underappreciated interactions between metabolism, neuroendocrine state, and pregnancy progression, with yet to be explored links to immune cell states.

Overall, our findings extend the current understanding of the pathobiology preceding sPTB. We provide compelling evidence that an enhanced adrenergic immune response during early pregnancy represents a fundamental element of the immunopathobiology underlying sPTB. Our data also emphasize an augmented pro-inflammatory innate immune response and functional shift of CD4^+^ T cells, including regulatory T cells, reinforcing the notion that a maladaptation of the maternal immune response early in pregnancy may contribute to the subsequent precipitation of sPTB.

### Maternal blood T cells exhibit a transcriptional program weeks before the onset of sPTB, characterized by enhanced sensitivity to adrenergic and neuroactive signals

Our mass cytometry analysis emphasized the role of T cells as crucial mediators of maternal immune maladaptation in sPTB. To further investigate this cell population, we conducted an orthogonal analysis of the T cell transcriptome using scRNASeq. This analysis was designed as an exploratory investigation to identify candidate biological pathways, not as a definitive gene-level discovery effort. Gene expression profiles of single T cells collected in the second trimester were generated (Supplementary Fig. 11a–c). We selected the second trimester, compared to the first and third trimesters, as it represents a window of relative stability of maternal-fetal immune adaptations, and is a clinically accessible period with fewer confounding factors that is suitable for timely intervention. In this context, we evaluated a subcohort of sPTB and TB from within our cohort (*N* = 10 TB, *N* = 10 sPTB), where women with two consecutive pregnancies were analyzed, allowing to control for inter- and intra-individual variability (Table 2 and Supplementary Fig. 11a–d). Conducting differential gene expression analysis, we found that the number of differentially expressed genes (DEG) was higher when comparing sPTB vs. TB than when comparing the transcriptome signature between a prior vs. a consecutive pregnancy. Thus, gene transcriptional profiles are driven more strongly by sPTB status than by birth order, with intra-patient (within-woman) variability across pregnancies being modest relative to inter-patient differences associated with sPTB. Accordingly, inclusion of two pregnancies from the same woman in a unified cohort was justified to maximize power for detecting sPTB-associated signatures (Supplementary Fig. 11d).

**Table 2:** Cohort overview for scRNA-seq analysis

DEMOGRAPHICS	1^st^ pregnancy		2^nd^ pregnancy	
	TB (N = 5)	sPTB (N = 5)	TB (N = 5)	sPTB (N = 5)
	N (Percentage) or Median [Interquartile Range]			
**Maternal characteristics**				
Maternal age at delivery (years)	29.0 [26.5, 32.0]	31.0 [28.5, 34.5]	32.0 [30.0, 36.0]	33.0 [31.0, 36.5]
BMI 1^st^ trimester (kg/m^2^)	22.40 [21.60, 24.85]	21.40 [21.10, 22.85]	21.40 [21.05, 23.45]	22.30 [21.85, 27.00]
Nulliparous	1 (20.0%)	1 (20.0%)	0 (0.0%)	0 (0.0%)
**Pregnancy details**				
GA at delivery (weeks)	39.30 [37.50, 40.50]	34.10 [33.65, 34.90]	39.00 [38.75, 40.40]	35.10 [34.00, 36.70]
Vaginal birth	4 (80.0%)	1 (20.0%)	3 (60.0%)	5 (100.0%)
Infant sex	2 female (40.0%)	2 female (40.0%)	3 female (60.0%)	2 female (40.0%)
Birthweight (g)	3360 [3003, 3638]	2535 [2000, 2714]	3270 [3118, 3575]	2670 [2093, 3010]
5-min Apgar score	10 [9.25, 10]	9 [8.5, 10]	10 [8.5, 10]	10 [10, 10]
History of preterm birth	0 (0.0%)	0 (0.0%)	3 (60.0%)	2 (40.0%)
**Pregnancy complications**				
Gestational diabetes	0 (0.0%)	0 (0.0%)	0 (0.0%)	0 (0.0%)
Hyper-/Hypothyroidism	0 (0.0%)	0 (0.0%)	0 (0.0%)	0 (0.0%)
Pre-existing hypertension	0 (0.0%)	0 (0.0%)	0 (0.0%)	0 (0.0%)

**Fig. 4:**
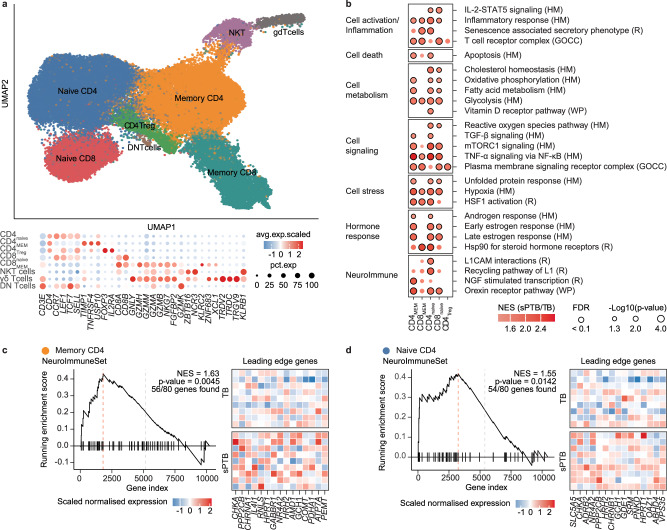
Second trimester T cells are transcriptionally distinct prior to sPTB vs. TB. **a** Single-cell RNA sequencing of T cells at 24 weeks GA to deep-phenotype the adaptive response prior to premature delivery. UMAP visualization of all single cells (*N* = 33,538) across 18 total samples. UMAP of RNA expression profiles of marker genes (top). Cell populations are labeled within the plot (bottom). **b** Gene set enrichment analysis (GSEA) was used to calculate the normalized enrichment score (NES) of MSigDB gene sets (Gene Ontology: Cellular Component/GOCC, Reactome/R, Wikipathways/WP, KEGG, and Hallmark/HM) for each subpopulation using a list of all genes ranked by the log2-fold difference between sPTB and TB samples. Statistical significance and p-values were calculated against an empirical null distribution and reflect two-sided tests. Selected gene sets are shown (nominal p-value < 0.05); and those with FDR < 0.1 highlighted with a black border. Gene sets are grouped based on their biological function on the right margin. **c**, **d** GSEA plots of the neuroimmune gene set for (**c**) memory and (**d**) naïve CD4 T cells, including the top 15 leading-edge genes. Statistical significance and p-values were calculated as above. Nominal p-values are shown and no adjustment for multiple comparisons was performed. Source data are provided as a Source Data file.

T cells clustered into naïve and memory CD4 and CD8 as well as Treg cells, in line with previously described transcriptional profiles^37,38^ (Fig. 4a). Comparing gene expression between sPTB and TB cases for CD4 naïve, CD4 memory, CD8 naïve, CD8 memory, and Tregs identified DEGs, mostly in memory and naïve CD4 T cells (Supplementary Fig. 11d–f). Gene set enrichment analyses (GSEA) revealed an increased expression of genes in pathways involved in activation/inflammation, cell metabolism, signaling, stress, hormone responses, and neuroimmune responses in sPTB compared to TB T cells (Fig. 4b, Supplementary Data 2). This signature was most pronounced in CD4 naïve and memory T cells. Notably, gene sets indicating translation and ribosomal activity were the only pathways decreased in sPTB CD4 naïve and memory T cells. Among the upregulated pathways related to stress hormone responses was the pathway ‘HSP90 steroid hormone receptors’—chaperones facilitating glucocorticoid entry into the nucleus^39^—in naïve CD4 T cells. At the same time, inflammatory and TNFα signaling (consistent with increased TNF pathway-related proteins among serum proteins (Supplementary Fig. 7)) were enhanced across CD4 and CD8 naïve and memory subsets (Fig. 4b and Supplementary Fig. 11). These observations support the hypothesis of a steroid-responsive (i.e., stress-responsive), pro-inflammatory environment preceding sPTB in the second trimester. Dysregulations in sPTB hormone responsiveness are further illustrated by enhanced androgen and estrogen responses (Fig. 4b, Supplementary Fig. 12). In accordance with increased levels of VDR in sPTB serum (Supplementary Fig. 7), naïve CD4 T cells exhibited enhanced expression of the VDR signaling pathway, alongside other metabolic pathways suggesting a sPTB-specific metabolic profile (Fig. 4b). A neuroimmune gene expression cluster, comprised of β--NGF and orexin signaling pathways, was elevated in memory CD4 T cells (Fig. 4b and Supplementary Fig. 12). In line with this observation, serum levels of β--NGF, one of the informative protein features of the model, were increased across sPTB pregnancies (Supplementary Fig. 7 and Supplementary Data 1). This suggests that T cells can engage in signaling induced by neuroactive proteins.

To investigate the interactions among genes that are differentially regulated in sPTB versus TB, a gene network analysis was conducted with a particular emphasis on the transcriptionally reprogrammed population of CD4 memory T cells (Supplementary Fig. 13). Both inter- and intra-pathway correlations of DEG in sPTB were significantly increased compared to TB and over a randomly permutated correlation matrix. This suggests that the alterations in gene expression associated with sPTB occur in a highly coordinated fashion across transcriptional programs and pathways.

To further refine phenotypic clustering within memory CD4 T cells into T helper subgroups (Fig. 4a, b), we conducted an enrichment analysis of curated gene sets sourced from the existing literature^40^ (Supplementary Data 2). Th17 and Th2 gene expression signatures were increased, while Th1 signatures were decreased in sPTB compared to TB (Supplementary Fig. 14). Representative examples of this finding of enhanced systemic Th17 profiles preceding sPTB were a notable upregulation of the chemokine *CCL20*, which is associated with Th17 cell activity, along with an increase in *RORC*, the transcription factor critical for Th17 cell differentiation, when enriching for a carefully selected interleukin gene set in memory CD4 T cells (Supplementary Fig. 14c)^40^.

Having characterized transcriptional reprogramming in T cell subsets in sPTB, we aimed to deepen our findings of enhanced adrenergic immune responsiveness, which significantly contributes to the longitudinal classification of sPTB from TB. We curated a specialized set of genes involved in signaling, biosynthesis, transport, and metabolism of neurotransmitters (Supplementary Data 3) and examined their enrichment and differential expression in CD4 naïve, CD4 memory, CD8 naïve, CD8 memory, and Tregs derived from sPTB cases. Notably, naïve and memory CD4 T cells in sPTB exhibited a marked increase in the expression of the neuroimmune gene set compared to TB, while no other subsets showed differential expression (Fig. 4c, d). A representative example among these genes was protein phosphatase 2 catalytic subunit beta (*PPP2CB*), a key component of the adrenergic signaling cascade, upregulated in sPTB memory CD4 T cells (Fig. 4c). Expression of adrenoceptor beta 2 (*ADRB2*), the target of the adrenergic agonist ISO utilized for stimulation for functional immunoprofiling in this study, was found to be upregulated in naïve CD4 T cells (Fig. 4d). Increased receptor expression may underlie the increased adrenergic responsiveness observed on protein level (Fig. 3k, l and Supplementary Fig. 6, 10). We externally validated these single-cell transcriptomic findings, testing for enrichment of our custom neuroimmune gene set (used in the scRNAseq analysis in Fig. 4d, e), at each timepoint in the “Dream” study, a whole-blood bulk transcriptomic data set of sPTB vs. TB cases^20^. Demonstrating convergent pathway-level results (Figs. 3c, 4c, d), neuroimmune-related genes were positively enriched (increased expression, trending towards significance) in the second trimester (GA weeks 16-22 and 22-26) in sPTB vs. TB cases. Congruent with our finding of a lack of ISO feature contribution to sPTB risk prediction in late pregnancy, this association was lost in the third trimester (GA weeks 26-37) (Supplementary Fig. 15). Collectively, along with the enrichment of β--NGF, orexin, and other signaling cascades related to neuroactive proteins (Fig. 4b), these findings suggest the presence of a dysregulated peripheral neuroimmune synapse in the immunopathobiology of sPTB. Overall, we report pathway-level associations of second-trimester single-T cell transcriptomic profiles with sPTB, providing a foundation for hypothesis generation to test causal links in future studies.

### The serum proteomic profile during early pregnancy is predictive of sPTB

**Fig. 5:**
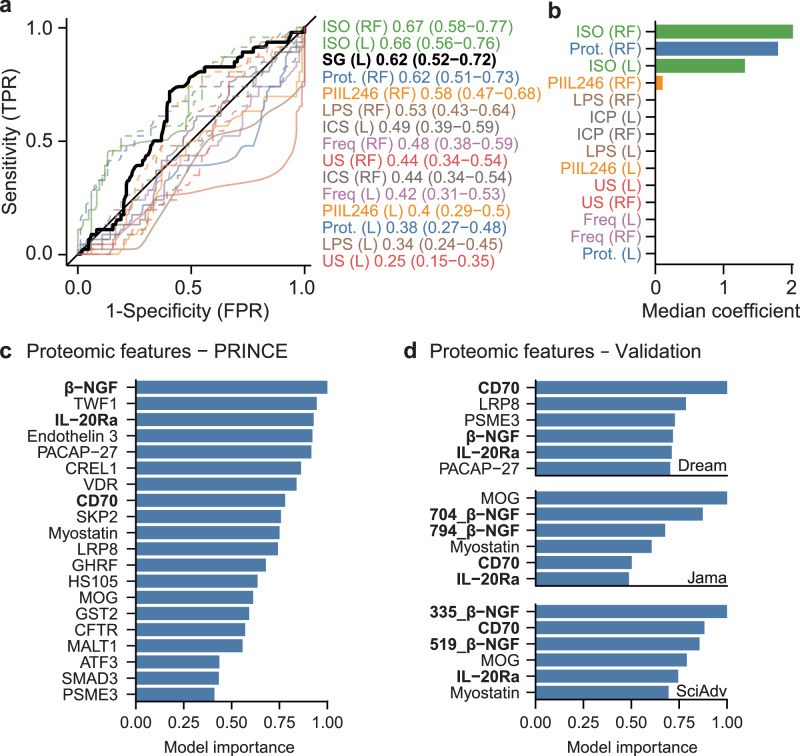
Proteomic markers identified in early pregnancy serve as predictive indicators of sPTB. **a** ROC curve and AUC of the sample-level multi-omic model distinguishing sPTB from TB using data from T1 and T2, displaying each single-omic model and the stacked model (bold black). **b** Bar graph showing the weights of each single-omic submodel in the stacked model. Submodel weights were calculated as the median coefficient across all cross-validation folds. **c** Model features of the proteomic data layer ranked by feature importance (index). Features identified here were extracted from publicly available proteomic sPTB datasets to validate the proteomic sPTB prediction model. **d** Proteomic model features identified in and validated to be predictive of sPTB in the Jama, SciAdv, and Dream cohorts. Source data are provided as a Source Data file.

The longitudinal analysis of immune cell and serum proteomic features that distinguish individuals with sPTB from TB highlighted trimester-dependent immune responses that may contribute to the immunopathogenesis of sPTB. From a clinical perspective, the early identification of patients at risk for sPTB during pregnancy is imperative, ultimately facilitating the implementation of personalized and effective preventive strategies. To identify early predictive markers of sPTB in asymptomatic individuals, immune cell and serum proteomic features measured during the first and second trimesters of pregnancy served as input for a multivariable predictive model of sPTB (Supplementary Fig. 4b). An SG model integrating all omic data layers successfully predicted sPTB (AUC = 0.62, CI [0.52 to 0.72], Fig. 5a and Supplementary Fig. 16). Interestingly, models developed based only on adrenergic cell responses (ISO) or serum proteomic data layers demonstrated noteworthy predictive capabilities for sPTB, surpassing or meeting similar performance of the SG model (ISO: AUC = 0.67, CI [0.58 to 0.77]; Proteome: AUC = 0.62, CI [0.51 to 0.73]); Fig. 5a, b and Supplementary Data 4). A confounder analysis indicated that the predictive accuracy of the SG model was not significantly affected by clinical or demographic variables (Supplementary Data 5).

To externally validate the proteome-based prediction, three publicly available plasma proteomic studies of sPTB cases provided independent datasets. Of the 19 serum proteomic model features identified in the proteome submodel (Fig. 5c, Supplementary Data 4, and Supplementary Fig. 16), six features were measured in the “Dream” study, while five features overlapped with those measured in both the “Jama” and “SciAdv” validation studies (Fig. 5d). Utilizing these subsets of quantified features, novel models were built and predicted sPTB with AUCs of 0.6 (CI 0.52 to 0.67), 0.67 (CI 0.55 to 0.79), and 0.61 (CI 0.54 to 0.68), in “Dream”, “Jama”, and “SciAdv”, respectively. Notably, β–NGF, IL-20Ra, and CD70 demonstrated validation in all three studies, establishing themselves as biomarker candidates amidst demographic and gestational-age variability across study cohorts (see Methods for cohort demographics). In brief, the validation cohorts differed significantly in geographical range (six sites in Asia, Africa, and North America) and race (Asian, African, African American) from the test cohort, which is Middle European and Caucasian. These discrepancies might explain the lower model performance in these data sets. Despite these differences, β–NGF, IL-20Ra, and CD70 surfaced as predictive across all data sets, suggesting generalizability across demographic domains, which will require additional validation in future studies. Collectively, our findings illuminate a set of potential early markers for sPTB, characterized by neuroactive and proinflammatory functions (Supplementary Figs. 5, 16).

## Discussion

Despite numerous investigations into the immunopathobiology of sPTB^16,41^, an integrated and precise description of the functional immune changes associated with sPTB pregnancies is still incomplete. Previously, transcriptional profiles of samples obtained during term parturition revealed perturbations detectable at the clinical onset of sPTB^42–44^. Other studies profiling second-trimester blood bulk transcriptome or first-trimester PBMC-derived microRNA provided important glimpses into differential gene expression associated with sPTB^20,45–48^. These studies suggested that immune maladaptation of impending PTL may be detectable before the onset of symptoms.

We designed the present study to examine the phenotypic and functional immune adaptation longitudinally, before the onset of sPTB. Multivariable modeling of time-dependent immune cell and serum proteomic dynamics revealed trimester-specific components in individuals with sPTB vs. TB. We characterized a distinct neuroimmunological signature associated with sPTB and detectable as early as the first trimester of pregnancy. We identified a pro-inflammatory profile in the third trimester, when the pathogenesis of sPTB may begin to manifest. Key elements of this neuroimmune and inflammatory signature of sPTB, described on the proteomic level, were validated using an external data set and orthogonally explored based on a transcriptomic approach. Moreover, predictive modeling identified early biomarker candidates of sPTB, including β–NGF, CD70, and IL-20Ra, which were independently validated in publicly available datasets.

While multiple etiologies, including infection/inflammation, cervical insufficiency, and vaginal microbial dysbiosis, are associated with sPTB, a substantial body of epidemiological evidence also identifies maternal psychosocial stress and stressful life events as important contributing factors^23–27^. Dysregulation of the hypothalamic-pituitary-adrenal axis has been proposed as a potential mechanistic link between sPTB and increased stress perception, resulting in elevated levels of stress hormones that significantly impact the immune response. However, current literature on the relationship between stress perception and immune system dynamics remains inconsistent, underscoring the necessity for further investigation into this interaction^49–51^. Our findings highlight that the quality of the immunobiological response to a stressful stimulus during early pregnancy, specifically an adrenergic response to a catecholaminergic agonist, is associated with adverse birth outcomes later during gestation. This is in line with a recent analysis of mid-gestation urine metabolites in over 1000 pregnant participants, which found a strong association between sPTB and elevated urine levels of catecholamines^52^. Heightened activity of the sympathetic nervous system may serve as a biological indicator of an autonomic imbalance, possibly engaging the immune system through its direct interaction with catecholamines. This study presents evidence supporting this proposed mechanism for the first time.

Some limitations of our study warrant consideration. First, while the cohort of sPTB cases is characterized in great detail regarding clinical and immunological features, participant recruitment was geographically and demographically constrained to a single site in North-Western Europe. This resulted in a West European cohort with very low population diversity. Yet, our study is unparalleled in its analysis depth and number of patients. Further studies are now essential to ascertain the generalizability of neuroimmunoendocrine mechanisms implicated in the pathobiology of sPTB in other populations. Although our cohort is relatively small, as is common for discovery-stage modeling studies, we performed post-hoc power analyses to assess whether the study had sufficient sensitivity to detect discriminatory performance above chance (AUC = 0.5). These analyses indicated that the observed AUCs were detectable under the sample size of our study. However, large international, multi-center studies with sizable, well-characterized patient groups are required to establish model stability, reduce overfitting, and establish reproducibility and reliability of the identified signals. Our study can serve as the foundation for the construction of such cohorts to ultimately take relevant steps towards improved clinical management. Second, although the frequency of blood sampling at one time point per trimester is unparalleled in the existing literature, our investigation does not fully elucidate the dynamic changes that occur throughout pregnancy. Consequently, the precise temporal points at which shifts in immune composition occur as well as tipping points towards the neuroimmune and inflammatory signature observed before the onset of sPTB remain elusive. For example, our investigation is limited by the absence of preconception assessments, which would enable us to determine whether the immune signature observed in the first trimester preexisted or developed concurrently with the initiation of gestation, an essential aspect for designing intervention programs. Third, the lack of assessments of cervicovaginal profiles or placental histopathology hinders an interpretation of our systemic findings as a potential reflection of local profiles in reproductive organs and at the maternal-placental interface. Studying local tissues such as the placenta or decidua may reveal sPTB-specific phenotypes and pathways, and offer unique mechanistic insights into pathobiology, given that uterine and cervicovaginal immune cell composition and function differ from blood and include specialized populations, such as decidual NK cells. These are neglected in analyses of peripheral blood. Yet, obtaining uterine tissue during pregnancy without a medical indication is not feasible, while analyses of placental and decidual tissues at delivery capture pathology only after sPTB has occurred.

Furthermore, it is essential to note that the interactions among various features remain mere associations until substantiated by mechanistic evidence. Although our study presents a comprehensive new set of longitudinal relationships between different immunological features, these remain unexplained at the mechanistic level. Our analysis reveals that GLUT1 expression in non-classical monocytes during the first trimester is the strongest adrenergic response, distinguishing sPTB from TB. Typically, GLUT1 is a marker for immune activation and is necessary to sustain a proinflammatory state in monocytes^53^. Furthermore, high expression of GLUT1 associates with the modulation of migratory capacities of non-classical monocytes through the upregulation of CCR2, such that in proinflammatory states, non-classical monocytes can be found in tertiary lymphoid organs, where they utilize their full immunoregulatory potential via PD-L1^53–55^. Further, PD-L1 expressed by non-classical monocytes is a potent regulator of T cell function, as it enhances T cell apoptosis and dampens immune tolerance induced by Tregs^55,56^. This is reflected in our finding that low levels of Treg-derived TGFβ, a key cytokine for maintaining and expanding the immune regulatory capacity of Tregs^57,58^, is one of the strongest features in the ICP data layer distinguishing sPTB and TB in the first trimester. To fully understand the potential cell communication network between non-classical monocytes and regulatory T cells, and to close the knowledge gap we have left open, further functional studies need to be conducted, for example leveraging GLUT1 or PD-L1 inhibitors in in vitro co-culture systems.

Lastly, from a translational perspective, we included analyses aimed at identifying potential blood biomarkers indicative of risk for sPTB as early as possible during pregnancy. Obtaining blood is a low-risk procedure that can be readily incorporated into routine prenatal care. The predictive value, including sensitivity/specificity of cytokine production capacity, cell frequency, protein composition, or adrenergic response, will ultimately become apparent in clinical applications, i.e., for a sPTB risk test. Clinical labs can readily determine serum protein levels or Complete Blood Counts, reporting immune cell frequencies. Cytokine production and adrenergic response assays, while currently not universally available in clinical labs, are attainable to establish in dedicated facilities.

In conclusion, our investigation elucidates several pathways associated with sPTB, offering a valuable resource for formulating hypotheses and informing future mechanistic studies. Our research underscores the significance of generating comprehensive phenotypic and functional cell atlases over a defined temporal framework to enhance our understanding of sPTB pathobiology. To further advance our comprehension of immune adaptation to pregnancy and its dysregulation in the context of sPTB, it will be essential that future research cohorts employ a multi-center, multi-faceted approach. Lastly, careful attention must be given to social determinants of health to ensure the protection of pregnant individuals through robust scientific inquiry^59^.

## Methods

### PRINCE pregnancy cohort

The ongoing prospective pregnancy cohort PRINCE (Prenatal Identification of Children’s Health) was initiated in 2011 at the University Medical Center Hamburg-Eppendorf. Women of legal age with a singleton pregnancy were recruited in the 1st trimester (gestational age/GA, 12–14 weeks) and subsequently invited to two subsequent prenatal visits in the 2nd (GA, 24–26 weeks) and 3^rd^ trimester (GA, 34–36 weeks). The presence of a multiple pregnancy, maternal chronic infections (HIV, hepatitis B/C), known substance abuse, severe maternal disease, and pregnancies conceived after assisted reproductive technologies precluded study participation. Smoking and alcohol use, risk factors for PTB, were also designated as exclusion criteria in the PRINCE study. Still, we inquired about alcohol intake and smoking habits during the first study visit. In total, 10.4% of all participants (*N* = 741) reported smoking during the early weeks of pregnancy, with similar numbers between women with subsequent TB or sPTB, but they quit once pregnancy was confirmed. None of the study participants reported alcohol consumption after pregnancy confirmation. Therefore, we did not include smoking and alcohol as confounding variables in our modeling approach. Following childbirth, information on pregnancy outcome was provided by clinical records in combination with self-reports. This information included GA at delivery, delivery mode, child’s sex, and birth weight, and the occurrence of pregnancy complications, e.g., PTB. In total, *N* = 40 pregnancies with PTB were identified among the *N* = 761 participants of the PRINCE study (PTB rate: 5.5%). Among these total PTB cases, an onset of labor resulting in delivery occurred in 24 women, whereby onset of labor was defined as contractions and preterm premature rupture of membranes (PPROM). These cases were classified as spontaneous PTB (60% sPTB of all PTB cases) and used for the analysis presented in our current study. Women who experienced iatrogenic PTB (e.g., induced delivery before gestation week 37 due to preeclampsia, fetal distress or growth restriction, vaginal bleeding, or uterine infections were excluded from the present analyses (N = 16). Notably, relevant and significant risk factors for PTB, such as twins, chronic maternal infections, or severe maternal disease, were exclusion criteria in the PRINCE study design. It is well known that risk factors for PTB can be multifaceted and include, e.g., obesity, lower income, high stress levels, and short pregnancy spacing. We controlled for these risks by a clear set of matching criteria when selecting the reference cases from the group of women with TB pregnancies. Matching criteria were maternal age, first-trimester maternal body mass index, gravidity/parity, and fetal sex. TB controls were chosen from the full cohort in a 2:1 TB:sPTB ratio.

The PTB rate of 5.5% in our study is lower than the national averages in Germany (8.3%), the European Union (7.2%), and the US (10.4%)^60,61^. This lower rate may be attributed to high-quality, standardized perinatal care and universal health insurance, as well as a high socioeconomic status, specifically in Hamburg. This socioeconomic advantage is linked to better nutrition, higher physical activity, earlier prenatal visits, reduced smoking during pregnancy, and fewer untreated infections, likely contributing to the low PTB rate observed in the PRINCE study.

### Inclusion & ethics

All PRINCE study participants signed an informed consent form. The study protocol was approved by the ethics committee of the Hamburg Chamber of Physicians (license number PV 3694) and was conducted according to the Declaration of Helsinki for Medical Research involving Human Subjects.

### Monitoring of clinical parameters

During all three antenatal visits, study participants were clinically examined to document pregnancy progression and maternal health/anthropometric data. Detailed information on nutrition and medication intake was obtained, and a maternal venous blood sample was taken. In addition, a detailed materno-fetal ultrasound was performed by two fully trained fetal medicine specialists with certified advanced ultrasound expertise and in accordance with the guidelines of the International Society of Ultrasound in Obstetrics and Prenatal Medicine (ISUOG) using a Voluson E8 (General Electric; GE), equipped with a transabdominal 3–5 MHz transducer (RAB 6D, GE). The examination included fetal parameters such as head and abdominal circumference and femur length to estimate fetal weight (1st trimester according to Warsof, 2nd and 3rd trimester according to Hadlock IV^62^ and perfusion of the A. umbilicalis. To calculate the average pulsatility index (PI) of the A. uterinae, the mean of at least three consecutive consistent flow waveforms was recorded by pulsed Doppler after visualization using color flow on both sides. The measurements were performed in a sagittal plane of the uterus with an ultrasound transducer placed in either the left or right iliac fossa of the abdomen, directed towards the lateral uterine walls and downwards into the pelvis.

### Blood sample processing

#### Isolation of peripheral blood mononuclear cells (PBMC)

During each study visit (T1: median, 13 weeks GA; T2: median, 24 weeks GA; T3: median, 34 weeks GA), pregnant participants donated blood by venous puncture into an EDTA-coated tube. All samples were processed within 2 hours after collection. Peripheral blood mononuclear cells (PBMC) were isolated from whole blood using Biocoll (Biochrome/Merck) gradient centrifugation. PBMC were stored until further processing in RPMI1640 medium supplemented with 20% heat-inactivated FBS and 10% DMSO in liquid nitrogen.

#### Serum isolation

Blood was collected into polystyrene tubes and allowed to coagulate for 45 min. The sample was then centrifuged (1500 g, 20 min) at 4 °C within 60 min. Separated and aliquoted serum was stored at –80 °C until further processing.

#### In vitro stimulation of PBMC

Liquid-nitrogen preserved PBMC were quickly thawed in a 37 °C waterbath and washed twice (300xg) in pre-warmed complete (c)RPMI media (RPMI1640 + 10% FBS, 1% Penicillin/Streptomycin, 1% L-Glutamine). Cells were then resuspended in 5 ml cRPMI and rested for 1 hr with loose caps in an incubator (37 °C, 5% CO_2_). Following rest, cells were spun and resuspended, then split for either an intracellular cytokine assay or an intracellular signaling protein phosphorylation assay.

For the intracellular cytokine assay, 0.5–1 × 10^6^ cells per sample were incubated with Golgi Plug (Brefeldin A, BD) and Golgi Stop (Monensin, BD) in the presence or absence (unstimulated control) of phorbol 12-myristate 13-acetate (PMA)/ionomycin (0.5X, eBioscience) for 2 hours with loose caps in an incubator (37 °C, 5% CO_2_).

For the intracellular signaling protein phosphorylation assay, 0.5–1 × 10^6^ cells per sample were incubated in 4 conditions: (1) unstimulated (vehicle cRPMI), (2) ultra-pure LPS (1 μg/ml, Invivogen) + CL097 (1 μg/ml, Invivogen), (3) PMA/ionomycin (0.5X, eBioscience) + human recombinant Interleukin-2, Interleukin-4, Interleukin-6 (each 100 ng/ml, R&D), (4) isoproterenol hydrochloride (100 μM, Tocris Bioscience). Concentrations were chosen based on experimental testing^63^. All samples were incubated for a total of 30 min in a 37 °C waterbath. Stimulants of condition (2) and (3) were added after 15 min of preincubation and incubated for 15 min. Stimulant of condition (4) was added at the start of the stimulation, for a total incubation time of 30 min.

Following both intracellular cytokine assay and intracellular signaling protein phosphorylation assay, Proteomic Stabilizer (SmartTube Inc.) was added to each sample, and incubated at room temperature for 10 min for cell fixation. Cells were transferred to cryotubes and stored at –80 °C until further processing.

### Mass cytometry of PBMC samples (Immunome)

#### Antibody panels

Two mass cytometry antibody panels were used to phenotype immune cell subsets and to detect either intracellular signaling protein phosphorylation (Supplementary Table 1) or intracellular cytokine production (Supplementary Table 2). Antibodies were either obtained preconjugated (Standard Biotools Inc.) or were purchased as purified, carrier-free (no BSA, gelatin) versions, which were then conjugated in-house with trivalent metal isotopes utilizing the MaxPAR antibody conjugation kit (Standard Biotools Inc.). All antibodies used in the analysis were titrated and validated on samples that were processed identically to the samples used in this study.

#### Barcoding and antibody staining

To achieve single-sample barcoding for sample pooling, cells were transiently permeabilized with saponin and barcoded with a combination of Palladium metal isotopes (102 ^Pd^, 104-106 ^Pd^, 108 ^Pd^, 110 ^Pd^), following a 3-out-of-6 scheme, allowing to pool 20 samples^64^. Then, after incubation with anti-human Fc block (Biolegend), pooled barcoded cells were stained with surface antibodies for 30 min at room temperature with gentle agitation (600 rpm). For intracellular signaling protein phosphorylation staining, cells were then permeabilized with methanol and stained with intracellular antibodies, again for 30 min at room temperature with gentle agitation (600 rpm). For intracellular cytokine staining, cells were permeabilized with saponin and stained with intracellular antibodies, for 1 h at room temperature with gentle agitation (600 rpm). Pools of 20 barcoded and antibody-stained samples were analyzed on the mass cytometer instrument (Helios CyTOF, Standard Biotools Inc.), acquiring two sets of pooled samples (40 samples total) per day.

#### Minimization of experimental batch effect

To minimize the effect of experimental variability on mass cytometry measurements between serially collected samples, samples corresponding to the entire time series collected from one participant and its matched participant from the other group were processed, barcoded, pooled, and stained simultaneously.

To minimize the effect of variability between study participants, sample sets of two participants were run per day and the run was completed within consecutive days, while carefully controlling for consistent tuning parameters of the mass cytometry instrument.

To control for variability between runs of barcoded and pooled samples, an internal control sample, stimulated similarly as the actual samples, was distributed across all sample pools and measured simultaneously, indicating signal variation occurring across time and sample pools.

To control for signal amplification shifts during the runs, normalization beads were added to each pooled sample. The mass cytometry data from all pooled samples was normalized using Normalizer v0.1 MATLAB Compiler Runtime (MathWorks)^65^.

#### Processing of mass cytometry data and generation of a single-cell feature matrix of the immunome

Files were de-barcoded with a single-cell MATLAB debarcoding tool^64^. Manual, supervised gating was performed using CellEngine (CellCarta, Montreal, Canada)^66^, clustering immune cell populations according to conventional surface marker composition. Cell populations as shown in the gating strategy (Supplementary Fig. 3) were included in the analysis (37 immune cell subsets).

Population frequencies among all mononuclear cells (among all living cells for the granulocyte population), their endogenous intracellular activities of signaling proteins (e.g., median signal intensity of phosphorylated protein) or their frequencies of cytokine-positive cells per population, and the capacities of each cell subset to respond to stimulation (e.g., delta between unstimulated and stimulated condition (median expression or frequency)) were exported. Additionally, median signal intensities of metabolic, chemotactic, epigenetic, and activation markers were exported, resulting in a multi-class feature matrix with 4185 single-cell immune features per sample.

We penalized the data matrix and excluded features based on prior biological knowledge (e.g., signaling responses in TLR4-deficient T cells upon LPS stimulation) to reduce noise^67^. In total, 835 features (none in ICP, 50 in ISO, 50 in PIIL246, 50 in US, 685 in LPSCL097) were penalized, resulting in a final matrix of 3350 immunome features per sample (Supplementary Data 6).

From the data matrix, the following data layers were constructed: Freq: frequencies of 37 immune cell subsets; US: unstimulated/endogenous (intra)cellular activities such as the phosphorylation states of 9 signaling proteins, as well as 13 metabolic, epigenetic, chemotactic, and functional markers; Evaluation of functional capacities of each immune cell subset: ICP: ability to produce intracellular cytokines when stimulated with phorbol 12-myristate-13-acetate/ionomycin (PI), including the unstimulated(US)/endogenous levels of cytokines; LPSCL097: ability to respond to bacterial and viral exposure with TLR4-agonist lipopolysaccharide (LPS) and synthetic TLR7/8-agonist CL097 (a derivative of the imidazoquinoline compound R848); PIIL246: ability to respond to a proinflammatory cytokine environment with a combination of Interleukins (IL)−2, −4, and −6, in conjunction with PI; ISO: ability to respond to a neurotransmitter of the sympathetic nervous system, via β-adrenergic agonist and norepinephrine mimetic isoproterenol.

### Serum analysis (Proteome)

The serum samples (120 μL per sample) were analyzed using a highly multiplexed, aptamer-based platform capturing 1500 proteins at the company’s site (SomaLogic, Inc., Boulder, CO)^68,69^. The assay quantifies proteins over a wide dynamic range ( > 8 log) using chemically modified aptamers with slow off-rate kinetics (SOMAmer reagents). Each SOMAmer reagent is a unique, high-affinity, single-strand DNA endowed with functional groups mimicking amino acid side chains. In brief, samples were incubated on 96-well plates with a mixture of SOMAmer reagents. Two sequential bead-based immobilization and washing steps were used to eliminate nonspecifically bound proteins, unbound proteins, and unbound SOMAmer reagents from protein target-bound reagents. After eluting SOMAmer reagents from the target proteins, the fluorescently labeled reagents were quantified on an Agilent hybridization array (Agilent Technologies Inc.). Data were normalized in four specific steps and according to assay data quality control procedures defined in the good laboratory practice quality system of SomaLogic, Inc. Normalization steps controlled for signal intensity biases introduced by differential hybridization efficiencies and the overall brightness of plates, collection protocol artifacts, and batch effects between different plates.

### Bioinformatic methods for immunome and proteome

#### Multiomic interactome analyses

For each feature, we regressed the feature signal from all timepoints on the patientID label with a linear model and extracted the residuals. Thereafter, pairwise Spearman correlations were calculated across all timepoints with the residuals of each feature in each group. This approach ensures that the resulting correlations reflect feature covariations over time within individuals by removing patient-specific baseline effects. Benjamini-Hochberg (BH) correction^70^ was applied (FDR = 0.05) to the resulting sets of correlations (TB and sPTB) to control for multiple hypothesis testing. This was visualized for TB pregnancies as gray lines in Fig. 2b. Significantly different correlations (FDR = 0.05) were calculated between TB and sPTB pregnancies with the R package ‘cocor’^71^ and visualized as black lines in Fig. 2B. To visualize interomic correlations, pairwise correlations were classified as intra- or inter-omic based on dataset membership of the corresponding feature pairs. Thereafter, correlations were filtered (FDR < 0.05) and visualized using a chord diagram (R software, circlize package^72^). The number of significant interomic correlations was normalized to the total number of possible correlations between any two datasets.

### Prediction of spontaneous preterm birth with longitudinal multiomic data

#### Data configurations for machine learning analyses

To predict PTB with multiomic longitudinal data, we evaluated two data configurations with machine learning. Data configurations refer to the unit of observation for model training and evaluation (Supplementary Fig. 4b). In the patient-level data configuration, the unit of observation is the patient. This configuration of the data addresses whether the immune trajectory of each patient can predict PTB or gestational age at birth. Data from all three timepoints (T1 + T2 + T3) were used to predict PTB. In the sample-level data configuration, each individual blood sample is the unit of observation. This configuration of the data addresses whether sets of immune variables can predict PTB at multiple timepoints during gestation. Data from the first two timepoints (T1 + T2) were used to predict PTB.

#### Leave-one-patient-out cross-validation and data preprocessing

We employed a nested leave-one-patient-out cross-validation (LOOCV) approach in all modeling to prevent overfitting and to obtain an unbiased estimate of model performance on unseen samples. The overall cross-validation design comprised three nested levels: (i) an outer LOOCV loop used to evaluate the stacked generalization ensemble; (ii) an inner LOOCV loop within the outer training set used to train and generate out-of-sample predictions from each submodel; and (iii) a grouped V-fold cross-validation within each inner training iteration used for hyperparameter tuning. Within each training set, data preprocessing was applied per feature in the following order: imputation of missing values using the median; winsorization at a 0.05 quantile threshold; exclusion of zero-variance features; centering by subtracting the mean; and scaling by dividing by the standard deviation. Preprocessing parameters estimated from the training set were then applied to the corresponding test set. For classification analyses, a weighted loss function was used during model training to address class imbalance.

#### Multivariable analyses and stacked generalization

Model training followed the nested LOOCV structure described above. For each omic dataset, submodels were trained within the inner LOOCV loop using the least absolute shrinkage and selection operator (Lasso^73^) and Random Forest (RF^74^) using the Tidymodels^75^ machine learning framework. For classification analyses, Lasso was implemented as penalized logistic regression with L1 regularization; for regression analyses (predicting gestational age at birth), penalized linear regression with L1 regularization was used. The penalty parameter λ was tuned via 5-times repeated, 20-fold grouped cross-validation, where folds were constrained such that all samples from a given patient remained within the same fold to prevent data leakage. Folds were stratified by the response variable in classification analyses. A grid of 100 candidate penalty values was evaluated, selecting the value that optimized the area under the receiver operating characteristic curve (AUROC) for classification or root mean squared error (RMSE) for regression. Random Forest classifiers were trained with 1,000 trees using the ranger engine with impurity-based variable importance. The top 25 features ranked by importance were used to train the final RF submodel, following the approach established in the DREAM PTB challenge^20^.

To integrate predictive signals across omics layers, we used stacked generalization (SG^76^) in the outer LOOCV loop. In each outer iteration, submodels were trained on the training data using the inner LOOCV loop. Their out-of-sample predictions were then used as inputs for a second-layer Lasso model constrained to non-negative coefficients, yielding a weighted combination of submodels. The SG layer processed submodel predictions with mean imputation. This nested cross-validation design ensured that SG performance estimates were unbiased and avoided information leakage between submodels and the ensemble layer.

#### Snakemake pipeline

We designed a Snakemake pipeline to facilitate training and evaluating the individual models across configurations. The pipeline comprised three sequential rules: (1) training of submodels across all omic layers in parallel; (2) training of the stacked generalization ensemble using submodel predictions; and (3) model evaluation and analysis. To enable parallel execution, all outer cross-validation splits were predefined as omic layer–patient pairs, allowing independent model training iterations to be distributed across available cores.

#### Model evaluation and feature importance

For classification analyses, model performance was assessed using the area under the receiver operating characteristic curve (AUC) with 95% confidence intervals, computed on pooled out-of-fold predictions. Model performance was assessed using a Wilcox test comparing prediction scores of each group (TB or sPTB), and model accuracy was calculated as the area under the Receiver Operating Characteristic (ROC) curve (AUC). Prediction scores for each patient or sample were calculated as the mean score across cross-validation folds. A Wilcoxon rank-sum test was used to compare prediction scores between TB and sPTB groups. For regression analyses, model performance was assessed using Spearman’s rank correlation between predicted and observed gestational age at birth, along with root mean squared error (RMSE). Prediction scores for each patient or sample were calculated as the median prediction score across cross-validation folds.

For Lasso and Random Forest models, variable importance was calculated as the median importance score across cross-validation folds. The importance score for a variable of the Lasso model was defined as the absolute value of the model coefficient. The importance score of a variable of the random forest model was determined with the decrease of node impurity. To quantify the contribution of individual features through the stacked model, we extracted features from submodels with positive median SG coefficients. For each submodel, importance scores were normalized by the maximum importance score within that submodel, yielding a relative importance score. The SG model contribution index for a feature was calculated as (-log10(p-value) * relative importance score), where the p.value was derived from a Wilcoxon rank-sum test (classification) or Spearman’s correlation test (regression) for that feature.

#### Model comparison and calibration

To compare predictive performance across models, we computed the area under the receiver operating characteristic curve (AUROC) with 95% confidence intervals, along with sensitivity and specificity at the optimal classification threshold determined by Youden’s J index. Formal pairwise comparisons between the stacked generalization model and individual submodels were performed using a two-sided DeLong test for correlated ROC curves. Model calibration was assessed using reliability diagrams, in which predicted probabilities were grouped into five quantile-based bins and plotted against observed event rates, with a LOESS calibration curve fitted to the unbinned predictions.

#### Confounder analyses

A post-hoc linear regression analysis was used as a statistical method to account for clinical or demographic variables that may impact the predictive accuracy of each model. We considered the following variables: “Age”, “BMI”, “Gravidity”, “Parity”, “History_priorPTB”, and “Infant_Sex”. This analysis regresses prediction scores of the cross-validated model with the clinical and demographic variables against the group label (TB vs sPTB). This approach helps to determine whether the predictive model’s outputs are significantly associated with the outcome when other variables are considered. We report the coefficient and associated p.value for each covariate, and results of each model are shown in Supplementary Data 1, Supplementary Table 4, Supplementary Data 5.

#### Validation cohorts

Previous studies of TB and PTB pregnancy with comparable multiomic approaches were used for independent validation of hypotheses inferred from observations made in this study. Particularly, these studies used similar multiomic modalities and had publicly available data.

##### Jama cohort^77^

The plasma samples in the Jama study were derived from five biorepository-supported cohorts in Matlab, Bangladesh; Lusaka, Zambia; Sylhet, Bangladesh; Karachi, Pakistan; and Pemba, Tanzania. A total of 81 pregnant women were included, of which 39 had sPTBs (48.1%) and 42 had TB pregnancies (51.9%). Medically-indicated PTB deliveries were excluded. The mean (SD) maternal age was 24.8 (5.3) years. The median GA at sampling was 13.6 weeks.

##### SciAdvances cohort^14^

Plasma samples in the SciAdv study were derived from five biorepository-supported cohorts in Matlab, Bangladesh; Lusaka, Zambia; Sylhet, Bangladesh; Karachi, Pakistan; and Pemba, Tanzania. This cohort was entirely separate from the Jama cohort^77^. 113 pregnant women with a PTB (48.9%) and 118 with a TB delivery (51.1%) were included, of which N = 224 (97.8%) occurred spontaneously. Median (IQR) maternal age was 25 (22–29). Samples were collected before gestational week 20.

##### Dream cohort^20^

Samples in the Dream cohort were derived from the Perinatology Research Branch, Wayne State University, and the Detroit Medical Center in the US. A bulk transcriptomic data set of *n* = 65 controls and *n* = 34 sPTB samples was available with 2–5 samples per participant. Mean maternal age (IQR) was 25 (21–28), 23.5 (21–27), and African American race 93.8%, 88.2%, respectively. Blood samples were collected at the time of prenatal visits, scheduled at four-week intervals from the first or early second trimester until delivery, during the following gestational-age intervals: 8–16 weeks, 16–24 weeks, 24–28 weeks, 28–32 weeks, 32–37 weeks, and >37 weeks.

In the same study, a total of *n* = 210 plasma samples (two samples per participant) were available from *N* = 39 controls, *N* = 62 sPTB, and *N* = 4 PPROM cases. Mean maternal age (IQR) was 25 (22–27), 24 (21–28), and 24 (21.8–26.2), and African American race 87.2%, 95.2%, and 75%, respectively. Blood samples were collected at the time of prenatal visits, scheduled at 17–22, and at 27–33 weeks gestation.

##### SciTranslMed cohort^19^

Samples in the STM study were collected from *N* = 63 pregnancies with spontaneous onset of labor at term (*N* = 58) or preterm (*N* = 5) at a single North-American site. Median (IQR) maternal age in the training cohort (*N* = 53) was 33 (30, 35) and White, East Asian, African American race 35.9%, 30.2%, 0%, respectively. Blood samples were collected in the last 100 days before delivery; time to labor at sampling was −36 ( − 71, −15).

#### Validation of proteomic signatures

In total, twenty protein markers were identified in the random forest sample-level analysis of early pregnancy (timepoints T1 + T2) of the PRINCE cohort. The predictivity of these proteomic features was validated using publicly available data of three studies, described above^14,20,77^. Using the data of each validation cohort, these protein markers were used to newly train and evaluate a random forest model with leave-one-patient-out cross-validation. Not all proteomic features were measured in all validation cohorts. Specifically, 6/20 markers were measured in Dream; 6/20 in SciAdvances; 6/20 in Jama, and relative importance these markers for the prediction of sPTB is shown in the respective figure of each validation cohort.

#### Validation of multi-omic features

To assess the generalizability of the multi-omic model, we leveraged an independently generated longitudinal multi-omic cohort from a previous study that collected integrated mass cytometry and aptamer-based proteomic data during pregnancy, described above (“SciTranslMed” study)^19^. Gestational age at sample collection was aligned between the two studies by binning into matching time intervals, restricting the analysis to T2 and T3 timepoints. When multiple samples from the same patient fell within a single time interval, the earliest sample was retained. Only patients with samples at both T2 and T3 were included, yielding a test set of six sPTB samples (*N* = 3 patients) and eight TB samples (*N* = 4 patients). The analysis was restricted to overlapping features, i.e., the same immune cell frequency features and proteins measured by both the training and test cohort platforms (matched by UniProt identifiers) (Supplementary Table 5, 6).

To account for batch effects between cohorts, features in the SciTranslMed dataset were standardized (z-scored) within the cohort rather than applying the training cohort’s preprocessing parameters. This approach removes cohort-level location and scale effects while preserving within-cohort relative differences, enabling evaluation of whether the learned model weights maintain discriminative ability in an independent dataset. For the frequency and proteomic submodels, model scores were computed as the linear combination of standardized feature values weighted by the corresponding Lasso coefficients from the training study. Submodel scores were then standardized and combined using the stacked generalization weights to produce a final ensemble score. Model discrimination in the cohort was evaluated using the area under the ROC curve (AUROC) with 95% confidence intervals, and group differences were assessed with the Wilcoxon rank-sum test.

### Single-cell transcriptome analyses

#### Cohort description

Within our selective cohort, we identified 10 women who participated in the PRINCE study with two consecutive pregnancies. Of these participants, two women experienced two consecutive TB and two women experienced two sPTB. The remaining six women had one TB and one sPTB. Of these six, three women first had a TB and a subsequent sPTB, while the other three women first had a sPTB and then a TB pregnancy (Supplementary Fig. 11b).

#### Sorting of maternal T cells

Frozen PBMCs were thawed in a 37 °C warm water bath until cells were completely thawed. Cells were washed using pre-warmed (37 °C) RPMI1640 medium supplemented with 5% of heat-inactivated FBS and centrifuged at 450 g for 5 min at room temperature. Subsequently, cells were counted using a Neubauer counting chamber. PBMCs were blocked with ChromPure Human IgG to prevent non-specific binding of antibodies. Antibodies against the extracellular antigens CD45 and CD3 in pre-determined dilutions were added and incubated for 30 min at 4 °C. Afterwards cells were washed with PBS first followed by two washing steps with annexin V binding buffer. Supernatant was discarded and cell pellet was incubated for 20 min at room temperature in a dark place with annexin V-PE and eFluor 506 viability dye (eBioscience) to avoid sorting of early and late apoptotic and dead cells. After the final centrifugation step cells were resuspended in PBS containing 2 mM EDTA to prevent cells from clotting during the process of cell sorting. CD45^+^ CD3^+^ eFluor 506^neg^ Annexin-V^neg^ T cells were sorted using a BD Aria Fusion cell sorter (BD Biosciences) and used for single cell sequencing.

#### Single-cell sequencing of pre-sorted maternal T cells

Pre-sorted CD3^+^ T cells were loaded on a 10x Genomics Chromium instrument to generate single-cell gel beads in emulsion (GEMs). Single-cell RNA-Seq libraries were prepared as described by the 10x Genomics Single Cell 5′ Reagent Kit user guide and using the following Reagent Kits: Chromium Next GEM Single Cell 5’ Library & Gel Bead Kit v1.1 (PN-1000165), Human T Cell (PN-1000005), Chromium Next GEM Chip G (PN-1000120), Single Index Kit T Set A (PN-1000213).

#### Derivation of the single-cell gene matrix

Raw FASTQ files (Read1 contains the 8-nucleotide barcode + 8nt UMI and Read2 contains the mRNA sequence) for each plate were mapped to the human transcriptome (HG38) using the STAR aligner^78^. Uniquely mapped reads were demultiplexed per cell using the known cell barcodes, of which the mapping to specific wells in the 384-well plate is known. Unique RNA molecules were counted and summed per gene using the UMI sequence. Finally, a cell x gene matrix (*N* = 10,655 cells in total) was built using Seurat^79^.

#### Data filtering, normalization, clustering, and cell type annotation

Cells were filtered from the dataset if they were >3 median-absolute deviations for library size, the number of unique genes, or mitochondrial counts, using the quickPerCellQC() function of scater R package^80^. Multivariate outliers were also removed using the same quality-control variables. Two low-quality samples were excluded after filtering due to very low cell counts ( < 500 cells). Gene expression data was normalized using functions of the Seurat v5 data analysis pipeline^81^. In order, these are: NormaliseData(normalization.method = “LogNormalise”); FindVariableFeatures(selection.method = “vst”, nfeature = 6000); ScaleData(vars.to.regress = “MotherID”, “nCount_RNA”, “percent.mt”). Note that data scaling included MotherID, to regress batch effects attributable to each patient. Lastly, RunPCA() function was used to calculate the principal components of the dataset, of which the top 36 (capturing 90% of variance) were retained for downstream analysis. Clustering of cells into populations was achieved with the FindNeighbours() function followed by FindClusters() with resolution set to 0.16. Cell populations were annotated based on positive expression of phenotypic genes curated from the literature^37,38^. Naïve CD4 (*CD4, CCR7, LEF1, TCF7, SELL*); Memory CD4 (*CD4, TIMP1, TNFRSF4, USP10*); Naïve CD8 (*CD8A, CD8B, CCR7, LEF1, TCF7, SELL*); Memory CD8 (*CD8A, CD8B; GNLY, GZMH, GZMM, GZMA, GZMB, NKG7, FGFBP2*); Treg (*FOXP3, IL2RA*); gd T cells (*TRDV2, TRDC, TRGV9, KLRB1*); NK T cells (*ZBTB16, NCR3, KLRC2, ZNF683, XCL1*); DNT cells (*CD3E, CCR7, LEF1, TCF7, SELL*, negative for other markers). Cells were visualized with the RunUMAP() function of Seurat.

#### Pseudobulk differential expression analysis

We created ‘pseudobulk’ RNA libraries by computationally pooling the gene expression of cells in each cell population for each PBMC sample. The total number of cells in each pseudobulk sample for each population is shown in Supplementary Fig. 11. NK T cells, DNT cells, and gd T cells were excluded from downstream analyses since these populations represent by-products of CD3-based cell sorting to isolate T cells and pseudobulk samples of these cell populations comprised few cells ( < 50 cells for most samples). Raw gene counts of cells within a pseudobulk sample were summed together to generate pseudobulk libraries for downstream analyses. We used the DESeq2 bulk RNAseq pipeline to perform differential gene expression analysis based on the developer’s directions^82^. To identify genes associated with PTB, we analyzed gene expression of each population separately with a model that specifies the TB/sPTB birth label as the covariate of interest and controls for subject-level variations. We also analyzed gene expression with a model that specifies the delivery order (prior or consecutive delivery) to identify genes that are associated with repeated/multiple pregnancies. The output from the DESeq2 pipeline includes a gene matrix for each subpopulation with log2 fold change (D1 / D0) and FDR-corrected p-values. These gene matrices were used for downstream analysis of differentially expressed genes and gene set enrichment analyses.

#### Gene set enrichment analyses

Enriched gene sets were identified using gene set enrichment analysis (GSEA^83^) using the pre-ranked list of genes mode. GSEA was calculated using the ClusterProfileR R package^84^ using 10,000 permutations. Differential gene sets of scRNAseq populations were calculated using the log2 fold change expression (sPTB/TB). Genes with fewer than 200 counts in total across all samples were excluded from analysis. Gene sets in the human MSigDB collections were extracted using the MSigDBR R package^85^ and analyzed. This included Hallmark gene sets^86^, curated gene sets comprising Reactome^87^, Wiki Pathways^88^, KEGG gene sets^89^, and Gene Ontology (GO)^90^. We also analyzed T-helper cell gene signatures derived from Seumois et al.^40^ with GSEA. Statistical significance and nominal *p*-values were calculated against an empirical null distribution and reflect two-sided tests. A false discovery rate (FDR) adjusted *p*-value < 0.1 was used to infer significance. In addition to the MSigDB collection analysis, we manually curated a gene sets including genes involved in neurotransmitter signaling, synthesis, transport, and metabolism (Supplementary Data 3). Of the 447 genes, 80 had detectable expression across T cells subpopulations and were defined as the NeuroImmune set (Fig. 4c, d) and analyzed by GSEA (sPTB/TB).

For microarray validation analyses, differential expression was performed using limma, with duplicateCorrelation to account for repeated samples from the same individual. Genes were ranked by log fold change from the limma model for GSEA input. For microarray analyses, blood transcription modules^91^ and the NeuroImmune set above were analyzed by GSEA.

To assess whether proteomic features identified by the multiomic model were enriched for specific biological pathways, we performed over-representation analysis using MSigDB Gene Ontology Biological Process gene sets. Statistical significance and p-values were calculated using a one-sided upper-tail hypergeometric test as implemented in the fgsea R package^92^, testing whether proteins from each pathway were over-represented among the model-informative proteins relative to the measured proteomic background. Shown are pathways with nominal *p*-value < 0.05 and overlap > 3.

#### CD4^+^ memory gene network analyses

Leading edge genes of enriched gene sets of CD4^+^ memory T cells were extracted to construct a correlation network. These gene sets were grouped according to biological function to calculate inter- and intra-group correlations. The following representative gene sets were selected and corresponding groups were analyzed: “Th17 signature genes” Seumois et al.^40^ as a single group; Wiki Pathways “Orexin Receptor Pathway” as the Neuro-Immune group; Hallmark gene sets “Androgen response” and “Estrogen response (early & late)” in the Hormone response group; Hallmark gene sets “Glycolysis” and “MTORC1 Signaling” in the Cell metabolism group; Hallmark gene sets “TNFα signaling via NFkB”, “TGF Beta signaling”, and “Inflammatory response” in the Inflammation group. In total, *N* = 284 leading edge genes were extracted and annotated with the corresponding gene group. If a gene belonged to more than one group, the gene group label was appended with ‘multiple’. Pearson’s correlation coefficient was calculated between all pairs of genes and the resulting matrix was analyzed with tSNE^93^ to visualize the network, where each node corresponds to a gene. Correlations between pairs of genes with *p*-value < 0.001 are shown with a gray edge, and the size of each node corresponds to the number of correlations with *p*-value < 0.001.

### Reporting summary

Further information on research design is available in the Nature Portfolio Reporting Summary linked to this article.

## Supplementary information

Supplementary Information

Description of Additional Supplementary Files

Supplementary Data 1

Supplementary Data 2

Supplementary Data 3

Supplementary Data 4

Supplementary Data 5

Supplementary Data 6

Reporting Summary

Transparent Peer Review file

## Source data

Source Data

Source Data - Figure 2b

Source Data - Figure S13a

## Data Availability

The immunome and proteome data generated in this study have been deposited in the Dryad database under accession code 10.5061/dryad.z8w9ghxst and in the Research Data Repository of the University of Hamburg: https://www.fdr.uni-hamburg.de/record/18834. The scRNAseq data generated in this study can be accessed via the DDBJ database under the BioProject: PRJDB42605 and have also been deposited in the Research Data Repository of the University of Hamburg: https://www.fdr.uni-hamburg.de/record/18030. The data visualized in this study are provided in the Source Data file. Source data are provided with this paper.

## Code availability

The code generated in this study has been deposited on Zenodo under accession code 10.5281/zenodo.20337723 and on GitHub: https://github.com/joshuagi/multiomics_ptb_public.git.
