# Supplementary material for: Immunological maladaptation preceding spontaneous preterm birth in human pregnancies
Source: Nat Commun. 2026 Jul 27;17:7121. doi: 10.1038/s41467-026-75605-5 (PMC13408597; doi:10.1038/s41467-026-75605-5)
Supplement: Supplementary file 1 — Supplementary Information [file 41467_2026_75605_MOESM1_ESM.pdf]

Immunological Maladaptation Preceding Spontaneous Preterm Birth in Human Pregnancies

SUPPLEMENTARY INFORMATION

Authors: Ina A. Stelzer[1,2,#], Joshua Gillard[1,3,#], Christopher Urbschat[4,5,#], Kristin Thiele[4,5,#], Dorien Feyaerts[1], Mirja Pagenkemper[4], Ann-Christin Tallarek[4], Bettina Hollwitz[4], Masaki Sato[1], Edward Ganio[1], Maïgane Diop[1], Kazuo Ando[1], Jakob Einhaus[1], Nima Aghaeepour[1,6,7], David K. Stevenson[6], Nicola Gagliani[5,8], Anna Woestemeier[8], Stefan Bonn[5,9,10], Anke Diemert[4,10,‡], Petra C. Arck[4,5,10, ‡*], Brice Gaudillière[1, ‡*]

Affiliations:

[1]Department of Anesthesiology, Perioperative and Pain Medicine, Stanford University, Stanford, CA, USA

[2]Department of Pathology, University of California San Diego, La Jolla, CA, USA

[3]Department of Medicine, Division of Cardiovascular Medicine, Stanford University, Stanford, CA, USA

[4]Department of Obstetrics and Prenatal Medicine, University Medical Center Hamburg-Eppendorf, Germany

[5]Hamburg Center for Translational Immunology, University Medical Center Hamburg-Eppendorf, Germany

[6]Department of Pediatrics, Stanford University, Stanford, CA, USA

[7] Department of Biomedical Data Science, Stanford University, Stanford, CA, USA

[8]Department for General, Visceral, Thoracic Surgery, University Medical Center Hamburg-Eppendorf, Germany

[9]Institute of Medical Systems Bioinformatics, Center for Biomedical AI (bAIome), Center for Molecular Neurobiology Hamburg (ZMNH), University Medical Center Hamburg-Eppendorf, Germany

[10]German Center for Child and Adolescent Health, Hamburg, Germany

[#] These authors contributed equally to this work

[‡] These authors contributed equally to this work

*Co-corresponding authors: Petra Arck, MD (email: p.arck@uke.de)
Brice Gaudillière, MD/PhD (email: gbrice@stanford.edu)

Content:

Supplementary Figures 1-16
Supplementary Tables 1-7

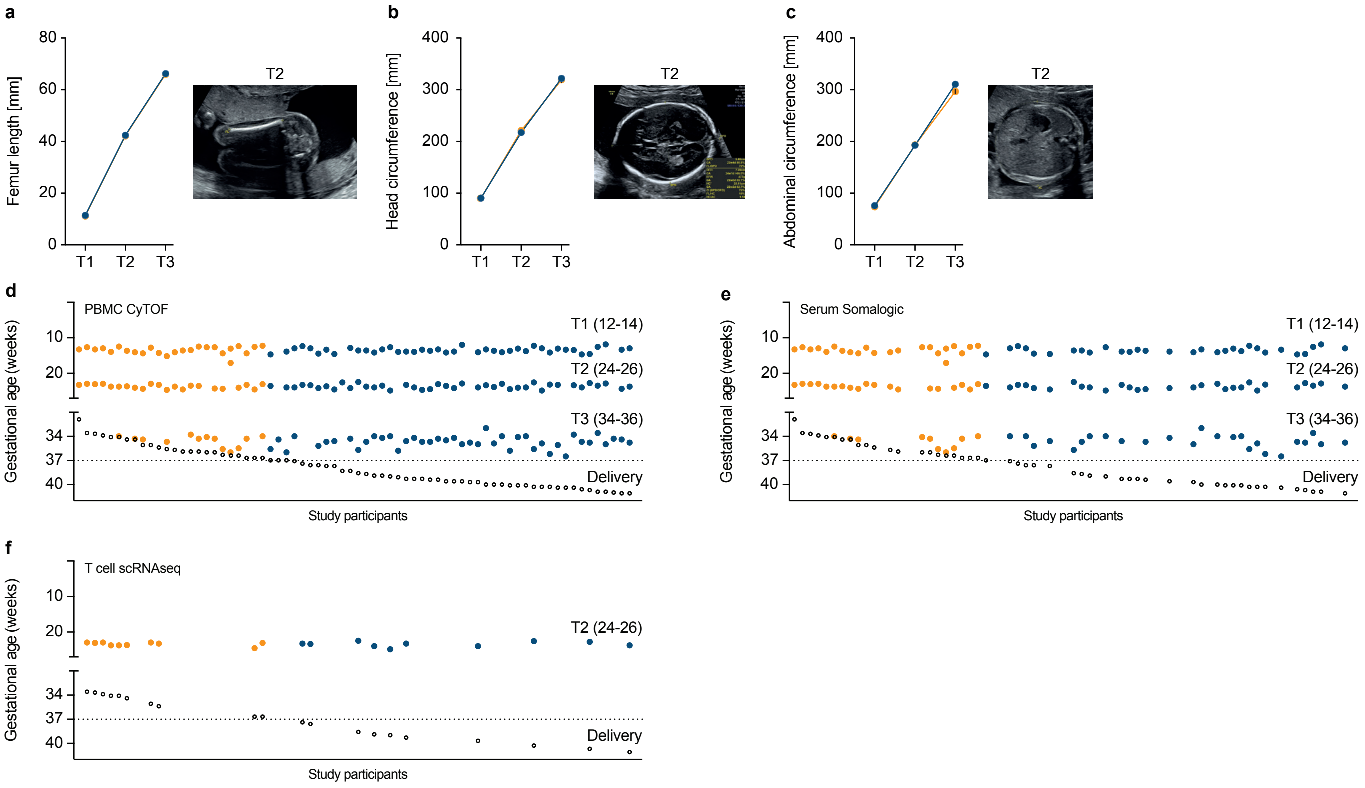

Supplementary Fig. 1: Assessment of the maternal immunome, proteome, transcriptome were performed in spontaneous preterm birth (sPTB) and term birth (TB) pregnancies. (a–c) Ultrasound measurements of fetal parameters reveal no differences in fetal growth across pregnancy. Inserts (right) show representative ultrasound images of the respective measurement at T2. (d-f) Sample cohorts of PBMC for CyTOF analyses (d, sPTB: N = 24, n = 60, TB: N = 46, n = 126), serum for aptamer-based proteomics (Somalogic, e, sPTB: N = 21, n = 51, TB: N = 30, n = 84), and T cells for single-cell RNA sequencing (f, sPTB: N = 10, n = 10, TB: N = 10, n = 10). x-axis shows gestational age (GA, weeks) at sampling. y-axis depicts a single patient per row. Participant N and sample n are indicated per group. Source data are provided as a Source Data file.

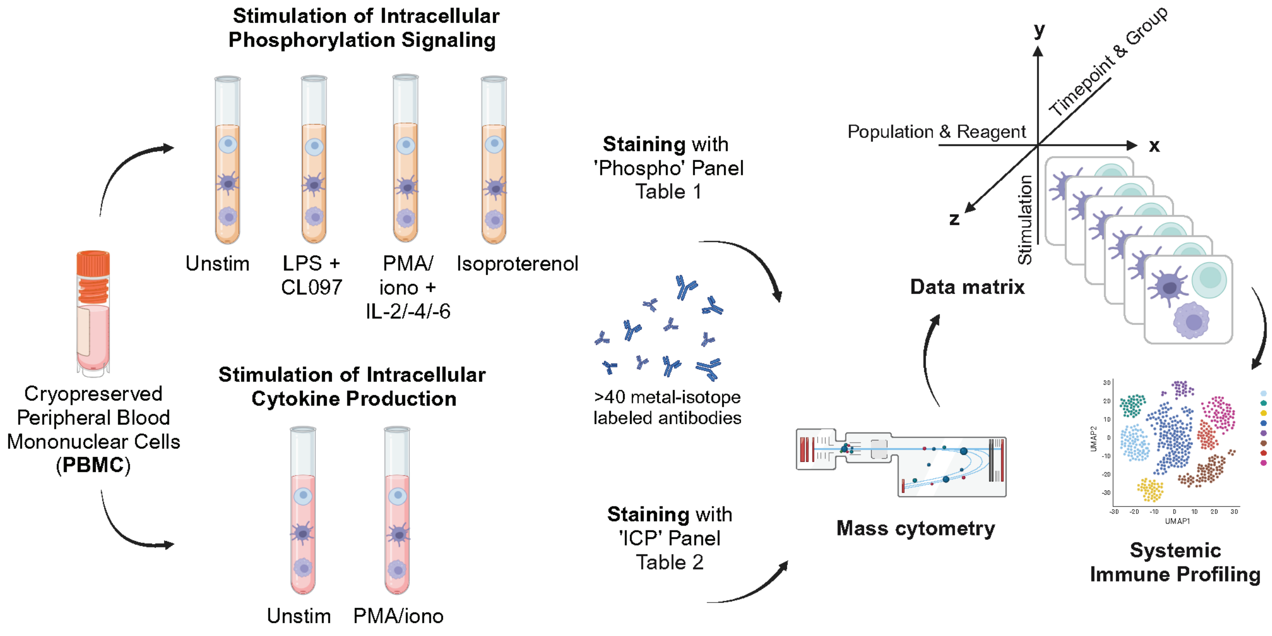

Supplementary Fig. 2: Overview of in vitro PBMC stimulation assay for single-cell mass cytometry. Maternal blood immune cells (PBMC) were stimulated with a selection of agents to either measure intracellular phosphorylation signaling or intracellular cytokine production (Methods), fixed, barcoded, and stained with panels of 40+ antibodies targeting extra- and intracellular markers defining phenotype and function across adaptive and innate compartments (Supplementary Tables 1 and 2). The samples were acquired on a Helios Mass Cytometer (Standard Biotools Inc.). The resulting .fcs files were manually gated to generate a data matrix across groups, timepoints, stimulations, populations, and reagents (Supplementary Fig. 3) that served as the input for the immune profiling task to define immunopathobiology preceding sPTB (Fig. 2, Fig. 5). Figure created in BioRender. Stelzer, I. (2026) https://BioRender.com/ywu08mj

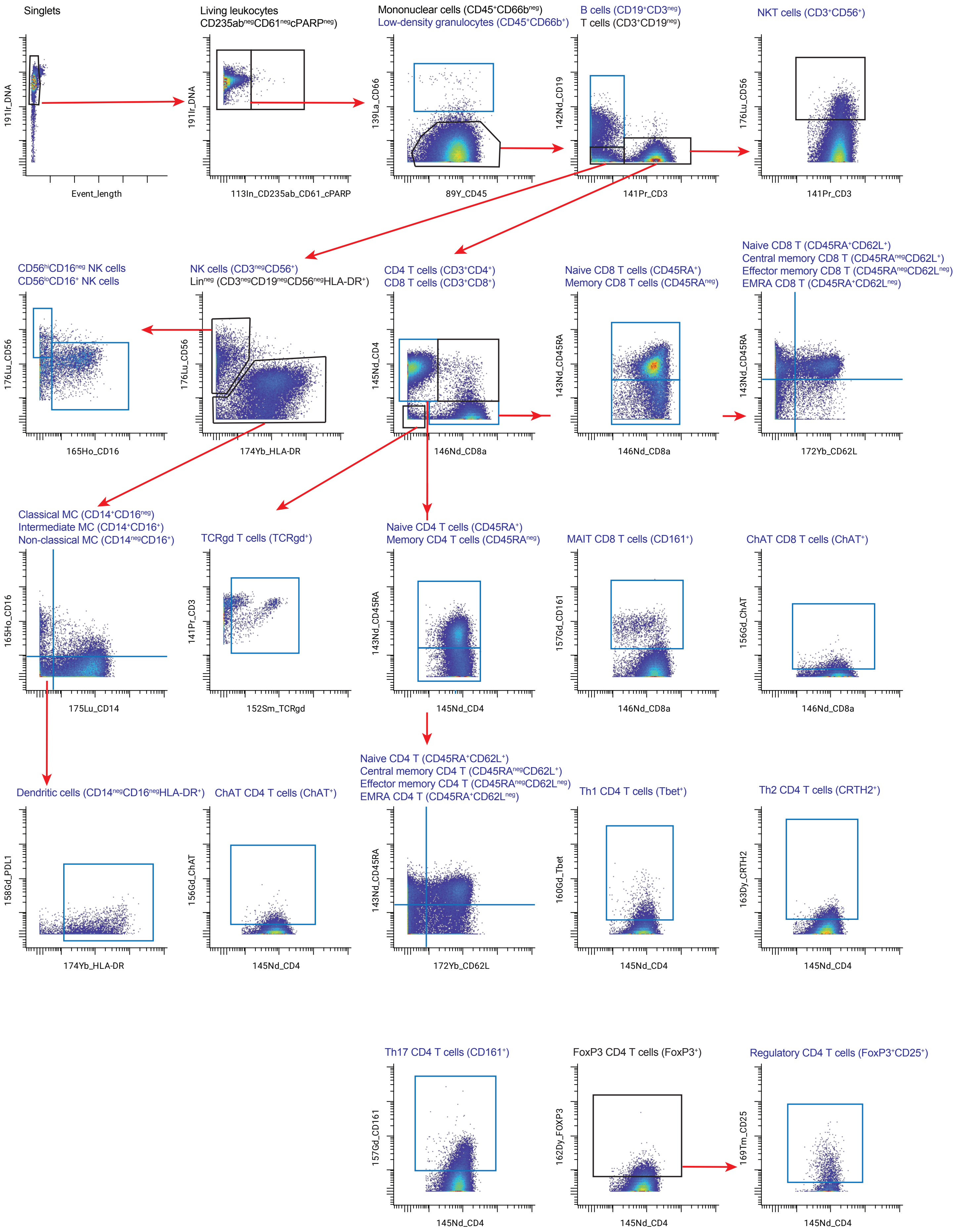
Singlets
191Ir_DNA
Event_length
Living leukocytes
CD235ab^neg CD61^neg cPARP^neg
191Ir_DNA
113In_CD235ab_CD61_cPARP
Mononuclear cells (CD45+CD66b^neg)
Low-density granulocytes (CD45+CD66b+)
139La_CD66
89Y_CD45
B cells (CD19+CD3^neg)
T cells (CD3+CD19^neg)
142Nd_CD19
141Pr_CD3
NKT cells (CD3+CD56+)
176Lu_CD56
141Pr_CD3
CD56^hi CD16^neg NK cells
CD56^lo CD16+ NK cells
176Lu_CD56
165Ho_CD16
NK cells (CD3^neg CD56+)
Lin^neg (CD3^neg CD19^neg CD56^neg HLA-DR+)
176Lu_CD56
174Yb_HLA-DR
CD4 T cells (CD3+CD4+)
CD8 T cells (CD3+CD8+)
145Nd_CD4
146Nd_CD8a
Naive CD8 T cells (CD45RA+)
Memory CD8 T cells (CD45RA^neg)
143Nd_CD45RA
146Nd_CD8a
Naive CD8 T (CD45RA+CD62L+)
Central memory CD8 T (CD45RA^neg CD62L+)
Effector memory CD8 T (CD45RA^neg CD62L^neg)
EMRA CD8 T (CD45RA+CD62L^neg)
143Nd_CD45RA
172Yb_CD62L
Classical MC (CD14+CD16^neg)
Intermediate MC (CD14+CD16+)
Non-classical MC (CD14^neg CD16+)
165Ho_CD16
175Lu_CD14
TCRgd T cells (TCRgd+)
141Pr_CD3
152Sm_TCRgd
Naive CD4 T cells (CD45RA+)
Memory CD4 T cells (CD45RA^neg)
143Nd_CD45RA
145Nd_CD4
MAIT CD8 T cells (CD161+)
157Gd_CD161
146Nd_CD8a
ChAT CD8 T cells (ChAT+)
156Gd_ChAT
146Nd_CD8a
Dendritic cells (CD14^neg CD16^neg HLA-DR+)
158Gd_PDL1
174Yb_HLA-DR
ChAT CD4 T cells (ChAT+)
156Gd_ChAT
145Nd_CD4
Naive CD4 T (CD45RA+CD62L+)
Central memory CD4 T (CD45RA^neg CD62L+)
Effector memory CD4 T (CD45RA^neg CD62L^neg)
EMRA CD4 T (CD45RA+CD62L^neg)
143Nd_CD45RA
172Yb_CD62L
Th1 CD4 T cells (Tbet+)
160Gd_Tbet
145Nd_CD4
Th2 CD4 T cells (CRTH2+)
163Dy_CRTH2
145Nd_CD4
Th17 CD4 T cells (CD161+)
157Gd_CD161
145Nd_CD4
FoxP3 CD4 T cells (FoxP3+)
162Dy_FOXP3
145Nd_CD4
Regulatory CD4 T cells (FoxP3+CD25+)
169Tm_CD25
145Nd_CD4

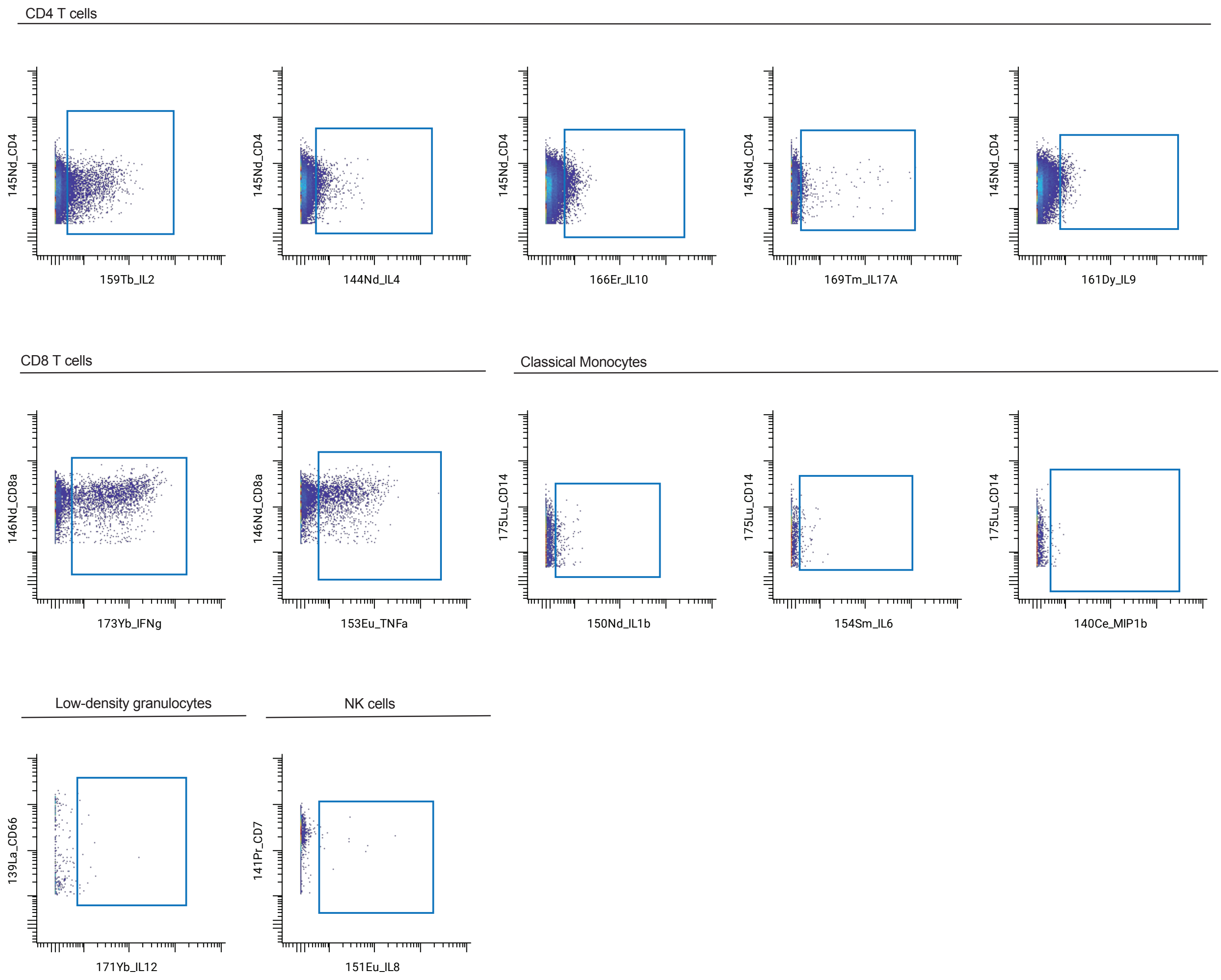

Supplementary Fig. 3: Gating strategy for mass cytometry analyses of intracellular phosphorylation and cytokine production. Singlet, live, non-erythroid cell populations (highlighted with blue squares) were used for analysis.

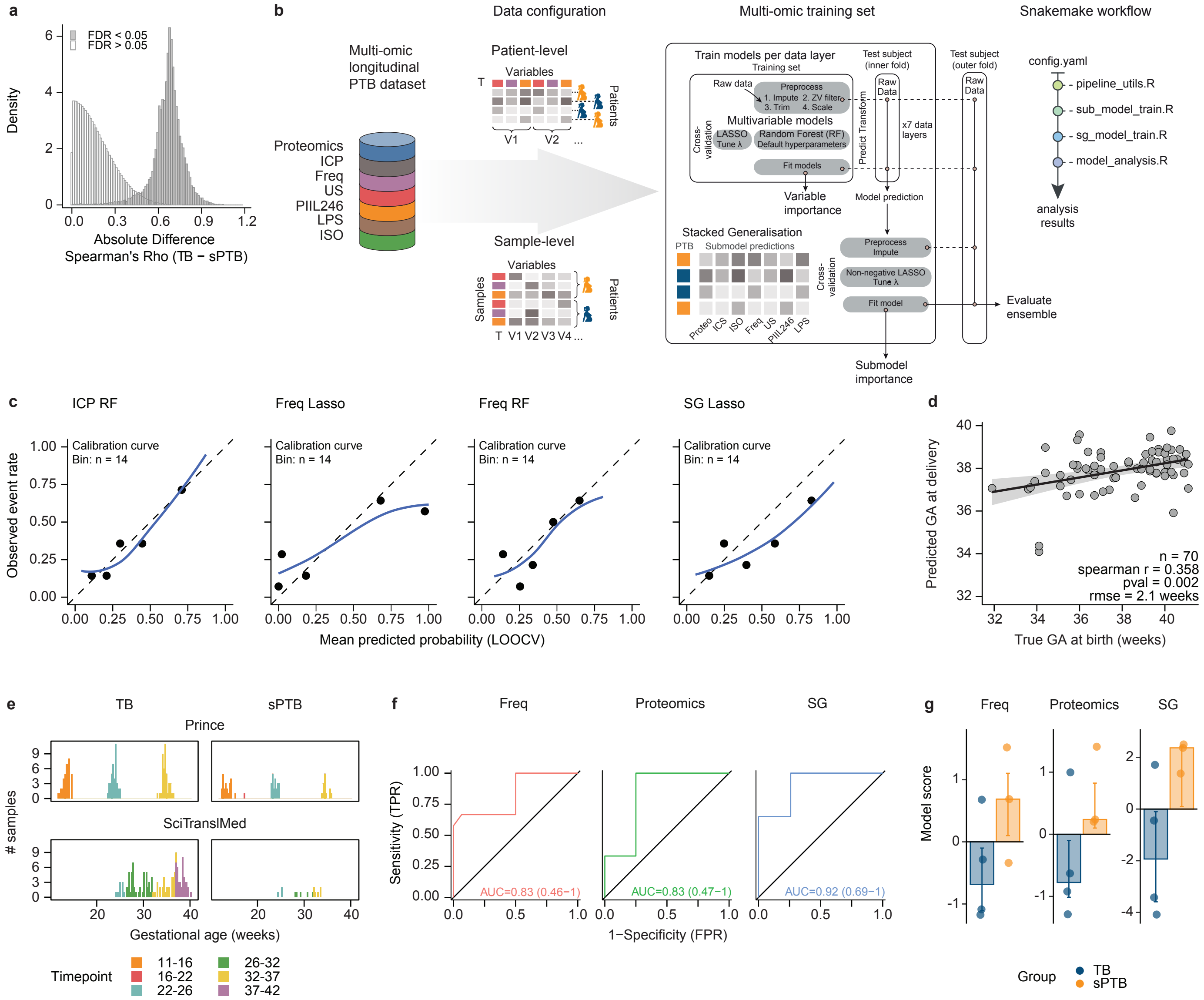

Supplementary Fig. 4: (a) The distribution of correlations that are significantly different (FDR < 0.05) in sPTB compared to TB or not (FDR > 0.05). The absolute difference in Spearman's rho (TB – sPTB) is indicated on the x-axis. (b) Schematic of a multi-omic modeling approach to reveal gestational immune and proteomic trajectories and to identify potential biomarker candidates. The model is built using a patient-level (Fig. 2) or sample-level (Fig. 5) data configuration for distinguishing sPTB from TB (Methods). T: trimester, V: variable, (c) Calibration curves of the top predictive single-omic models and the multi-omic SG model to assess how well predicted risk corresponds to the observed outcomes. Circles on the diagonal indicate a well-calibrated model; circles above or below indicate under- or overconfidence in the estimated risk, respectively. (d) Regression of predicted vs. true gestational age (weeks) at delivery based on all data layers using a linear regression model. (e) Overview of blood samples and measurements in the test (Prince) vs. validation (SciTranslMed) data sets. Histograms depicting the number of TB and sPTB samples with mass cytometry data and proteomics over the course of gestation. Bars are colored according to time periods corresponding to Prince sample timepoints T1, T2, and T3. (f) Results of the multi-omic model predicting sPTB in sPTB with mass cytometry (frequencies) and proteomics data. ROC curve and AUC. (g) Prediction scores, median (IQR) for each participant in TB (N = 4) vs. sPTB (N = 3) groups. Source data are provided as a Source Data file.

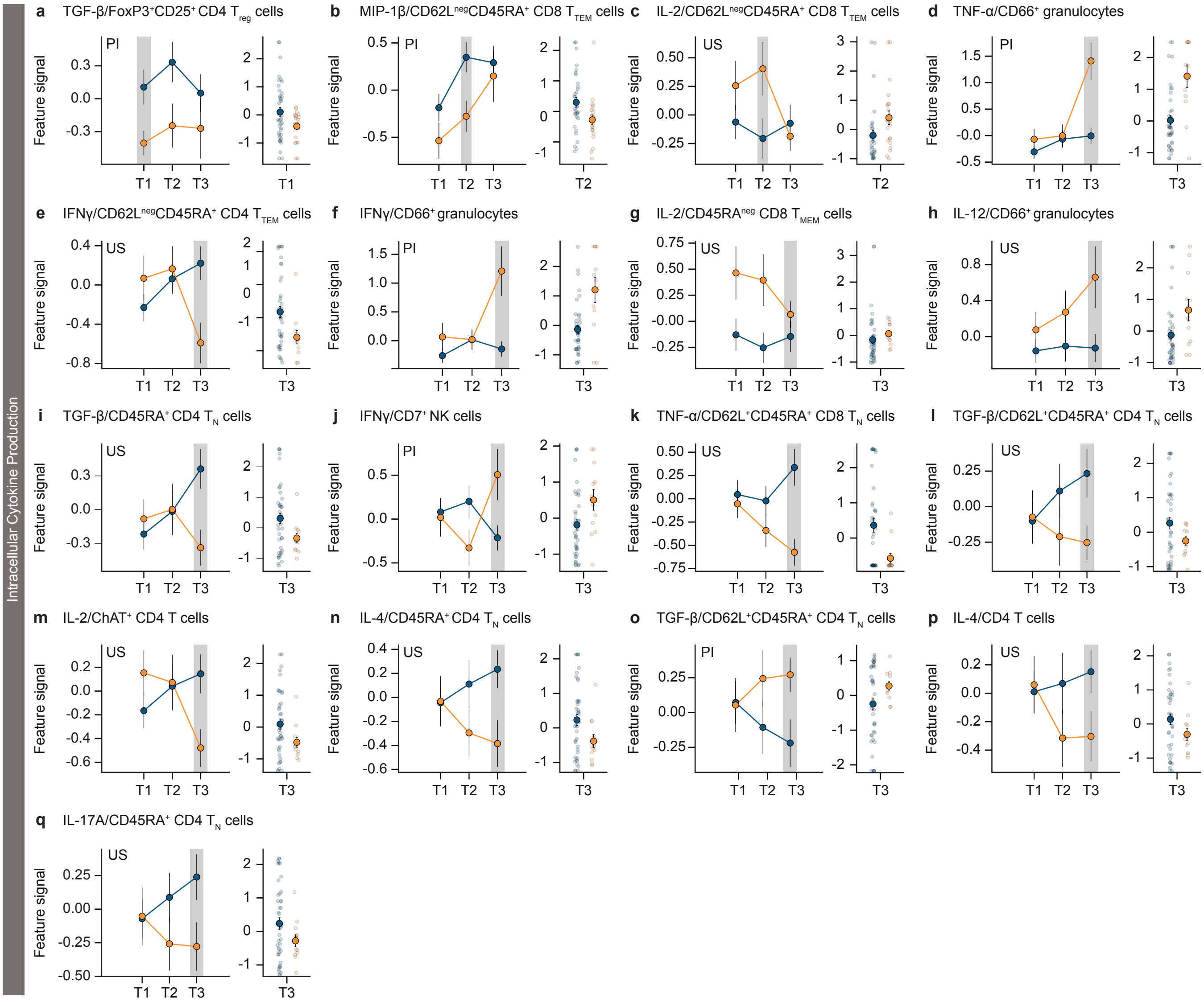

Supplementary Fig. 5: Model features of intracellular cytokine production (ICP) data layer, ranked across model features from all submodels. (a–q) Left: Feature signal across pregnancy with informative timepoint chosen by the model shaded in gray. Right: Comparison of individual patients' values between TB (N = 46, n = 126) and sPTB (N = 24, n = 60) at timepoint chosen by the model. Data are presented as the mean ± SE of winsorized (5th and 95th percentile) and scaled values. Model features of the ICP layer were sorted by timepoint and subsequently by rank. The complete ranking can be reviewed in Supplementary Table 4. US: Unstimulated, PI: Stimulated with PMA/ionomycin (Methods). Treg: regulatory T cells, TEM: terminal effector memory, MEM: memory, N: naive, NK: natural killer cells, IFN: interferon, IL: interleukin, TGF: transforming Growth Factor, TNF: tumor necrosis factor. Source data are provided as a Source Data file.

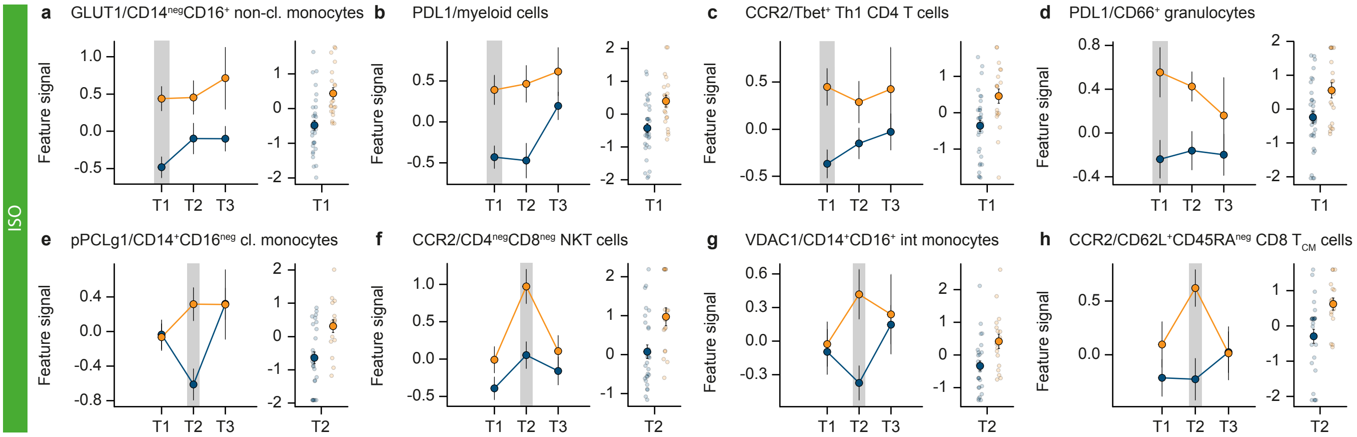

Supplementary Fig. 6: Model features of adrenergic (ISO) response data layer, ranked across model features from all submodels. (a–h) Left: Feature signal across pregnancy with informative timepoint chosen by the model shaded in gray. Right: Comparison of individual patients' values between TB (N = 46, n = 126) and sPTB (N = 24, n = 60) at timepoint chosen by the model. Data are presented as the mean ± SE of winsorized (5th and 95th percentile) and scaled values. Model features of the ISO layer were sorted by timepoint and subsequently by rank. The complete ranking can be reviewed in Supplementary Table 4. Monocyte subpopulations: cl: classical, non-cl: non-classical, int: intermediate; CM: central memory, NKT: natural killer T cells, Th: T helper cells. Source data are provided as a Source Data file.

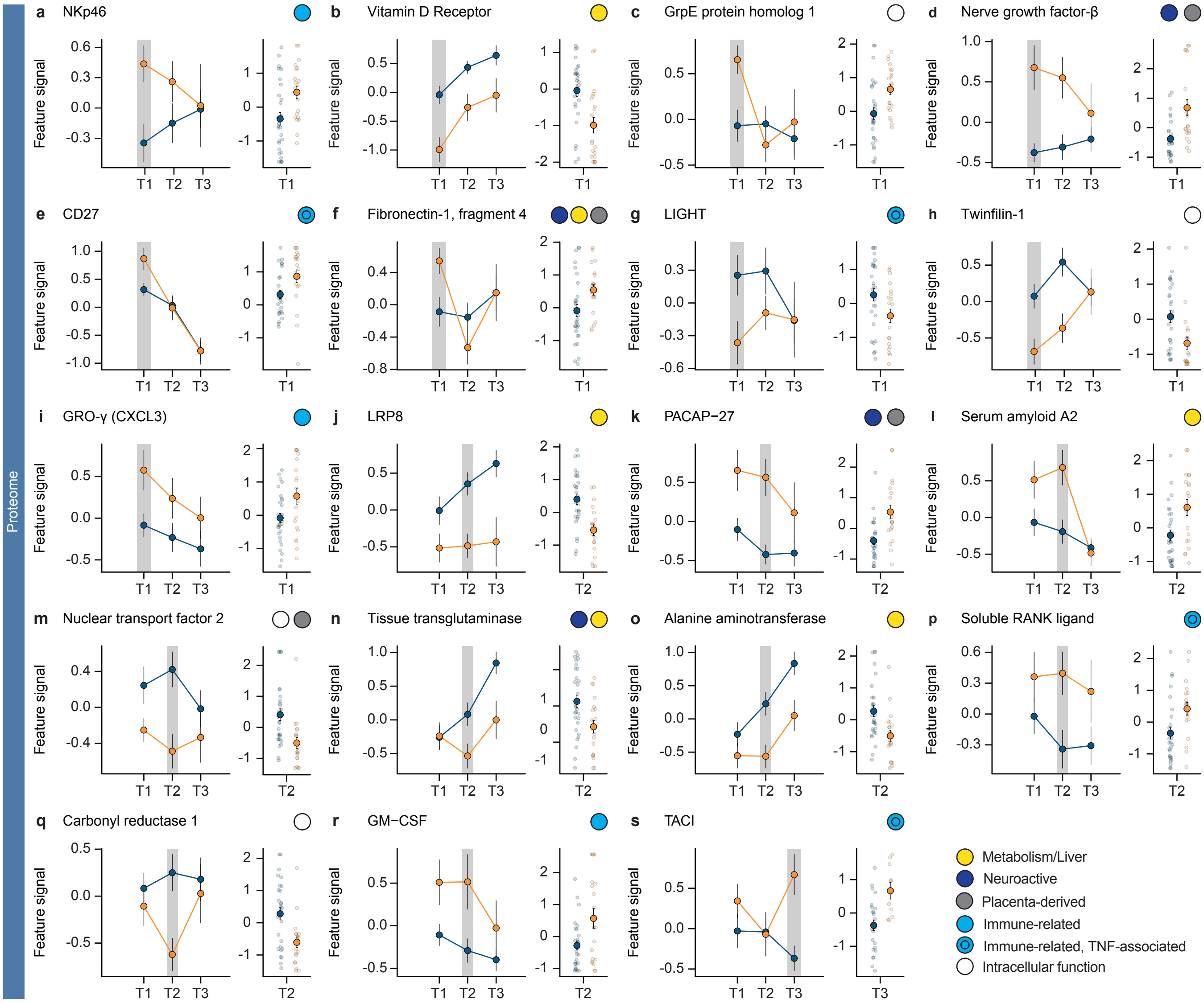

Supplementary Fig. 7: Model features of the proteomic data layer, ranked across model features from all submodels. (a–s) Left: Feature signal across pregnancy with informative timepoint (T: trimester) chosen by the model shaded in gray. Right: Comparison of individual patients' values between TB (N = 30, n = 84) and sPTB (N = 21, n = 51) at timepoint chosen by the model. Data are presented as the mean ± SE of winsorized (5th and 95th percentile) and scaled values. Model features of the proteome layer were sorted by timepoint and subsequently by rank. The complete ranking can be reviewed in Supplementary Table 4. CD27: Tumor necrosis factor receptor superfamily member 7, CXCL3: Chemokine (C-X-C motif) ligand 3, GM-CSF: Granulocyte-macrophage colony-stimulating factor, LIGHT: tumor necrosis factor superfamily member 14, LRP8: Low-density lipoprotein receptor-related protein 8, NKp46: Natural killer cell p46-related protein (Natural cytotoxicity triggering receptor 1), PACAP-27: Pituitary adenylate cyclase-activating polypeptide-27, TACI: Transmembrane activator and CAML interactor. Source data are provided as a Source Data file.

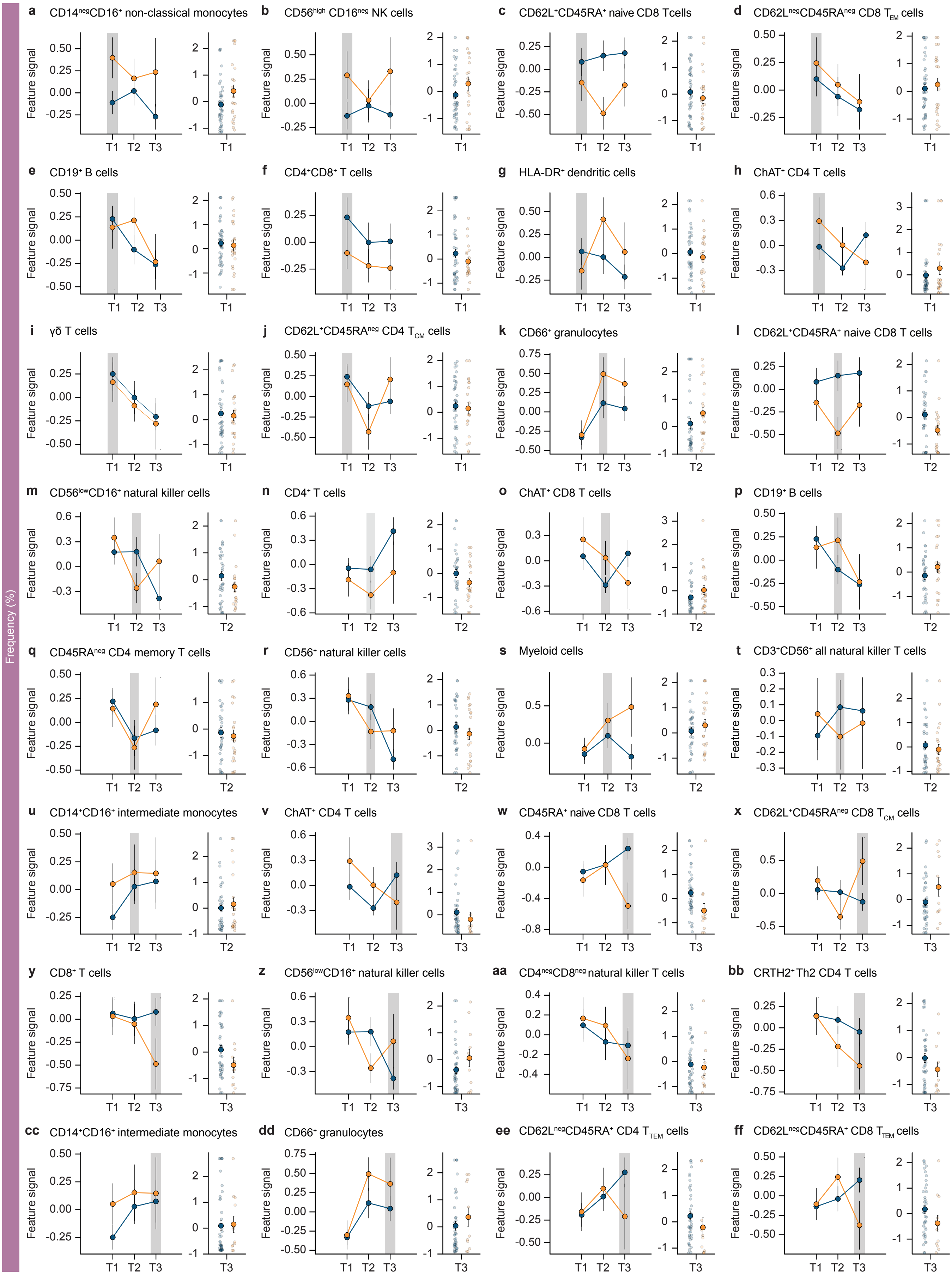

Supplementary Fig. 8: Model features of immune cell population frequencies (Freq) data layer, ranked across model features from all submodels. (a–ff) Left: Feature signal across pregnancy with informative timepoint chosen by the model shaded in gray. Right: Comparison of individual patients' values between TB (N = 46, n = 126) and sPTB (N = 24, n = 60) at timepoint chosen by the model. Data are presented as the mean ± SE of winsorized (5th and 95th percentile) and scaled values. Model features of the Freq layer were sorted by timepoint and subsequently by rank. The complete ranking can be reviewed in Supplementary Table 4. CM: central memory, EM: effector memory, TEM: terminal effector memory. Source data are provided as a Source Data file.

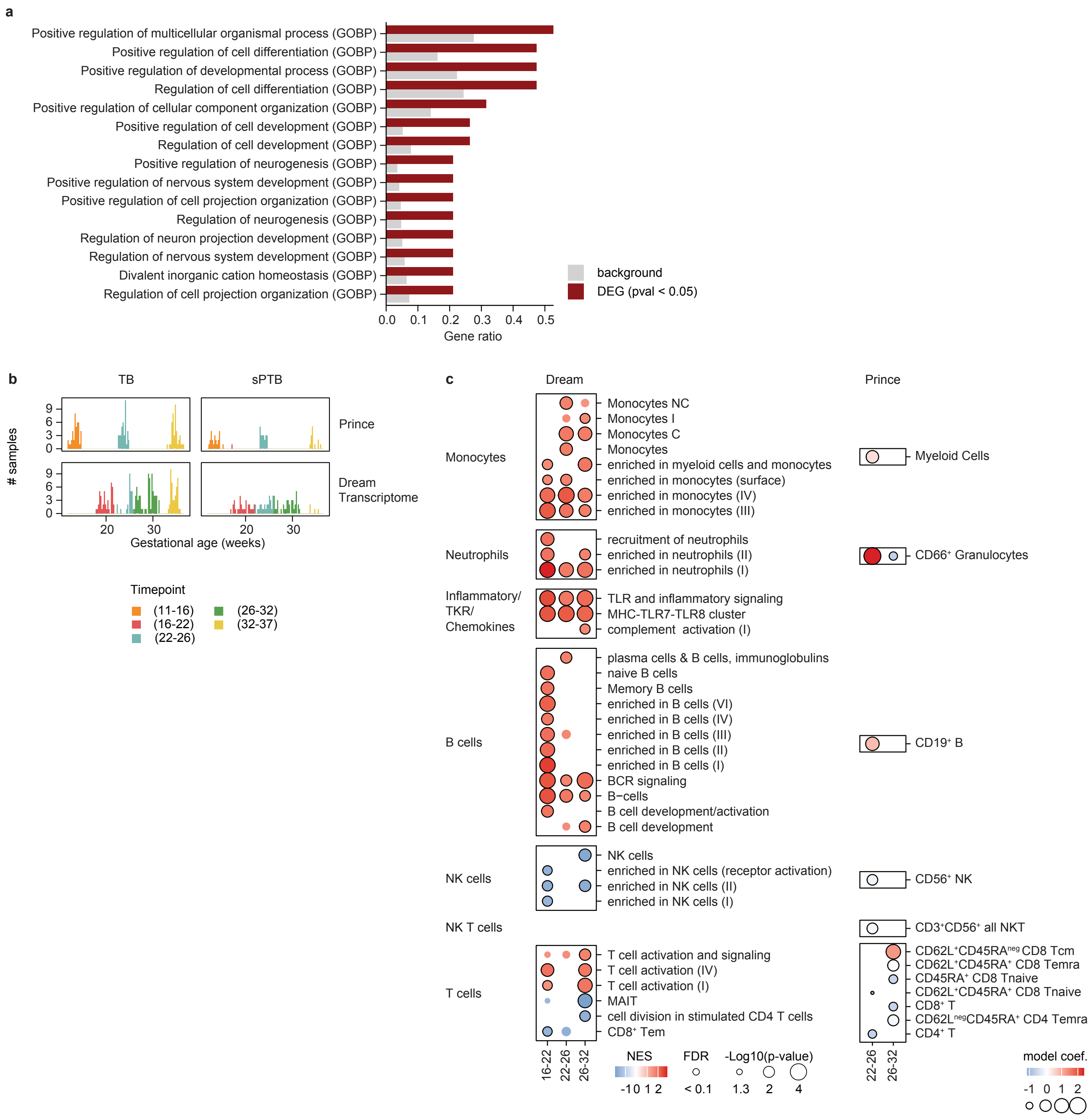

Supplementary Fig. 9: Proteomics pathway enrichment analysis and immune feature validation. (a) Nineteen proteins informing the model were used to perform over-representation analysis of MSigDB Gene Ontology Biological Process gene sets, resulting in enrichment of pathways related to cellular and neural development (Methods, Supplementary Table 8). Statistical significance and p-values were calculated using a one-sided upper-tail hypergeometric test. Shown are pathways with nominal p-value < 0.05 and overlap ≥ 4. GOBP: Gene Ontology Biological Process. (b) Overview of blood samples and measurements in the test (Prince) vs. validation (Dream Study) data sets. Histograms depicting the number of TB and sPTB samples over the course of gestation. Bars are colored according to time periods corresponding to Prince sample timepoints. (c) Left: Gene set enrichment analysis (GSEA) was used to calculate the normalized enrichment score (NES) of blood transcription modules derived from whole blood microarray samples (Dream study) using a list of all genes ranked by the log2-fold difference between sPTB and TB samples. Statistical significance and p-values were calculated against an empirical null distribution and reflect two-sided tests. Selected gene sets are shown (nominal p-value<0.05); and those with FDR < 0.1 highlighted with a black border. On the right y-axis, blood transcription modules are shown for different immune cell populations, each bubble corresponds to a normalized enrichment score (NES) for a module at a timepoint on the x-axis. Right: Bubble plot of the Prince model coefficients in the frequency submodel, i.e., each frequency feature's contribution to the prediction model. Source data are provided as a Source Data file.

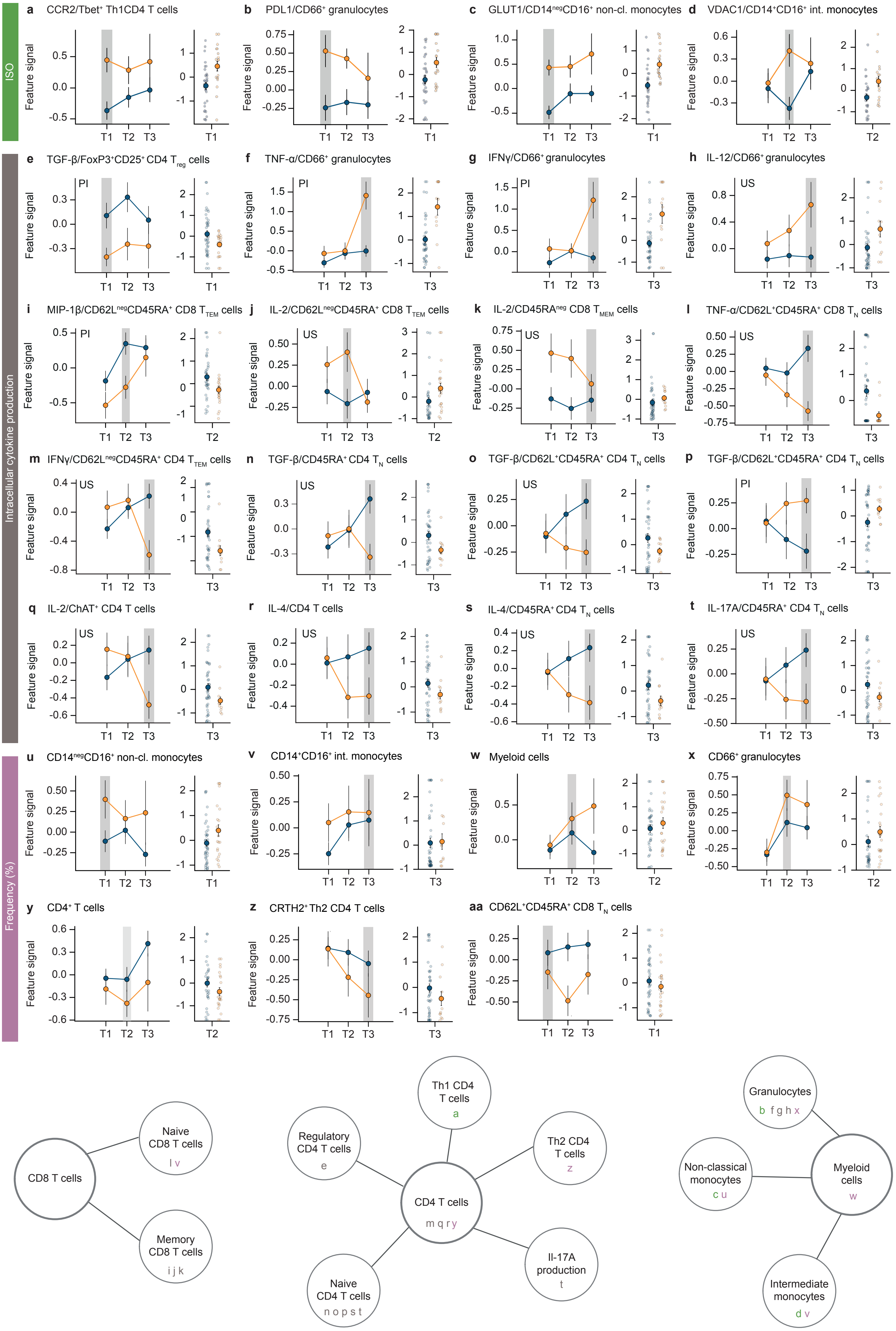

Supplementary Fig. 10: Key immune model features across populations and stimulation conditions. Immune features selected from Supplementary Figures 5, 7, 8 representing the major trajectories of immunological maladaptation preceding sPTB vs. TB. Both CD4 T cells and myeloid cells contribute significantly to the immunopathobiology of sPTB. Monocyte (MC) subpopulations: non-cl: non-classical, int: intermediate; Th: T helper cells, Treg: regulatory T cells, MEM: memory, TEM: terminal effector memory, N: naive

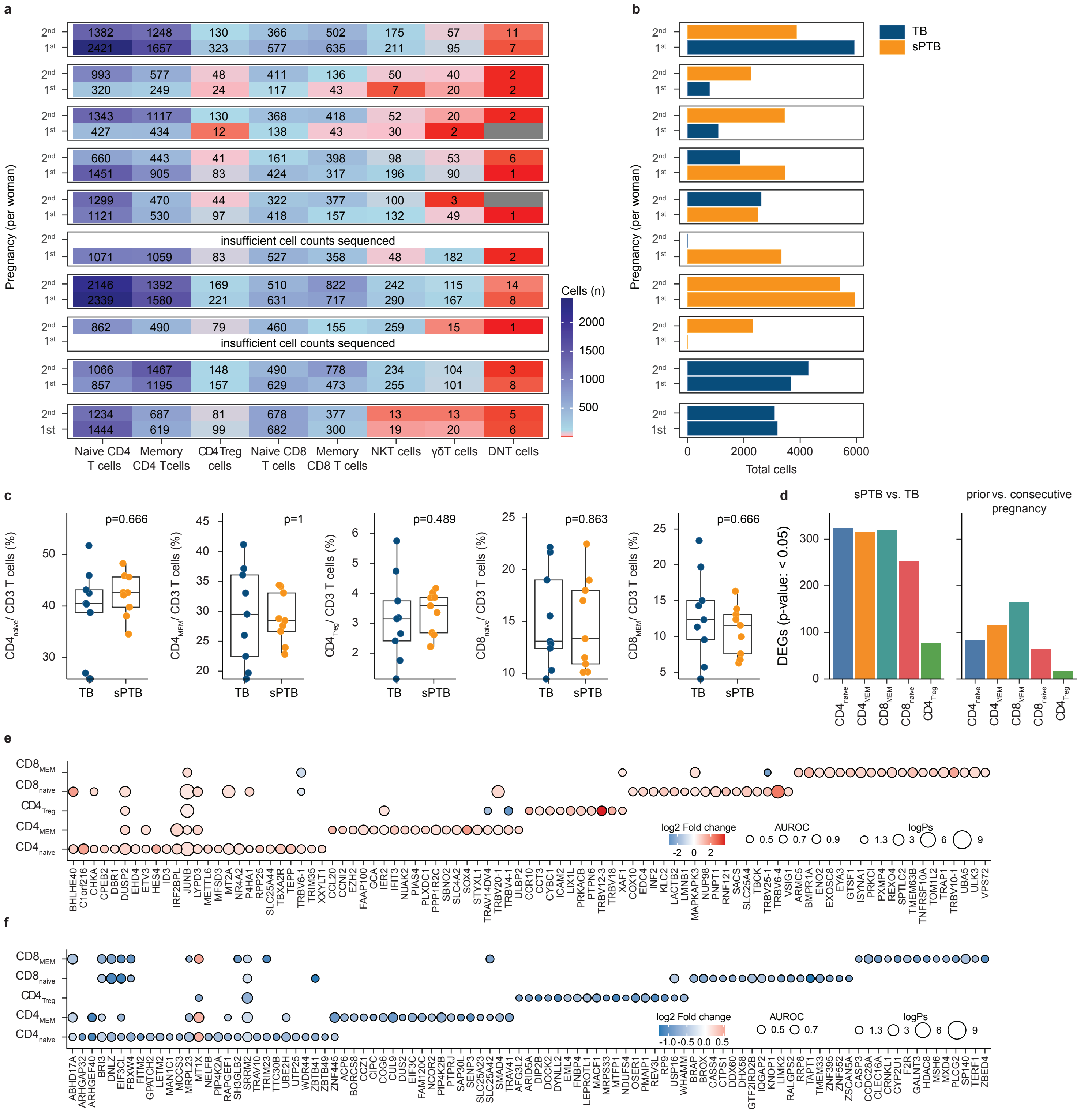

Supplementary Fig. 11: Aggregation of single cells into pseudobulk samples, differential abundance analysis and differential gene expression analysis. (a,b) Overview of the transcriptome subcohort. Mothers (N = 10) participated twice in the study (total pregnancies, N = 18), experiencing either (1) two TB (N = 2), (2) two sPTB (N = 2), and (3) first a TB, then a sPTB (N = 3), or vice versa (N = 3). (a) Heat map of the number of cells contributed by each patient for each immune cell population. (b) Total number of cells of each patient sample (TB = blue, sPTB = yellow). (c) The frequencies of the indicated T cell populations among total T cells (Wilcox test, p-values are displayed in the graphs). Data are presented as a box plots, with bounds from 25th to 75th percentile, median line, and whiskers, which extend to the largest or smallest value no further than 1.5 * the inter-quartile range. (d) The number of differentially expressed genes (DEGs) for each immune cell population when comparing sPTB vs. TB (left) and prior vs. consecutive pregnancy (right). (e, f) Dot plots of the top (e) upregulated and (f) downregulated DEGs in sPTB for each population. Source data are provided as a Source Data file.

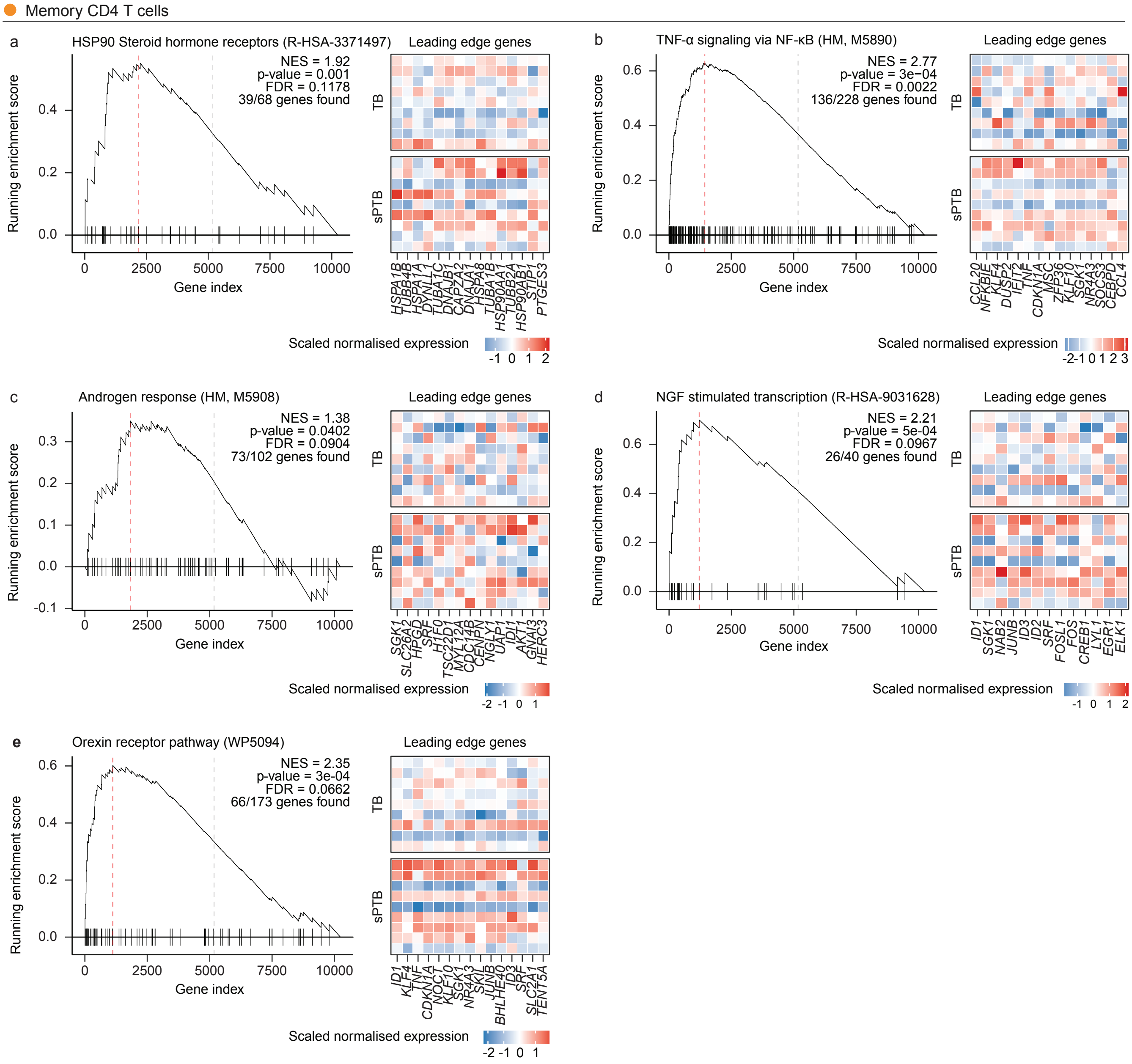

Supplementary Fig. 12: Enrichment plots of selected gene sets for memory CD4 T cells. (a–e) Left panel: Gene set enrichment plots for the indicated pathways. NES, normalized enrichment score. The nominal p-value of the enrichment with false-discovery rate-corrected (FDR) p-value is shown, together with the total number of genes in the pathway with detectable expression in memory CD4 T cells (genes found). Right panel: Heatmap of the scaled normalized expression of leading-edge genes for T cells of each sample (y-axis), separated into TB and sPTB groups. Source data are provided as a Source Data file.

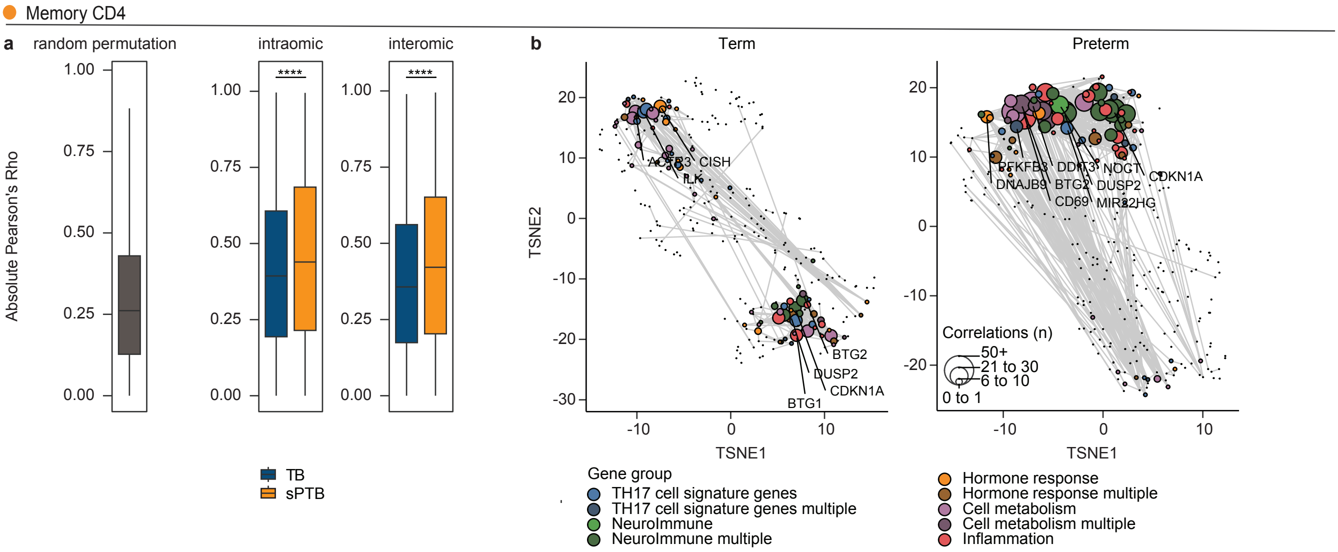

Supplementary Fig. 13: Transcriptional correlation network in TB vs. sPTB memory CD4 cells. (a) Inter- and intra-pathway correlation of genes expressed in a select set of pathways overexpressed in the CD4 memory signature of sPTB, compared to random permutation. Leading edge genes of enriched representative gene sets were extracted (Methods) and pairwise Pearson correlations were calculated within TB and sPTB memory CD4 T cell samples. Data are presented as a box plots, with bounds from 25th to 75th percentile, median line, and whiskers, which extend to the largest or smallest value no further than 1.5 * the inter-quartile range. (b) A t-SNE map of genes was constructed with the resulting correlation matrices, where each point corresponds to a gene. Gray lines indicate significant correlations ($p < 0.001$). The size and color of each point indicate the number of significant correlations and the corresponding gene group for each gene. The gene in each gene group with the largest number of correlations is shown. This visualization exemplified the tight clustering of sPTB genes within and across DE-pathways as compared to increased modularity and decreased number of correlations in individual clusters in TB. Source data are provided as a Source Data file.

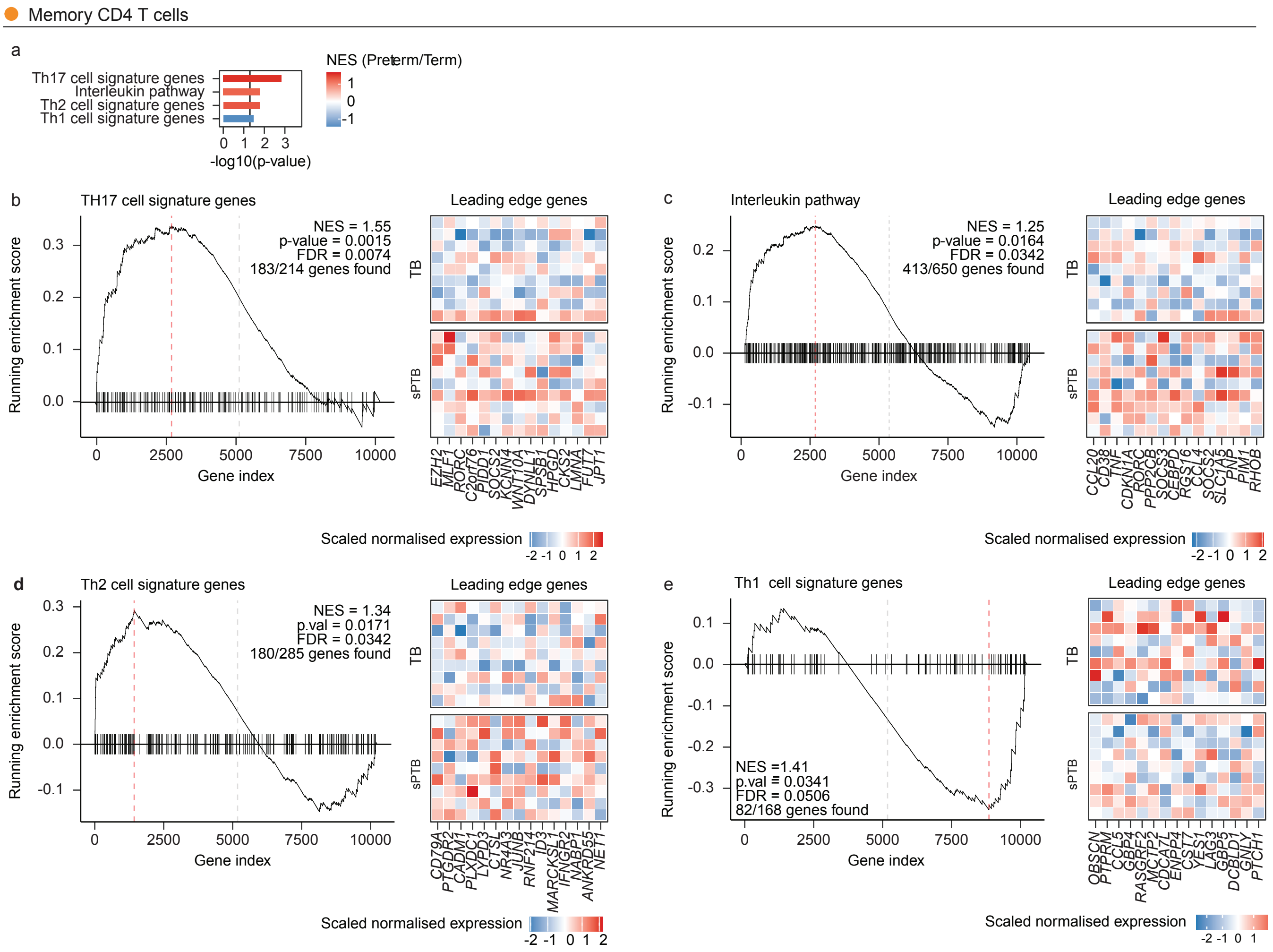

Supplementary Fig. 14: Enrichment plots of selected gene sets for memory CD4 T cells. (a) GSEA results (FDR < 0.1) of selected Th signatures reported in Seumois et al, analyzed in CD4 memory T cells. The color of each bar corresponds to the NES and -log10(nominal p-value) is shown on the x-axis. The black vertical line corresponds to p = 0.05.(b–e) Left panel: Gene set enrichment plots for the indicated pathways. NES, normalized enrichment score. The nominal p-value of the enrichment with false-discovery rate-corrected (FDR) p-value is shown, together with the total number of genes in the pathway with detectable expression in memory CD4 T cells (genes found). Right panel: Heatmap of the scaled normalized expression of leading-edge genes for T cells of each sample (y-axis), separated into TB and sPTB groups. Source data are provided as a Source Data file.

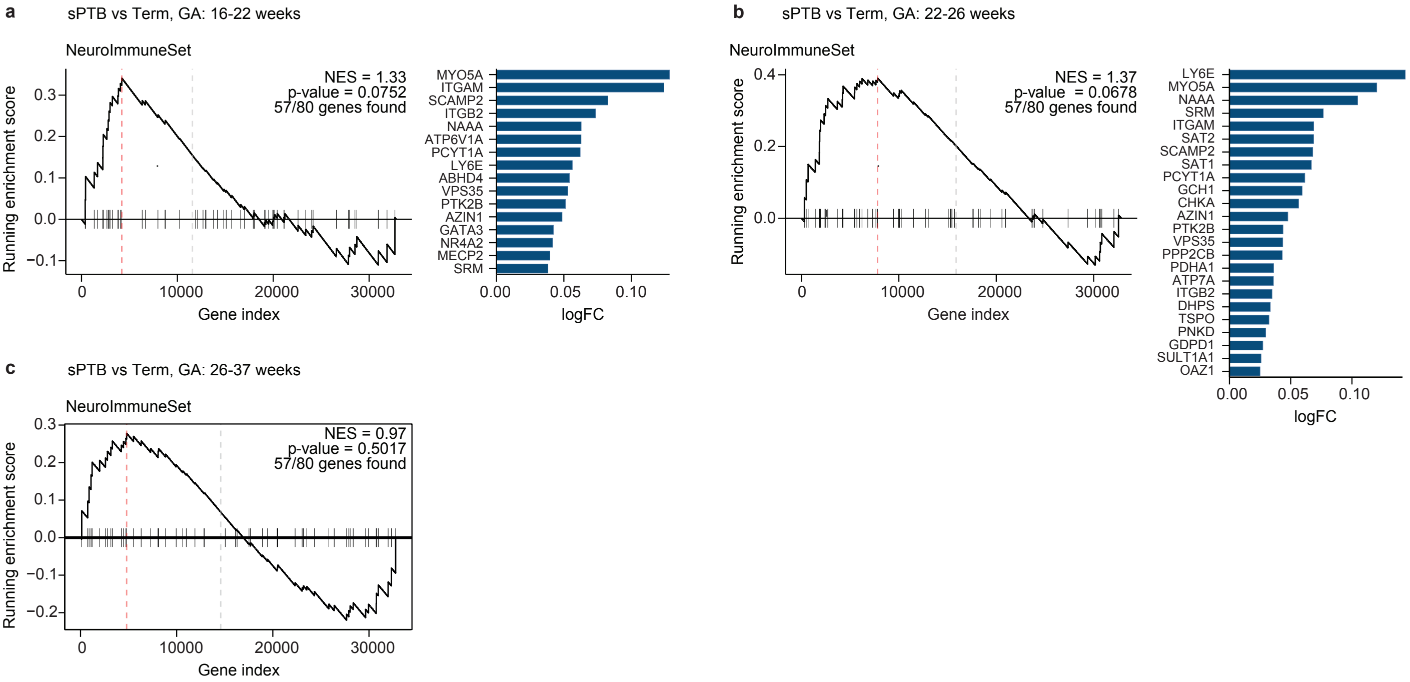

Supplementary Fig. 15: External validation of neuroimmune transcriptome signatures in sPTB vs. TB. Whole blood microarray (bulk transcriptome) re-analysis (Dream study) trends towards recapitulating single-T cell transcriptional profiles of neuroimmune gene set expression in second trimester sPTB vs. TB. (a) Gestational week 16-22, (b) 22-26 and (c) 26-37. Left: Gene set enrichment plots for the neuroimmune gene set. NES, normalized enrichment score. The nominal p-value of the enrichment is shown, together with the total number of genes in the pathway with detectable expression in bulk transcriptome (genes found). Right panel: Log foldchange (logFC) of leading-edge genes differentially expressed in sPTB vs. TB. Source data are provided as a Source Data file.

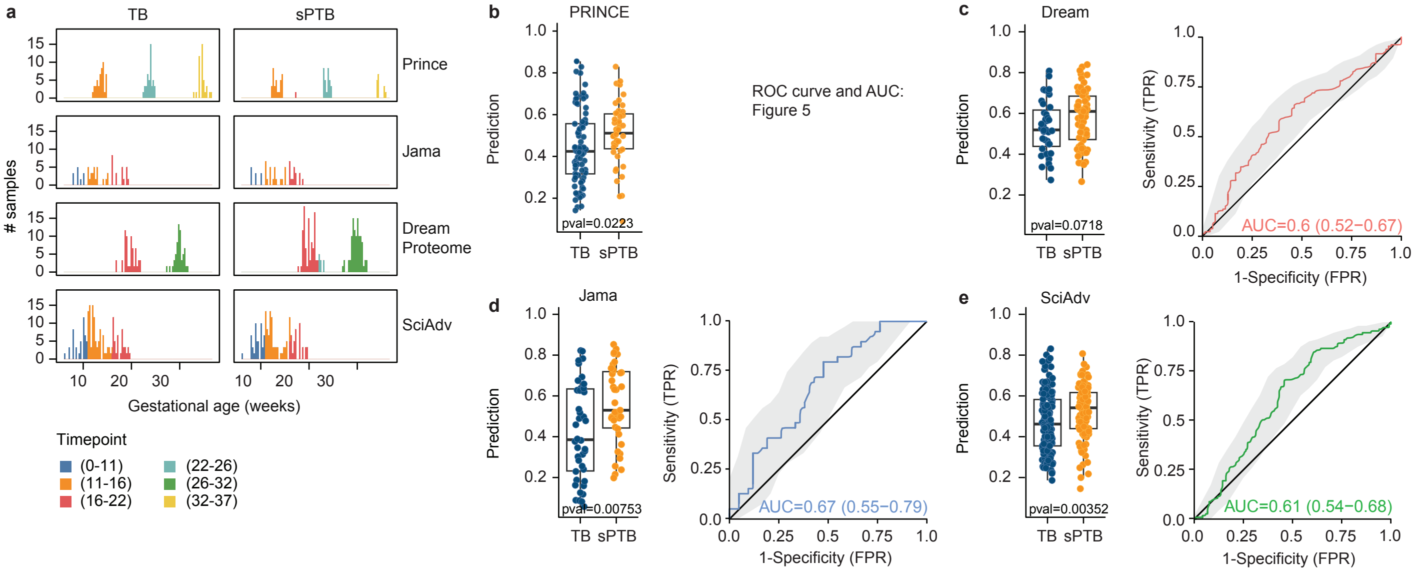

Supplementary Fig. 16: A core set of circulating proteomic markers predicts PTB. (a) Overview of blood samples and measurements in the Prince study vs. published data sets. Histograms depicting the number of Term and Preterm samples with proteomics data of external cohorts over the course of gestation. Bars are colored according to time periods corresponding to Prince sample timepoints T1, T2, and T3. (b) Prediction score of the sample-level stacked multiomic model distinguishing sPTB from TB using data from T1 and T2 time points of the Prince dataset. The p-value above corresponds to a Wilcox test of prediction scores. Data are presented as box plots, with bounds from the 25th to the 75th percentile, median line, and whiskers extending to the largest or smallest value no further than 1.5 times the inter-quartile range. (c–e) Results of the random forest model predicting preterm birth with proteomic data in the (c) Dream, (d) Jama, and (e) SciAdv datasets. Left panel: prediction scores for each participant in TB vs. sPTB groups. The p-value above corresponds to Wilcox test of prediction scores. Right panel: ROC curve and AUC. Source data are provided as a Source Data file.

Supplementary Table 1: Mass cytometry panel for intracellular phosphorylation (Phospho)

Antibody	Manufacturer	Atomic Symbol	Atomic Mass	Clone	Comment	Staining	Catalogue Number	RRID
CD45	Biolegend	Y	89	HI30	Phenotype	Surface	304045	AB_2562821
Barcode 1	Trace Sciences	Pd	102		Barcode	Intracellular		
Barcode 2	Trace Sciences	Pd	104		Barcode	Intracellular		
Barcode 3	Trace Sciences	Pd	105		Barcode	Intracellular		
Barcode 4	Trace Sciences	Pd	106		Barcode	Intracellular		
Barcode 5	Trace Sciences	Pd	108		Barcode	Intracellular		
Barcode 6	Trace Sciences	Pd	110		Barcode	Intracellular		
CD235	BioLegend	In	113	HIR2	Phenotype	Surface	306615	AB_2562825
CD61	BD	In	113	VI-PL2	Phenotype	Surface	555752	AB_396093
cPARP	BD	In	113	F21-852	Apoptosis	Intracellular	552596	AB_394437
CCR5	Biolegend	In	115	J418F1	Chemotaxis	Surface	359102	AB_2562456
CD66	BD	La	139	B1.1/CD66	Phenotype	Surface	551354	AB_394166
Total H3	CST	Ce	140	D1H2	Epigenetics	Intracellular	4499	AB_10544537
CD3	BioLegend	Pr	141	UCHT1	Phenotype	Surface	300402	AB_314056
CD19	BioLegend	Nd	142	HIB19	Phenotype	Surface	302247	AB_2562815
CD45RA	BioLegend	Nd	143	HI100	Phenotype	Surface	304143	AB_2562822
VDAC1	Abcam	Nd	144	20B12AF2	Metabolism	Intracellular	ab14734	AB_443084
CD4	BioLegend	Nd	145	RPA-T4	Phenotype	Surface	300541	AB_2562809
CD8a	BioLegend	Nd	146	RPA-T8	Phenotype	Surface	301053	AB_2562810
GLUT1	Abcam	Sm	147	EPR3915	Metabolism	Surface	ab252403	
CPT1a	Abcam	Nd	148	8F6AE9	Metabolism	Surface	ab128568	AB_11141632
pCREB (pS133)	CST	Sm	149	87G3	Signaling	Intracellular	9198	AB_2561044
pSTAT5 (Tyr694)	CST	Nd	150	C11C5	Signaling	Intracellular	9359	AB_823649
pP38 (pT180/pY18	BD	Eu	151	36/p38	Signaling	Intracellular	612289	AB_399606
TCRγδ	BD	Sm	152	B1	Phenotype	Surface	555715	AB_396059
pSTAT1 (pY701)	BD	Eu	153	14/P-STAT1	Signaling	Intracellular	612133	AB_399504
pSTAT3 (Tyr705)	CST	Sm	154	M9C6	Signaling	Intracellular	4113	AB_2198588
pS6 (pS235/pS236	CST	Gd	155	D57.2.2E	Signaling	Intracellular	4858	AB_916156
ChAT	Abcam	Gd	156	EPR13024(B)	Function	Intracellular	ab224267	
CD161	Biolegend	Gd	157		Phenotype	Surface	339919	AB_2562836
PD-L1	Biolegend	Gd	158	29E.2A3	Function	Surface	329719	AB_2565429
H3K27me3	Active Motif	Tb	159	MABI 0323	Epigenetics	Intracellular	61017	AB_2614987
Tbet	Thermo Fisher	Gd	160	4B10	Phenotype	Intracellular	14-5825-80	AB_763635
Ki67	BD	Dy	161	B56	Proliferation	Intracellular	556003	AB_396287
FoxP3	Thermo Fisher	Dy	162	PCH101	Phenotype	Intracellular	14-4776-82	AB_467554

CRTH2	Biolegend	Dy	163	BM16	Phenotype	Surface	350102	AB_10639863
CD44	Biolegend	Dy	164	BJ16	Function	Surface	338811	AB_2562835
CD16	BioLegend	Ho	165	3G8	Phenotype	Surface	302051	AB_2562814
pNFκB (pS529)	BD	Er	166	K10-895.12.50	Signaling	Intracellular	558393	AB_647284
HSP90ab	Biolegend	Yb	167	K3720A	Function	Intracellular	661802	AB_2564179
CD40L	Biolegend	Er	168	24-31	Function	Surface	310802	AB_314825
CD25	BioLegend	Tm	169	M-A251	Phenotype	Surface	356102	AB_2561752
pPLCg1	Biolegend	Er	170	A17025A	Signaling	Intracellular	612402	AB_2810659
pERK1/2 (pT202/p'	CST	Yb	171	D13.14.4E	Signaling	Intracellular	4370	AB_2315112
CD62L	Thermo Scientific	Yb	172	DREG.200	Phenotype	Surface	BMS1015	AB_10596353
HK2	Abcam	Yb	173	EPR20839	Metabolism	Intracellular	ab228819	AB_2868547
HLA-DR	BioLegend	Yb	174	L243	Phenotype	Surface	307651	AB_2562826
CD14	BioLegend	Yb	175	M5E2	Phenotype	Surface	301843	AB_2562813
CD56	BD	Yb	176	NCAM16.2	Phenotype	Surface	559043	AB_397180
DNA1	Standard Biotools	Ir	191		DNA	Intracellular		
DNA2	Standard Biotools	Ir	192		DNA	Intracellular		
CCR2	BioLegend	Bi	209	K036C2	Chemotaxis	Surface	357202	AB_2561851

Supplementary Table 2: Mass cytometry panel for intracellular cytokine production (ICP)

Antibody	Manufacturer	Atomic Symbol	Atomic Mass	Clone	Comment	Staining	Catalogue Number	RRID
CD45	Biolegend	Y	89	HI30	Phenotype	Surface	304045	AB_2562821
Barcode 1	Trace Sciences	Pd	102		Barcode	Intracellular		
Barcode 2	Trace Sciences	Pd	104		Barcode	Intracellular		
Barcode 3	Trace Sciences	Pd	105		Barcode	Intracellular		
Barcode 4	Trace Sciences	Pd	106		Barcode	Intracellular		
Barcode 5	Trace Sciences	Pd	108		Barcode	Intracellular		
Barcode 6	Trace Sciences	Pd	110		Barcode	Intracellular		
CD235	BioLegend	In	113	HIR2	Phenotype	Surface	306615	AB_2562825
CD61	BD	In	113	VI-PL2	Phenotype	Surface	555752	AB_396093
cPARP	BD	In	113	F21-852	Function	Intracellular	552596	AB_394437
CCR5	Biolegend	In	115	J418F1	Chemotaxis	Surface	359102	AB_2562456
CD66	BD	La	139	B1.1/CD66	Phenotype	Surface	551354	AB_394166
MIP1b	R&D	Ce	140	24006	Cytokine	Intracellular	MAB271	AB_2071178
CD7	BD	Pr	141	M-T701	Phenotype	Surface	555359	AB_395762
CD19	BioLegend	Nd	142	HIB19	Phenotype	Surface	302247	AB_2562815
CD45RA	BioLegend	Nd	143	HI100	Phenotype	Surface	304143	AB_2562822
IL-4	Biolegend	Nd	144	MP4-25D2	Cytokine	Intracellular	500802	AB_315121
CD4	BioLegend	Nd	145	RPA-T4	Phenotype	Surface	300541	AB_2562809
CD8a	BioLegend	Nd	146	RPA-T8	Phenotype	Surface	301053	AB_2562810
GLUT1	Abcam	Sm	147	EPR3915	Metabolism	Surface	ab252403	
CPT1a	Abcam	Nd	148	8F6AE9	Metabolism	Surface	ab128568	AB_11141632
CD25	BioLegend	Sm	149	M-A251	Phenotype	Surface	356102	AB_2561752
IL-1b	BD	Nd	150	AS10	Cytokine	Intracellular	550007	AB_2124482
IL-8	R&D	Eu	151	6217	Cytokine	Intracellular	MAB208	AB_2249110
TCRγδ	BD	Sm	152	B1	Phenotype	Surface	555715	AB_396059
TNFa	Biolegend	Eu	153	Mab11	Cytokine	Intracellular	502902	AB_315253
IL-6	Biolegend	Sm	154	MQ2-13A5	Cytokine	Intracellular	501115	AB_2562841
TGFb	Biolegend	Gd	155	O92B5	Cytokine	Intracellular	846802	AB_2629662
ChAT	Abcam	Gd	156	EPR13024(B)	Function	Intracellular	ab224267	
CD161	Biolegend	Gd	157		Phenotype	Surface	339919	AB_2562836
PD-L1	Biolegend	Gd	158	29E.2A3	Function	Surface	329719	AB_2565429
IL-2	Biolegend	Tb	159	MQ1-17H12	Cytokine	Intracellular	500302	AB_315088
Tbet	Thermo Fisher	Gd	160	4B10	Phenotype	Intracellular	14-5825-80	AB_763635
IL-9	Biolegend	Dy	161	MH9A4	Cytokine	Intracellular	507602	AB_315484
FoxP3	Thermo Fisher	Dy	162	PCH101	Phenotype	Intracellular	14-4776-82	AB_467554

CRTH2	Biolegend	Dy	163	BM16	Phenotype	Surface	350102	AB_10639863
CD44	Biolegend	Dy	164	BJ16	Function	Surface	338811	AB_2562835
CD16	BioLegend	Ho	165	3G8	Phenotype	Surface	302051	AB_2562814
IL-10	Biolegend	Er	166	JES3-9D7	Cytokine	Intracellular	501505	AB_2125386
HSP90ab	Biolegend	Yb	167	K3720A	Function	Intracellular	661802	AB_2564179
CD40L	Biolegend	Er	168	24-31	Function	Surface	310802	AB_314825
IL-17A	Biolegend	Tm	169	BL168	Cytokine	Intracellular	512331	AB_2563779
CD3	Biolegend	Er	170	UCHT1	Phenotype	Surface	300402	AB_314056
IL-12	Biolegend	Yb	171	C8.6	Cytokine	Intracellular	508803	AB_2810643
CD62L	Thermo Scientific	Yb	172	DREG.200	Phenotype	Surface	BMS1015	AB_10596353
IFNg	Biolegend	Yb	173	B27	Cytokine	Intracellular	506521	AB_2562849
HLA-DR	BioLegend	Yb	174	L243	Phenotype	Surface	307651	AB_2562826
CD14	BioLegend	Yb	175	M5E2	Phenotype	Surface	301843	AB_2562813
CD56	BD	Yb	176	NCAM16.2	Phenotype	Surface	559043	AB_397180
DNA1	Standard Biotools	Ir	191		DNA	Intracellular		
DNA2	Standard Biotools	Ir	192		DNA	Intracellular		
CCR2	BioLegend	Bi	209	K036C2	Chemotaxis	Surface	357202	AB_2561851

Supplementary Table 3: Model comparison

Model	AUROC	Sensitivity	Specificity	DeLong_p
SG Lasso	0.70 (0.57-0.83)	54.2%	84.8%	-
Freq Lasso	0.73 (0.60-0.85)	66.7%	82.6%	0.729
ICP RF	0.75 (0.62-0.89)	75.0%	69.6%	0.363
Freq RF	0.71 (0.58-0.85)	66.7%	80.4%	0.865

Supplementary Table 4: Confounder analysis for sPTB classification model

Parameter	Estimate	Std..Error	z.value	Pr(>|z|)	Model
Predictive accuracy	2,3123764250	0,7663095138	3,0175488930	0,0025482793	Freq_Lasso
Age	-0,0171978842	0,1065027947	-0,1614782430	0,8717167460	Freq_Lasso
BMI	0,0435061905	0,0731547034	0,5947148773	0,5520340755	Freq_Lasso
Gravidity	0,4354176359	0,4992200453	0,8721958182	0,3831015604	Freq_Lasso
Parity	-0,3017662213	0,7624283528	-0,3957961692	0,6922554031	Freq_Lasso
History of prior PTB	-0,5853579775	1,1617848880	-0,5038436837	0,6143712276	Freq_Lasso
Infant sex	0,5298363553	0,6304624520	0,8403931965	0,4006879625	Freq_Lasso
Predictive accuracy	5,1961608060	1,5101887680	3,4407359630	0,0005801343	ICS_RF
Age	0,0854912758	0,1118294542	0,7644790581	0,4445818118	ICS_RF
BMI	0,0854637988	0,0736474601	1,1604446190	0,2458678296	ICS_RF
Gravidity	-0,0805048992	0,5291225667	-0,1521479224	0,8790702676	ICS_RF
Parity	-0,4639422510	0,7676449563	-0,6043708713	0,5455970974	ICS_RF
History of prior PTB	0,6076788774	1,1112479730	0,5468436317	0,5844861644	ICS_RF
Infant sex	0,7257633682	0,6432852591	1,1282138960	0,2592295968	ICS_RF
Predictive accuracy	4,2789146230	1,9767521940	2,1646186290	0,0304169032	ISO_Lasso
Age	0,0441286075	0,1033084898	0,4271537376	0,6692673546	ISO_Lasso
BMI	0,0899938607	0,0728208644	1,2358252190	0,2165235386	ISO_Lasso
Gravidity	0,3832169670	0,4861794634	0,7882212142	0,4305673223	ISO_Lasso
Parity	-0,6294740348	0,7374950863	-0,8535298018	0,3933655683	ISO_Lasso
History of prior PTB	-0,1877316078	1,0885662330	-0,1724576807	0,8630777288	ISO_Lasso
Infant sex	0,8383861834	0,6114529085	1,3711377800	0,1703320098	ISO_Lasso
Predictive accuracy	3,5703612010	1,3872089250	2,5737732340	0,0100596182	Proteomics_Lasso
Age	0,0234384520	0,1040828916	0,2251902460	0,8218312771	Proteomics_Lasso
BMI	0,0709538793	0,0696541156	1,0186602570	0,3083642865	Proteomics_Lasso
Gravidity	0,3812104801	0,5009875520	0,7609180680	0,4467060060	Proteomics_Lasso
Parity	-0,6397806362	0,7845684447	-0,8154554781	0,4148116393	Proteomics_Lasso
History of prior PTB	-0,8310461749	1,2595507910	-0,6597956835	0,5093849537	Proteomics_Lasso
Infant sex	0,6921309160	0,6275780723	1,1028602600	0,2700878543	Proteomics_Lasso
Predictive accuracy	3,1461886520	1,1938721180	2,6352811200	0,0084067626	SG_Lasso

Age	0,0335488628	0,1037801575	0,3232685670	0,7464918570	SG_Lasso
BMI	0,0944641146	0,0730690799	1,2928055850	0,1960783084	SG_Lasso
Gravidity	0,2680518837	0,4990175358	0,5371592468	0,5911576189	SG_Lasso
Parity	-0,5090764096	0,7558463373	-0,6735183919	0,5006175520	SG_Lasso
History of prior PTB	-0,3465092330	1,1242104220	-0,3082245337	0,7579114854	SG_Lasso
Infant sex	0,6602708667	0,6185899671	1,0673804970	0,2858000515	SG_Lasso

Supplementary Table 5: Multi-omic validation for frequency

Rank	Term	Model	Stimulation	Estimate	Population	Timepoint	Mapped cell type
12	CD66p.Granulocytes_T2	Lasso	freq	2,284130749	CD66p.Granulocytes	T2	Granulocytes
16	CD62LpCD45RAp.CD8Tnaive_T2	Lasso	freq	-1,258079625	CD62LpCD45RAp.CD8Tnaive	T2	CD62LposCD8Tnaive
25	CD45RAp.CD8Tnaive_T3	Lasso	freq	-0,669514274	CD45RAp.CD8Tnaive	T3	CD8Tnaive
36	CD62LpCD45RAn.CD8Tcm_T3	Lasso	freq	1,15847331	CD62LpCD45RAn.CD8Tcm	T3	CD8Tcm
38	CD8p.T_T3	Lasso	freq	-0,696345622	CD8p.T	T3	CD8Tcells
42	CD4p.T_T2	Lasso	freq	-0,735523604	CD4p.T	T2	CD4Tcells
52	CD19p.B_T2	Lasso	freq	0,74321296	CD19p.B	T2	Bcells
59	CD66p.Granulocytes_T3	Lasso	freq	-0,760988905	CD66p.Granulocytes	T3	Granulocytes
62	CD62LnCD45RAp.CD4Temra_T3	Lasso	freq	-0,11792307	CD62LnCD45RAp.CD4Temra	T3	CD4Tem
63	CD56p.NK_T2	Lasso	freq	-0,226912116	CD56p.NK	T2	NK
64	MyeloidCells_T2	Lasso	freq	0,371036647	MyeloidCells	T2	cMCs
69	CD3pCD56p.allNKT_T2	Lasso	freq	-0,16278582	CD3pCD56p.allNKT	T2	NKT
72	CD62LnCD45RAp.CD8Temra_T3	Lasso	freq	-0,030489033	CD62LnCD45RAp.CD8Temra	T3	CD8Tem

Supplementary Table 6: Multi-omic validation for proteomics

Rank	Term	Model	Stimulation	Estimate	Target	Timepoint	UniProt
2	LRP8_T2	Lasso	proteomics	-0,497420417	LRP8	T2	Q14114
3	PACAP-27_T2	Lasso	proteomics	0,449984922	PACAP-27	T2	P18509
13	TACI_T3	Lasso	proteomics	0,198282362	TACI	T3	O14836
43	ALT_T2	Lasso	proteomics	-0,054217932	ALT	T2	P24298
54	sRANKL_T2	Lasso	proteomics	0,029954964	sRANKL	T2	O14788
76	GM-CSF_T2	Lasso	proteomics	0,001940122	GM-CSF	T2	P04141

Supplementary Table 7: Proteomics pathway analysis

Pathway	p-value	adjusted p-value	Overlap	Size	n	k	Gene ratio	Bg ratio	Proteins	GeneSet category
GOBP_POSITIVE_REGULATION_OF_MULTICELLULAR_ORGANISMAL_PROCESS	0.01735597129	1	10	398	1047	19	10/19	398/1445	VDR | TWF1 | sRANKL | Tissue transglutaminase | LRP8 | FN1.4 | GM-CSF | CD27 | b-NG	GO:BP
GOBP_POSITIVE_REGULATION_OF_CELL_DIFFERENTIATION	0.001369237061	1	9	234	1211	19	9/19	234/1445	VDR | sRANKL | Tissue transglutaminase | LRP8 | FN1.4 | GM-CSF | LIGHT | CD27 | b-N	GO:BP
GOBP_POSITIVE_REGULATION_OF_DEVELOPMENTAL_PROCESS	0.01349092801	1	9	323	1122	19	9/19	323/1445	VDR | sRANKL | Tissue transglutaminase | LRP8 | FN1.4 | GM-CSF | LIGHT | CD27 | b-N	GO:BP
GOBP_REGULATION_OF_CELL_DIFFERENTIATION	0.02365217834	1	9	352	1093	19	9/19	352/1445	VDR | sRANKL | Tissue transglutaminase | LRP8 | FN1.4 | GM-CSF | LIGHT | CD27 | b-N	GO:BP
GOBP_POSITIVE_REGULATION_OF_CELLULAR_COMPONENT_ORGANIZATION	0.03960664296	1	6	203	1242	19	6/19	203/1445	TWF1 | LRP8 | FN1.4 | GM-CSF | LIGHT | b-NGF	GO:BP
GOBP_POSITIVE_REGULATION_OF_CELL_DEVELOPMENT	0.00229618384	1	5	76	1369	19	5/19	76/1445	sRANKL | Tissue transglutaminase | LRP8 | FN1.4 | b-NGF	GO:BP
GOBP_REGULATION_OF_CELL_DEVELOPMENT	0.01279415465	1	5	113	1332	19	5/19	113/1445	sRANKL | Tissue transglutaminase | LRP8 | FN1.4 | b-NGF	GO:BP
GOBP_POSITIVE_REGULATION_OF_NEUROGENESIS	0.00360717621	1	4	51	1394	19	4/19	51/1445	Tissue transglutaminase | LRP8 | FN1.4 | b-NGF	GO:BP
GOBP_POSITIVE_REGULATION_OF_NERVOUS_SYSTEM_DEVELOPMENT	0.006523412733	1	4	60	1385	19	4/19	60/1445	Tissue transglutaminase | LRP8 | FN1.4 | b-NGF	GO:BP
GOBP_POSITIVE_REGULATION_OF_CELL_PROJECTION_ORGANIZATION	0.009664158458	1	4	67	1378	19	4/19	67/1445	TWF1 | LRP8 | FN1.4 | b-NGF	GO:BP
GOBP_REGULATION_OF_NEUROGENESIS	0.01018161947	1	4	68	1377	19	4/19	68/1445	Tissue transglutaminase | LRP8 | FN1.4 | b-NGF	GO:BP
GOBP_REGULATION_OF_NEURON_PROJECTION_DEVELOPMENT	0.01304363763	1	4	73	1372	19	4/19	73/1445	TWF1 | LRP8 | FN1.4 | b-NGF	GO:BP
GOBP_REGULATION_OF_NERVOUS_SYSTEM_DEVELOPMENT	0.0210640433	1	4	84	1361	19	4/19	84/1445	Tissue transglutaminase | LRP8 | FN1.4 | b-NGF	GO:BP
GOBP_DIVALENT_INORGANIC_CATION_HOMEOSTASIS	0.02952973848	1	4	93	1352	19	4/19	93/1445	VDR | sRANKL | Tissue transglutaminase | PACAP-27	GO:BP
GOBP_REGULATION_OF_CELL_PROJECTION_ORGANIZATION	0.04365877836	1	4	105	1340	19	4/19	105/1445	TWF1 | LRP8 | FN1.4 | b-NGF	GO:BP
