# Supplementary material for: Immunological maladaptation preceding spontaneous preterm birth in human pregnancies
Source: Nat Commun. 2026 Jul 27;17:7121. doi: 10.1038/s41467-026-75605-5 (PMC13408597; doi:10.1038/s41467-026-75605-5)
Supplement: Supplementary file 2 — Description of Additional Supplementary Files [file 41467_2026_75605_MOESM2_ESM.pdf]

Description of Additional Supplementary Files

Supplementary Data 1: Model features for sPTB classification model

Supplementary Data 2: Gene set enrichment analysis (GSEA) for scRNA sequencing of T cells

Supplementary Data 3: Gene set enrichment analysis (GSEA) for neuro-immune terms

Supplementary Data 4: Model features for sPTB prediction model

Supplementary Data 5: Confounder analysis for sPTB prediction model

Supplementary Data 6: PRINCE Penalization Matrices
