# Supplementary material for: Immunological maladaptation preceding spontaneous preterm birth in human pregnancies
Source: Nat Commun. 2026 Jul 27;17:7121. doi: 10.1038/s41467-026-75605-5 (PMC13408597; doi:10.1038/s41467-026-75605-5)
Supplement: Supplementary file 9 — Reporting Summary [file 41467_2026_75605_MOESM9_ESM.pdf]

nature portfolio

Corresponding author(s): Petra Arck, Brice Gaudilliere

Last updated by author(s): June 03, 2026

Reporting Summary

Nature Portfolio wishes to improve the reproducibility of the work that we publish. This form provides structure for consistency and transparency in reporting. For further information on Nature Portfolio policies, see our Editorial Policies and the Editorial Policy Checklist.

Statistics

For all statistical analyses, confirm that the following items are present in the figure legend, table legend, main text, or Methods section.

n/a	Confirmed	
☐	☒	The exact sample size (*n*) for each experimental group/condition, given as a discrete number and unit of measurement
☐	☒	A statement on whether measurements were taken from distinct samples or whether the same sample was measured repeatedly
☐	☒	The statistical test(s) used AND whether they are one- or two-sided<br>*Only common tests should be described solely by name; describe more complex techniques in the Methods section.*
☐	☒	A description of all covariates tested
☐	☒	A description of any assumptions or corrections, such as tests of normality and adjustment for multiple comparisons
☐	☒	A full description of the statistical parameters including central tendency (e.g. means) or other basic estimates (e.g. regression coefficient) AND variation (e.g. standard deviation) or associated estimates of uncertainty (e.g. confidence intervals)
☐	☒	For null hypothesis testing, the test statistic (e.g. *F*, *t*, *r*) with confidence intervals, effect sizes, degrees of freedom and *P* value noted<br>*Give P values as exact values whenever suitable.*
☒	☐	For Bayesian analysis, information on the choice of priors and Markov chain Monte Carlo settings
☐	☒	For hierarchical and complex designs, identification of the appropriate level for tests and full reporting of outcomes
☐	☒	Estimates of effect sizes (e.g. Cohen's *d*, Pearson's *r*), indicating how they were calculated

Our web collection on statistics for biologists contains articles on many of the points above.

Software and code

Policy information about availability of computer code

Data collection	Mass cytometry: Helios CyTOF, Standard Biotools Inc., Proteomics: Aptamer-based platform (SomaLogic); Cell Sorting: BD Aria Fusion cell sorter (BD Biosciences), Single-cell RAN sequencing: Single Cell 5' gene expression kit (10x Genomics), Novogene sequencing, Psychometric assessments: validated questionnaires for assessment of psychosocial data, Ultrasound: Voluson E8 (General Electric)
Data analysis	Mass cytometry: ImmuneAtlas (CellEngine), Visualization and univariate statistical analyses: Prism v10 (GraphPad), Data matrices: Excel v16 (Microsoft), Multivariate analyses: R Studio, Python (open source), custom algorithms (see Zenodo repository).

For manuscripts utilizing custom algorithms or software that are central to the research but not yet described in published literature, software must be made available to editors and reviewers. We strongly encourage code deposition in a community repository (e.g. GitHub). See the Nature Portfolio guidelines for submitting code & software for further information.

Data

Policy information about availability of data

All manuscripts must include a data availability statement. This statement should provide the following information, where applicable:

- Accession codes, unique identifiers, or web links for publicly available datasets
- A description of any restrictions on data availability
- For clinical datasets or third party data, please ensure that the statement adheres to our policy

Data generated in this study are available on either Dryad (https://doi.org/10.5061/dryad.z8w9ghxst) or in the Research Data Repository of the University of

Hamburg (https://www.fdr.uni-hamburg.de/record/18030). The code generated in this study has been deposited on Zenodo under accession code https://doi.org/10.5281/zenodo.20337723.

Research involving human participants, their data, or biological material

Policy information about studies with human participants or human data. See also policy information about sex, gender (identity/presentation), and sexual orientation and race, ethnicity and racism.

Reporting on sex and gender	The PRINCE study is a prospective longitudinal pregnancy cohort study. Recruitment was restricted to pregnant women. Fetal sex is considered a biological variable.
Reporting on race, ethnicity, or other socially relevant groupings	We report race and ethnicity. As the recruitment took place at a single center, the University Medical Center Hamburg-Eppendorf, Germany, the majority of participants are of Central European descent, i.e., they identify as White and Non-hispanic. Please see Table 1 for demographic details.
Population characteristics	All pregnant women were above the age of 18 years or higher and had a viable singleton pregnancy at gestational week 12–14 at the time of recruitment. The following exclusion criteria were defined: women with chronic infections (HIV, hepatitis B/C), known substance abuse and smoking, multiple pregnancies or pregnancies conceived after assisted reproductive technologies. Assessment of relevant covariables has been described in detail in Diemert et al., J Reprod Immunol 2017, PMID: 28641119.
Recruitment	Pregnant women were recruited in their first trimester (12-14 weeks of gestation).
Ethics oversight	All study subjects signed an informed consent. The study protocol was approved by the ethics committee of the Hamburg Chamber of Physicians (PV3694).

Note that full information on the approval of the study protocol must also be provided in the manuscript.

Field-specific reporting

Please select the one below that is the best fit for your research. If you are not sure, read the appropriate sections before making your selection.

☒ Life sciences ☐ Behavioural & social sciences ☐ Ecological, evolutionary & environmental sciences

For a reference copy of the document with all sections, see nature.com/documents/nr-reporting-summary-flat.pdf

Life sciences study design

All studies must disclose on these points even when the disclosure is negative.

Sample size	Subject N: preterm: N=24, term: N=46. Sample n: blood and serum samples from all three trimester - preterm: n=58, term=118
Data exclusions	Women who experienced iatrogenic PTB (e.g., delivery due to preeclampsia, induction of labor due to fetal distress or growth restriction, N=16) were excluded.
Replication	The multivariable model underwent internal cross-validation. The proteomics submodel was externally validated in three publicly available data sets.
Randomization	Not applicable due to prospective study design.
Blinding	Not applicable due to prospective study design.

Reporting for specific materials, systems and methods

We require information from authors about some types of materials, experimental systems and methods used in many studies. Here, indicate whether each material, system or method listed is relevant to your study. If you are not sure if a list item applies to your research, read the appropriate section before selecting a response.

Materials & experimental systems

n/a	Involved in the study
☐	☒ Antibodies
☒	☐ Eukaryotic cell lines
☒	☐ Palaeontology and archaeology
☒	☐ Animals and other organisms
☐	☒ Clinical data
☒	☐ Dual use research of concern
☒	☐ Plants

Methods

n/a	Involved in the study
☒	☐ ChIP-seq
☐	☒ Flow cytometry
☒	☐ MRI-based neuroimaging

Antibodies

Antibodies used

Two mass cytometry antibody panels were used for phenotyping of immune cell subsets and to detect either intracellular signaling protein phosphorylation (Supplementary Table 1) or intracellular cytokine production (Supplementary Table 2). Antibodies were either obtained preconjugated (Standard Biotools Inc.) or were purchased as purified, carrier-free (no BSA, gelatin) versions, which were then conjugated in-house with trivalent metal isotopes utilizing the MaxPAR antibody conjugation kit (Standard Biotools Inc.).

Validation

All antibodies have been validated for cytometry by the manufacturer. RRIDs, Manufacturer, and Catalog Number for each antibody can be found in Supplementary Tables 1 and 2.
All antibodies used in the analysis were titrated in-house and validated on samples that were processed identically to the samples used in this study.

Clinical data

Policy information about clinical studies
All manuscripts should comply with the ICMJE guidelines for publication of clinical research and a completed CONSORT checklist must be included with all submissions.

Clinical trial registration

This study does not involve a clinical trial.

Study protocol

Note where the full trial protocol can be accessed OR if not available, explain why.

Data collection

Describe the settings and locales of data collection, noting the time periods of recruitment and data collection.

Outcomes

Describe how you pre-defined primary and secondary outcome measures and how you assessed these measures.

Plants

Seed stocks

This study does not include experiments with plants.

Novel plant genotypes

Describe the methods by which all novel plant genotypes were produced. This includes those generated by transgenic approaches, gene editing, chemical/radiation-based mutagenesis and hybridization. For transgenic lines, describe the transformation method, the number of independent lines analyzed and the generation upon which experiments were performed. For gene-edited lines, describe the editor used, the endogenous sequence targeted for editing, the targeting guide RNA sequence (if applicable) and how the editor was applied.

Authentication

Describe any authentication procedures for each seed stock used or novel genotype generated. Describe any experiments used to assess the effect of a mutation and, where applicable, how potential secondary effects (e.g. second site T-DNA insertions, mosiacism, off-target gene editing) were examined.

Flow Cytometry

Plots

Confirm that:

☒ The axis labels state the marker and fluorochrome used (e.g. CD4-FITC).

☒ The axis scales are clearly visible. Include numbers along axes only for bottom left plot of group (a 'group' is an analysis of identical markers).

☒ All plots are contour plots with outliers or pseudocolor plots.

☒ A numerical value for number of cells or percentage (with statistics) is provided.

Methodology

Sample preparation	Cell Sorting for single-cell RNA sequencing:<br>Frozen PBMCs were thawed in a 37°C warm water bath until cells were completely thawed. Cells were washed using pre-warmed (37°C) RPMI1640 medium supplemented with 5% of heat-inactivated FBS and centrifuged at 450g for 5 minutes at room temperature. Subsequently, cells were counted using a Neubauer counting chamber. PBMCs were blocked with ChromPure Human IgG to prevent non-specific binding of antibodies. Antibodies against the extracellular antigens CD45 and CD3 in pre-determined dilutions were added and incubated for 30 min at 4°C. Afterwards cells were washed with PBS first followed by two washing steps with annexin V binding buffer. Supernatant was discarded and cell pellet was incubated for 20 min at room temperature in a dark place with annexin V-PE and eFluor 506 viability dye (eBioscience) to avoid sorting of early and late apoptotic and dead cells. After the final centrifugation step cells were resuspended in PBS containing 2mM EDTA to prevent cells from clotting during the process of cell sorting.<br><br>Mass Cytometry barcoding and antibody staining:<br>Samples were barcoded by transiently permeabilizing with saponin and barcoding with a combination of Palladium metal isotopes (102Pd, 104-106Pd, 108Pd, 110Pd), following a 3-out-of-6 scheme, allowing to pool 20 samples. Then, after incubation with anti-human Fc block (Biolegend), pooled barcoded cells were stained with surface antibodies for 30 min at room temperature with gentle agitation (600rpm). For intracellular signaling protein phosphorylation staining, cells were permeabilized with methanol and stained with intracellular antibodies, again for 30 min at room temperature with gentle agitation (600rpm). For intracellular cytokine staining, cells were permeabilized with saponin and stained with intracellular antibodies, for 1 hr at room temperature with gentle agitation (600rpm). Pools of 20 barcoded and antibody-stained samples were analyzed on the mass cytometer instrument (Helios CyTOF, Standard Biotools Inc.), acquiring two sets of pooled samples (40 samples total) per day.
Instrument	BD Aria Fusion cell sorter (BD Biosciences), Helios Mass Cytometer (Standard Biotools Inc)
Software	BD Diva, Mass Cytometer Software (Standard Biotools)
Cell population abundance	Purity of sorted CD3 T cells for single cell RNA-sequencing was determined to be above 90%.
Gating strategy	Cell sorting: CD45+ CD3+ eFluor 506neg Annexin-Vneg T cells were sorted. Mass cytometry: Please see Supplementary Figure 3.

☒ Tick this box to confirm that a figure exemplifying the gating strategy is provided in the Supplementary Information.
