# Supplementary material for: Immunological maladaptation preceding spontaneous preterm birth in human pregnancies
Source: Nat Commun. 2026 Jul 27;17:7121. doi: 10.1038/s41467-026-75605-5 (PMC13408597; doi:10.1038/s41467-026-75605-5)
Supplement: Supplementary file 10 — Transparent Peer Review file [file 41467_2026_75605_MOESM10_ESM.pdf]

nature portfolio

Peer Review File

Immunological Maladaptation Preceding Spontaneous Preterm Birth in Human Pregnancies

Corresponding Author: Professor Petra Arck

This file contains all reviewer reports in order by version, followed by all author rebuttals in order by version.

Version 0:

Reviewer comments:

Reviewer #1

(Remarks to the Author)
Key results:
This study was a comprehensive multi-omics investigation of the maternal peripheral immune response in both term and spontaneous preterm pregnancies. The study presents an incredibly rich dataset including immune cell frequency characterization, immune proteome and transcriptome, with repeated measures study design that also allowed for longitudinal investigation. The authors present that multi-omic perturbations, with specific emphasis on significantly different inter-feature correlations in terms vs preterms, are predictive of preterm birth. The inclusion of treated cells to demonstrate possible differences in immune- and adrenergic-response provided an important mechanistic investigation that strengthened the observational findings.

Validity:
The strength and novelty of this study lie in the study design and richness of data. The authors provide an extensive analysis of the data, including a breakdown of the contributions of each 'ome', identified correlative patterns that distinguish terms from preterms, as well as geneset enrichment analysis to provide a possible biological interpretation for the patterns observed. While the findings suggest potential predictive value for preterm birth, the lack of methodological clarity in some areas (e.g., possible data leakage) limits confidence in the robustness of this conclusion. Greater transparency in the analytical approach would strengthen the authors' claims. Specific recommendations and areas for improved transparency are described below in the Analytical approach section.

Significance:
This study has the potential to make a meaningful contribution to the field of preterm birth research. The multi-omic scope and the thoughtful study design provide a rich dataset that could yield valuable biological insights. While I do not find the current evidence sufficient to support the authors' claim that these signatures are predictive in a clinical sense, the work nonetheless highlights several important avenues for future investigation. In particular, the identification of potential neuroimmunological and stress-related pathways broadens the conceptual framework for understanding preterm birth etiology.
Overall, the study's strength lies in its integrative approach and hypothesis-generating potential, rather than immediate translational predictiveness. With greater methodological transparency and validation, the findings could substantially inform future mechanistic and biomarker studies in this area.

Data and methodology:
The study design and data quality are a strength of this study. However, there were certain areas in which the quality of presentation could have been modified to improve overall clarity and transparency. Specific suggestions are outlined below:
- The link provided for proteomic/mass spectrometry raw data was invalid and I was unable to access this data
- Line 113 it is unclear how participants would have been matched by gestational age, when presumably the gestational age at delivery would be markedly different in the sPTB vs term groups. Whether this refers to gestational age at sampling vs at delivery is unclear.
- The authors report n=24 sPTB participants and n=46 participants, who provided a blood sample at three timepoint during pregnancy. However, the final sample counts as outlines in Fig 1 legend suggests n=14 missing samples in the preterm group and n=20 samples in the term group. Please comment on the exclusion of these samples and whether they may impact the interpretation of these data

- The description of the single-cell RNA-seq subcohort could be clarified. It is stated that the subcohort included women with two consecutive pregnancies to “control for inter- and intra-individual variability,” with n=5 term and n=5 sPTB in each pregnancy. However, it is unclear whether this means that (a) some women experienced two consecutive term or two consecutive preterm births (i.e., recurrent outcomes), or (b) some women had discordant outcomes across pregnancies (e.g., one term and one preterm). Clarifying the structure of these paired samples is important for interpreting how intra-individual variability was assessed and what type of biological variation was controlled for.

Analytical approach:
The manuscript describes performing regression-based feature adjustment and correlation-based filtering prior to the prediction stage. However, it is unclear whether these preprocessing and feature selection steps were conducted within each training fold of the leave-one-out cross-validation or using the full dataset. If the latter, this would constitute data leakage, as information from the held-out sample would have influenced feature selection and model fitting, leading to inflated performance estimates. The authors should clarify whether all feature selection and regression adjustments were nested within the cross-validation procedure. The impact of (even unintended) data leakage can massively over-inflate performance metrics, as reported in the following publication:
Hornaday KK, Werbicki T, Tough SC, Wood SL, Anderson DW, Li CH, Slater DM. Machine learning for the prediction of spontaneous preterm birth using early second and third trimester maternal blood gene expression: A cautionary tale. PLoS One. 2025 Jun 27;20(6):e0310937. doi: 10.1371/journal.pone.0310937. PMID: 40577367; PMCID: PMC12204558.
Improving the clarity around the methods used for model training would strengthen the confidence of the results.
The inclusion of external validation proteomic datasets is a strength of this study, but this was only applicable to models trained on proteomic data alone. There was degradation in model performance in external validation (AUC lower confidence intervals as low as 0.52), though this was not commented on. In addition, while the author’s correctly highlight the homogeneity of this cohort as a potential limitation of this study, a brief discussion of the demographic characteristics of the populations used for external validation would strengthen the comparison and perhaps explain possible discrepancies in the validation. The study would also be strengthened by a comment on the lack of external validation for the multivariable model or other single-omics models.
Reporting only AUC is insufficient to fully evaluate predictive performance. Complementary metrics (e.g., sensitivity/specificity or calibration) and clear details on model validation would strengthen the authors’ claim of predictive value.
The multivariate SG model is presented as the main predictive result, but based on the AUC plots, three of the single-omic models (ICP RF, Freq L and Freq RF) were reported to have better performance (Figure 2e). Clarification is needed regarding how the authors determined the multivariate model to be optimal and whether formal comparisons of model performance were conducted. Additionally, a comment on the predictive value of ICP or cell frequency may be warranted.

Clarity and context:
The authors have sufficiently placed this work within the context of previous work, and the manuscript, while reasonably dense, was clear overall. The interpretation of Figure 3e/f was difficult to follow. The text refers to two groups (cytokine production group and ISO response/proteome group), but it was not clear how these groups were defined or represented in the figure. Providing clearer labeling in the figure and/or a more explicit description of the groups in the legend or main text would help readers understand the comparison being made. As a minor comment, a few of the acronyms used were not defined on their first use (e.g. VDR, RMSE).

Reviewer expertise:
I am familiar with the field of preterm birth and have experience in prediction modeling, so I was able to evaluate the study design, analytical methods, and interpretation of the predictive modeling results. However, I am less familiar with immunological aspects, particularly those related to marker selection and annotation in the single-cell RNA-seq analyses. I am therefore unable to fully assess whether the cell type annotations or immunological interpretations are appropriate.

Reviewer #2

(Remarks to the Author)
This manuscript describes a multi-omics study aimed at predicting spontaneous preterm birth (sPTB). The objective is highly valuable from a clinical perspective, as a reliable predictive test would have significant impact. However, the study in its current form has several fundamental limitations that prevent it from achieving this goal.

Specific Points:

Definition of the Study Phenotype: The manuscript uses the terms PTB and sPTB. It is crucial to explicitly define the study population and consistently use the correct terminology. Spontaneous PTB (driven by labor or premature rupture of membranes) has different underlying mechanisms than medically-indicated PTB. The validity of the conclusions depends on a precise and well-defined cohort.

Concerns Regarding Statistical Power and Analysis Cohort: Although the overall cohort includes 761 pregnancies, the multi-omics data generation was performed on a much smaller subset. The primary analysis for the "omics context" uses only 24 sPTB and 46 TB cases, and the transcriptomics analysis is limited to an even smaller, carefully selected subcohort of 10 vs. 10. This severely limits the statistical power of the study and raises concerns about the reproducibility and reliability

of the discovered signals. The authors should justify this sample selection and acknowledge the high risk of overfitting.

Lack of Robust Validation: A major weakness is the absence of a strong validation step. Relying on public datasets for only a few biomarkers is insufficient. Most critically, the integrated multi-omics model itself was not validated on an independent internal or external dataset. For a study with translational aspirations, this is a critical omission that undermines the potential utility of the proposed biomarkers.

Recommendation: Reject

Reviewer #3

(Remarks to the Author)
In this study the authors evaluate the immunological landscape in the important problem of spontaneous preterm birth. They describe features and cell responses using an impressive multi-omics approach. Some of the strengths of this work is the use of mixed models, the longitudinal matched data, and the specifications of a consistent preterm birth cohort (all spontaneous, no iatrogenic cases).

I have the following minor comments. Please specify how iatrogenic cases were excluded. Lines 110-112 suggest that both medically indicated iatrogenic PTB and risk factors (e.g. cervical insufficiencies) guided the inclusion/exclusion criteria. However line 593 states exclusion as whoever experienced iatrogenic PTB. I find the sentence on results sections can be misleading.

I appreciate the effort detecting low-density granulocytes as this is such a small proportion of cells, and is impressive to detected features associated with this specific population. However, the link to NLR ratio (line 305) should be carefully interpreted, as NLR hospital data works with whole blood, which would include in its majority normal-density granulocytes, while here is a small proportion from the PBMC fraction.

Finally, if possible authors should provide details of cohort description of validation cohorts (line 836), particularly if samples were collected from similar risk population, and if spontaneous/iatrogenic cases were included.

Reviewer #4

(Remarks to the Author)
The goal of this paper was to characterize immune response in maternal blood preceding sPTB, for a better understanding of the pathophysiology and development of predictive biomarkers. This study is extremely well designed and uses rigorous statistical assessments including validation of their predictive test in a holdout dataset. The authors extract significant biological meaning from their multi-omics data by applying sophisticated network approaches to understand underlying biology of PTB. They also identify maternal immune signals that predict PTB weeks/months before delivery, which is a very important contribution to this field. Their study design leverages the existing PRINCE cohort, which had collected longitudinal data throughout pregnancy, which addresses ongoing challenges in this research field. The manuscript is well written and does a great job contextualizing their work within the larger body of maternal-fetal immune signaling in pregnancy. I offer some suggestions to strengthen this paper further.

1. The authors highlight their own work and important literature surrounding the role of stress in the etiology of sPTB in the introduction (Lines 86-90), and have very robust measures of maternal stress in their population (Methods, lines 624-635). In the results, they identify a relationship between maternal stress in the third trimester and sPTB (Line 121). They this potential mechanism at a molecular level in immune cell sub-populations, as they investigate an adrenergic response to a catecholaminergic agonist, which was very innovative. However, I had a hard time following why the stress measures were not further integrated into the multi-omics assessment. It would be interesting to see if these stress markers were correlated with immune and protein analytes in T3 that were predictive of sPTB, or perhaps even statistically mediated these relationships. However, this would add additional analyses to an already highly complex manuscript. The authors could consider dropping the EPDS measures altogether to tighten the focus.

2. The authors consider potential confounders including maternal age, bmi, gravidity/parity, history of PTB and fetal sex. Did the authors have data on the rates of maternal alcohol use or smoking? These could represent additional important confounders since these are also risk factors for PTB and are associated with molecular changes during pregnancy.

3. The authors highlight in the discussion that this study is limited to a single site in western-Europe with a homogenous population. Can the authors also comment on the rate of PTB (5.5%) and especially spontaneous PTB (24/761= 3.1%), as these numbers are much lower than other populations?

4. Line 178-179- I initially interpreted this line that the authors were looking at co-expression of proteins that were statistically different between preterm and term groups, instead of looking at the differences in correlation coefficients between the groups. This is explained more clearly in the methods. Perhaps a slight rewording here could highlight this strength?

5. The authors could consider reporting the overall accuracy (AUC) of their test for predicting PTB in the holdout dataset in

the abstract of the manuscript, as this is a main finding.

6. Can the authors clarify what the X axis is in Fig. 1 B? Is each dot representing a participant?

Version 1:

Reviewer comments:

Reviewer #1

(Remarks to the Author)
I appreciate the author's detailed response and revisions to the comments. No further comments.

Reviewer #2

(Remarks to the Author)
The authors have made substantial efforts to address the concerns raised in the previous review, including improved methodological transparency and additional analyses. The longitudinal design and depth of multi-omics profiling remain important strengths of the study.

However, key concerns remain insufficiently resolved. In particular, the limited sample size, lack of robust independent validation, and continued positioning of the work as a predictive modeling study still constrain the strength of the conclusions.
1. Validation remains insufficient
The external validation relies on a very small cohort (N=3 sPTB cases), which precludes a meaningful assessment of predictive performance. While consistency in effect direction is noted, this does not constitute robust validation, and the continued presentation as “successful validation” is not supported. The authors should reframe this as preliminary evidence and avoid overinterpretation of performance metrics.
2. Study positioning
The manuscript continues to emphasize predictive performance despite limited cohort size and lack of validation. The study would be more appropriately framed as an exploratory longitudinal multi-omics analysis rather than a predictive biomarker model.
3. Sample size and power
The post-hoc power analysis demonstrates detectability above chance but does not address model stability, overfitting, or reproducibility. These limitations should be more explicitly acknowledged.
4. Transcriptomic subcohort
The clarification of the paired design is helpful; however, given the small sample size (10 vs. 10), conclusions should remain at the pathway level and be interpreted as hypothesis-generating.
Minor
The revisions improve clarity and methodological transparency. Minor wording adjustments could further reduce overstatement (e.g., replacing “validated” with “evaluated”).

Reviewer #3

(Remarks to the Author)
Dear Editor and Authors,

Thanks for the opportunity to review the updated manuscript.
Authors have incorporated all feedback, in particular the addition of information about the validation cohorts, strengthening the comparisons findings.
This reviewer has no further concerns with this manuscript.

Kind regards

Reviewer #4

(Remarks to the Author)
The authors revisions to the manuscript have addressed my concerns and have strengthened the manuscript. The addition of supplemental Figure 4 highlights the authors efforts to prevent data leakage and better explain the study design.

Open Access This Peer Review File is licensed under a Creative Commons Attribution 4.0 International License, which permits use, sharing, adaptation, distribution and reproduction in any medium or format, as long as you give appropriate credit to the original author(s) and the source, provide a link to the Creative Commons license, and indicate if changes were made.
In cases where reviewers are anonymous, credit should be given to 'Anonymous Referee' and the source.
The images or other third party material in this Peer Review File are included in the article's Creative Commons license, unless indicated otherwise in a credit line to the material. If material is not included in the article's Creative Commons license and your intended use is not permitted by statutory regulation or exceeds the permitted use, you will need to obtain permission directly from the copyright holder.
To view a copy of this license, visit https://creativecommons.org/licenses/by/4.0/

Point-by-point reply to reviewers' comments on manuscript entitled 'Immunological Maladaptation as a Predictor of Spontaneous Preterm Birth in Human Pregnancies' (NCOMMS-25-78183)

Reviewer #1:

General comment from Reviewer 1 about key results: This study was a comprehensive multi-omics investigation of the maternal peripheral immune response in both term and spontaneous preterm pregnancies. The study presents an incredibly rich data set including immune cell frequency characterization, immune proteome and transcriptome, with repeated measures study design that also allowed for longitudinal investigation. The authors present that multi-omic perturbations, with specific emphasis on significantly different inter-feature correlations in terms vs preterms, are predictive of preterm birth. The inclusion of treated cells to demonstrate possible differences in immune- and adrenergic-response provided an important mechanistic investigation that strengthened the observational findings.

Authors' response:
We appreciate Reviewer 1's positive acknowledgement of our study design and data generation.

General comment from Reviewer 1 about validity: The strength and novelty of this study lie in the study design and richness of data. The authors provide an extensive analysis of the data, including a breakdown of the contributions of each 'ome', identified correlative patterns that distinguish terms from preterms, as well as gene set enrichment analysis to provide a possible biological interpretation for the patterns observed. While the findings suggest potential predictive value for preterm birth, the lack of methodological clarity in some areas (e.g., possible data leakage) limits confidence in the robustness of this conclusion. Greater transparency in the analytical approach would strengthen the authors' claims. Specific recommendations and areas for improved transparency are described below in the Analytical approach section.

Authors' response:
We thank the reviewer for positively acknowledging our analytical approach and concur that thorough consideration is essential for multi-omic modelling, as shown in our study. Below, we address the specific concerns raised about data leakage and methodological transparency.

General comment from Reviewer 1 about significance: This study has the potential to make a meaningful contribution to the field of preterm birth research. The multi-omic scope and the thoughtful study design provide a rich data set that could yield valuable biological insights. While I do not find the current evidence sufficient to support the authors' claim that these signatures are predictive in a clinical sense, the work nonetheless highlights several important avenues for future investigation. In particular, the identification of potential neuro-immunological and stress-related pathways broadens the conceptual framework for understanding the aetiology of preterm birth. Overall, the study's strength lies in its integrative approach and its hypothesis-generating potential, rather than in its immediate translational predictiveness. With greater methodological transparency and validation, the findings could substantially inform future mechanistic and biomarker studies in this area.

Authors' response:
We appreciate the reviewer's evaluation of the value and significance of our study. As outlined in detail below, we have amended our manuscript to include methodological details and validated both multi- and single-omic models using external data sets. We hope that our revised manuscript will inspire mechanistic follow-up and extensive biomarker validation, as suggested.

Reviewer 1's comment #1 regarding data and methodology: The link provided for proteomic/mass spectrometry raw data was invalid and I was unable to access this data.

Authors' response:
We apologize for the inconvenience the reviewer experienced accessing our data sets. We confirm that access to the data is available under this link:
http://datadryad.org/share/LINK_NOT_FOR_PUBLICATION/T5fuYjayHSyji-_fDZpiZ2SRSZGkYnZ7DvviSSIsKj8

Reviewer 1's comment #2 regarding data and methodology: Line 113, it is unclear how participants would have been matched by gestational age, when presumably the gestational age at delivery would be markedly different in the sPTB vs term groups. Whether this refers to gestational age at sampling vs at delivery is unclear.

Authors' response:
We appreciate the comment and have clarified the sample description. The PRINCE study is a prospective longitudinal cohort study with distinctly defined, narrow sampling time points across the three trimesters (trimester 1: week 12-14 of gestation, trimester 2: week 24-26 of gestation, trimester 3: week 34-36 of gestation). Therefore, by design, there was no difference in gestational age at sampling between the term and preterm groups. Furthermore, women with term and preterm birth were matched on maternal age, BMI, parity/gravidity, and fetal sex.

We revised the text in the results section as follows:

This generated a cohort of women with spontaneous PTB (N=24) pregnancies (median gestational age (GA) at birth of 35.7 weeks; Fig. 1a, b, Table 1). This cohort was matched on maternal age, first-trimester body-mass index, gravidity/parity, and fetal sex to a reference group of N=46 participants with TB (median GA at birth: 39.6 weeks). Study visits, including biospecimen collection, occurred serially once each trimester in a narrow gestational age window before the onset of labor (Fig. 1b, Supplementary Fig. 1), resulting in similar GA at sampling across sPTB and TB groups.

Reviewer 1's comment #3 regarding data and methodology: The authors report N=24 sPTB participants and N=46 participants, who provided a blood sample at three timepoint during pregnancy. However, the final sample counts as outlines in Fig 1 legend suggests n=14 missing samples in the preterm group and n=20 samples in the term group. Please comment on the exclusion of these samples and whether they may impact the interpretation of these data.

Authors' response:
We thank the reviewer for providing the opportunity to improve the reporting of the sample cohorts within our study. Fig. 1b from our original submission provided an overview of the samples available for the CyTOF analysis. We have replaced this figure with an overview of the study visits completed by the 70 participants (revised Fig. 1b). The study's longitudinal design occasionally led to missed appointments without participants dropping out of the study. Thus, in some cases, samples were not collected due to a missed study visit (unexpected illness, vacation of the participant). In cases of preterm birth, some participants could not attend the visit scheduled in the third trimester because they had already delivered prematurely. For the TB group (N=46), a total of n=138 samples could have been collected across the three visits, but 135 samples were actually collected. In the sPTB group (N=24), 72 samples were expected in total, with 60 collected. In order to improve clarity, we have summarized the missing data in the table below for the reviewer's reference and updated Figure 1b and the legend in the revised manuscript:

Missed study visits	Term (N=46)	Preterm (N=24)
1st trimester	N=0	N=0
2nd trimester	N=2	N=1
3rd trimester	N=1	N=11

Revised Figure 1b:

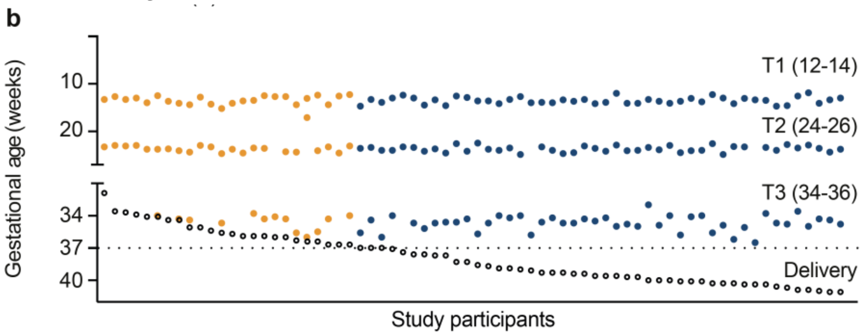

(**b**) Pregnant women completed a study visit at one timepoint in each trimester (T, filled circles) at indicated GAs (weeks) (sPTB, N=24, n=60 samples; TB, N=46, n=135). Open circles indicate GA at delivery. See Supplementary Figure 1 for sample number overviews of PBMC, serum, and single T-cells.

We have also amended the supplement (Supplementary Fig. 1) to include overviews of the specific sample numbers used for CyTOF/immunome analyses in PBMC, Somalogic/proteome analyses in serum, and for scRNAseq analyses in sorted T cells. Sample availability in the PRINCE biobank determined the number of samples available for these analyses.
To improve transparency regarding the sample size used for each analysis, we have added the following graphs to Supplementary Fig. 1:

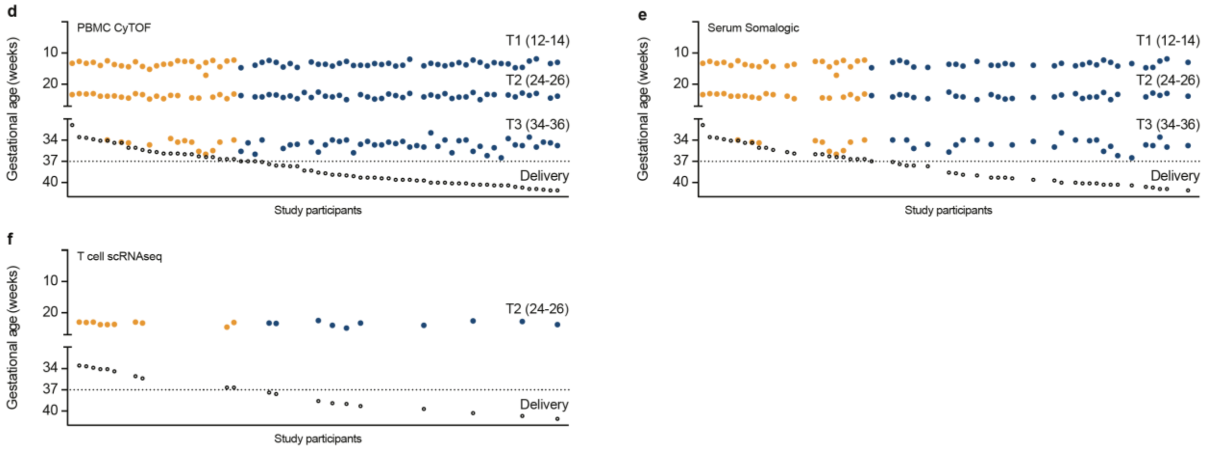

(**d-f**) Sample cohorts of PBMC for CyTOF analyses (d, sPTB: N = 24, n = 60, TB: N = 46, n = 126), serum for aptamer-based proteomics (Somalogic, e, sPTB: N = 21, n = 51, TB: N = 30, n = 84), and T cells for single-cell RNA sequencing (f, sPTB: N = 10, n = 10, TB: N = 10, n = 10). x-axis shows gestational age (GA, weeks) at sampling. y-axis depicts a single patient per row. Participant N and sample n are indicated per group.

Reviewer 1's comment #4 regarding data and methodology: The description of the single-cell RNA-seq subcohort could be clarified. It is stated that the subcohort included women with two consecutive pregnancies to "control for inter- and intra-individual variability", with N=5 term and N=5 sPTB in each pregnancy. However, it is unclear whether this means that (a) some women experienced two consecutive term or two consecutive preterm births (i.e., recurrent outcomes), or (b) some women had discordant outcomes across pregnancies (e.g., one term and one preterm). Clarifying the structure of these paired samples is important for interpreting how intra-individual variability was assessed and what type of biological variation was controlled for.

Authors' response:
To control for inter- and intra-individual variability, we identified this sub-cohort of 10 pregnant women who participated twice in our PRINCE study. These 10 women were grouped as follows:

N	Prior pregnancy	Consecutive pregnancy
2 women	Term	Term
2 women	Preterm	Preterm
3 women	Term	Preterm
3 women	Preterm	Term

We had illustrated this design in Supplementary Fig. 11b with the respective color code (blue for TB and yellow for sPTB).

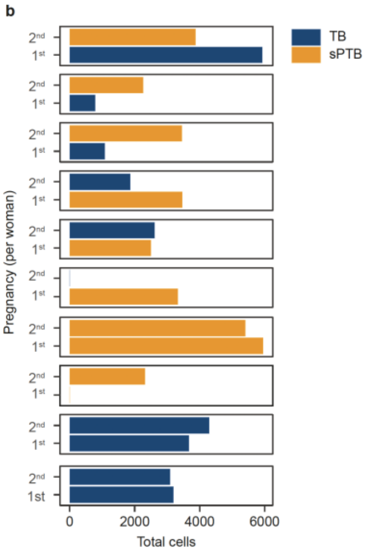

Supplementary Fig. 11: (**b**) Total number of cells of each patient sample (TB = blue, sPTB = yellow).

This cohort design allowed us to control for inter- and intra-patient variability by observing transcriptional immune profiles in consecutive pregnancies. We have now included additional analyses showing that the number of differentially expressed genes is higher when comparing sPTB vs. TB than when comparing transcriptome signatures between a prior and a consecutive pregnancy.

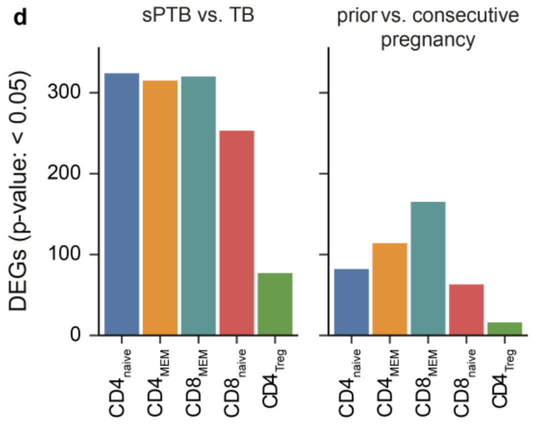

Supplementary Fig. 11: (**d**) The number of differentially expressed genes (DEGs) for each immune cell population when comparing sPTB vs. TB (left) and prior vs. consecutive pregnancy (right).

Thus, gene transcriptional profiles are driven more strongly by PTB status than by birth order, with intra-patient (within-woman) variability across pregnancies being modest relative to inter-patient differences associated with PTB. Accordingly, the inclusion of two pregnancies from the same woman in a unified cohort is justified to maximize power to detect PTB-associated signatures.

We have added these considerations to the results section:

In this context, we evaluated a subcohort of sPTB and TB (N=10 TB, N=10 sPTB), where women with two consecutive pregnancies were analyzed, allowing to control for inter- and

intra-individual variability (Table 2, Methods, Supplementary Fig. 11a–d). Conducting differential gene expression analysis, we found the number of differentially expressed genes was higher when comparing sPTB vs. TB than when comparing the transcriptome signature between a prior vs. a consecutive pregnancy. Thus, gene transcriptional profiles are driven more strongly by sPTB status than by birth order, with intra-patient (within-woman) variability across pregnancies being modest relative to inter-patient differences associated with sPTB. Accordingly, inclusion of two pregnancies from the same woman in a unified cohort was justified to maximize power for detecting sPTB-associated signatures (Supplementary Figure 11d).

Further, we have expanded the methods:

We also analyzed gene expression using a model that specifies delivery order (prior vs. consecutive delivery) to identify genes associated with repeated/multiple pregnancies.

Reviewer 1's comment #5 regarding analytical approach: The manuscript describes performing regression-based feature adjustment and correlation-based filtering prior to the prediction stage. However, it is unclear whether these pre-processing and feature selection steps were conducted within each training fold of the leave-one-out cross-validation or using the full data set. If the latter, this would constitute data leakage, as information from the held-out sample would have influenced feature selection and model fitting, leading to inflated performance estimates. The authors should clarify whether all feature selection and regression adjustments were nested within the cross-validation procedure. The impact of (even unintended) data leakage can massively over-inflate performance metrics, as reported in the following publication:
Hornaday KK, Werbicki T, Tough SC, Wood SL, Anderson DW, Li CH, Slater DM. Machine learning for the prediction of spontaneous preterm birth using early second and third trimester maternal blood gene expression: A cautionary tale. PLoS One. 2025 Jun 27;20(6):e0310937. doi: 10.1371/journal.pone.0310937. PMID: 40577367; PMCID: PMC12204558.
Improving the clarity around the methods used for model training would strengthen the confidence of the results.

Authors' response:
We appreciate Reviewer 1's concern about data leakage. We would like to clarify that no correlation-based filtering or regression-based feature adjustment was performed prior to partitioning the data. Aware of this potential for overfitting, we designed the pipeline from the outset to prevent data leakage to the ensemble layer. Importantly, all processing of raw data occurs within each iteration of the inner-LOOCV loop, including tuning of hyperparameters, which occurs within additional splits of the training data within the inner-LOOCV loop. We inserted an extended figure (new Fig. S4b) that shows the data-splitting scheme, as well as the Snakemake pipeline we used to train the models according to pre-specified data splits:

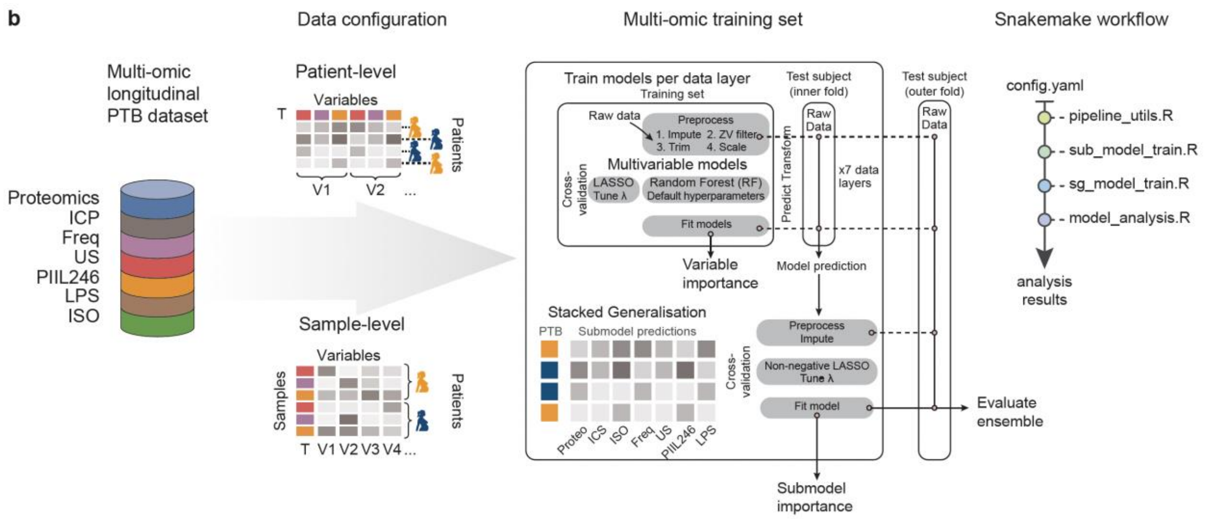

Supplementary Fig. 4: (b) Schematic of a multi-omic modeling approach to reveal gestational immune and proteomic trajectories and to identify potential biomarker candidates. The model is built using a patient-level (Fig. 2) or sample-level (Fig. 5) data configuration for distinguishing sPTB from TB (Methods). T: trimester, V: variable

In the revised manuscript, we now describe the cross-validation details for both the outer and inner folds in the methods, and we have clarified additional details:

Leave-one-patient-out cross-validation and data preprocessing

We employed a nested leave-one-patient-out cross-validation (LOOCV) approach in all modeling to prevent overfitting and to obtain an unbiased estimate of model performance on unseen samples. The overall cross-validation design comprised three nested levels: (i) an outer LOOCV loop used to evaluate the stacked generalization ensemble; (ii) an inner LOOCV loop within the outer training set used to train and generate out-of-sample predictions from each submodel; and (iii) a grouped V-fold cross-validation within each inner training iteration used for hyperparameter tuning. Within each training set, data preprocessing was applied per feature in the following order: imputation of missing values using the median; winsorization at a 0.05 quantile threshold; exclusion of zero-variance features; centering by subtracting the mean; and scaling by dividing by the standard deviation. Preprocessing parameters estimated from the training set were then applied to the corresponding test set. For classification analyses, a weighted loss function was used during model training to address class imbalance.

Multivariable analyses and stacked generalization

Model training followed the nested LOOCV structure described above. For each omic data set, submodels were trained within the inner LOOCV loop using the least absolute shrinkage and selection operator (Lasso 72) and Random Forest (RF 73) using the Tidymodels 74 machine learning framework. For classification analyses, Lasso was implemented as penalized logistic regression with L1 regularization; for regression analyses (predicting gestational age at birth), penalized linear regression with L1 regularization was used. The penalty parameter λ was tuned via 5-times repeated, 20-fold grouped cross-validation, where folds were constrained such that all samples from a given patient remained within the same fold to prevent data leakage. Folds were stratified by the response variable in classification analyses. A grid of 100 candidate penalty values was evaluated, selecting the value that optimized the area under the receiver operating characteristic curve (AUROC) for classification or root mean squared error (RMSE) for regression. Random Forest classifiers were trained with 1,000 trees using the ranger engine with impurity-based variable importance. The top 25 features ranked by importance were used to train the final RF submodel, following the approach established in the DREAM preterm birth challenge 20.

To integrate predictive signals across omics layers, we used stacked generalization (SG 75) in the outer LOOCV loop. In each outer iteration, submodels were trained on the training data using the inner LOOCV loop. Their out-of-sample predictions were then used as inputs for a second-layer Lasso model constrained to non-negative coefficients, yielding a weighted

combination of submodels. The SG layer processed submodel predictions with mean imputation. This nested cross-validation design ensured that SG performance estimates were unbiased and avoided information leakage between submodels and the ensemble layer.

We have also added details of the Snakemake workflow to the methods:

We designed a Snakemake pipeline to facilitate training and evaluating the individual models across configurations. The pipeline comprised three sequential rules: (1) training of submodels across all omic layers in parallel; (2) training of the stacked generalization ensemble using submodel predictions; and (3) model evaluation and analysis. To enable parallel execution, all outer cross-validation splits were predefined as omic layer–patient pairs, allowing independent model training iterations to be distributed across available cores.

Reviewer 1's comment #6 regarding analytical approach: The inclusion of external validation proteomic data sets is a strength of this study, but this was only applicable to models trained on proteomic data alone. There was degradation in model performance in external validation (AUC lower confidence intervals as low as 0.52), though this was not commented on. In addition, while the authors correctly highlight the homogeneity of this cohort as a potential limitation of this study, a brief discussion of the demographic characteristics of the populations used for external validation would strengthen the comparison and perhaps explain possible discrepancies in the validation. The study would also be strengthened by a comment on the lack of external validation for the multivariable model or other single-omics models.

Authors' response:
We appreciate the opportunity to elaborate on the demographic characteristics of the validation cohorts in the revised manuscript.

We added the following summary to the results:

In brief, the validation cohorts differ significantly in geographical range (six sites in Asia, Africa, and North America) and race (Asian, African, African American) from the test cohort, which is Middle European and Caucasian. These discrepancies might explain the lower model performance in these data sets. Despite these differences, β–NGF, IL-20Ra, and CD70 are predictive across all data sets, suggesting generalizability across demographic domains, which will require additional validation in future studies.

We further amended the method section by providing detailed cohort descriptions:

The plasma samples in the <u>Jama study</u> were derived from five biorepository-supported cohorts in Matlab, Bangladesh; Lusaka, Zambia; Sylhet, Bangladesh; Karachi, Pakistan; and Pemba, Tanzania. A total of 81 pregnant women were included, of which 39 had PTBs (48.1%) and 42 had term pregnancies (51.9%). Medically-indicated preterm deliveries were excluded. The mean (SD) maternal age was 24.8 (5.3) years. The median GA at sampling was 13.6 weeks.

Plasma samples in the <u>SciAdv study</u> were derived from five biorepository-supported cohorts in Matlab, Bangladesh; Lusaka, Zambia; Sylhet, Bangladesh; Karachi, Pakistan; and Pemba, Tanzania. This cohort was entirely separate from the Jama cohort (ref). 113 pregnant women with a PTB (48.9%) and 118 with a term delivery (51.1%) were included, of which N=224 (97.8%) occurred spontaneously. Median (IQR) maternal age was 25 (22-29). Samples were collected before gestational week 20.

Samples in the <u>Dream cohort</u> were derived from the Perinatology Research Branch, Wayne State University, and the Detroit Medical Center in the US. […] A total of n=210 plasma samples (2 samples per participant) were available from N=39 controls, N=62 sPTB, and N=4 PPROM cases. Mean maternal age (IQR) was 25 (22-27), 24 (21-28), and 24 (21.8-26.2), and

African American race 87.2%, 95.2%, and 75%, respectively. Blood samples were collected at the time of prenatal visits, scheduled at 17-22, and at 27-33 weeks' gestation.

Reviewer 1 had further pointed to the lack of external validation of the multi-variable model or other single-omics models. We now report robust performance of the multi-variable model in an external validation data set (please also see our more elaborate reply to Reviewer 2). Specifically, applying our multi-omic model to a previously published set of longitudinal, integrated immunome and proteome data successfully classified term from preterm pregnancies (AUC = 0.92).

This analysis has been added in extended Supplementary Fig. 4e-g, and is explained in results and methods accordingly:

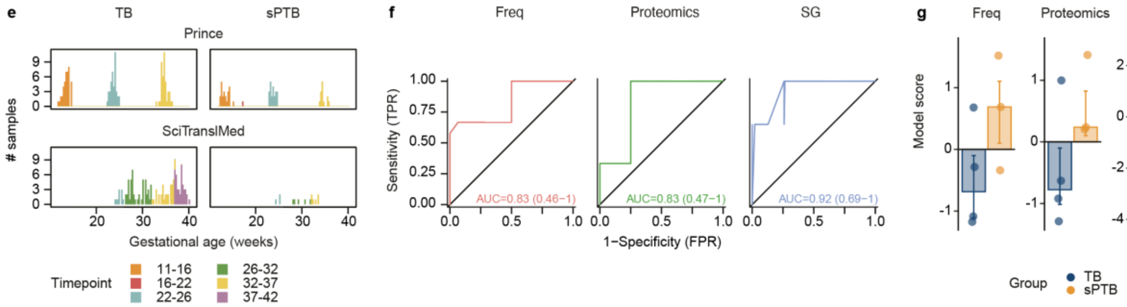

Supplementary Fig. 4: (**e**) Overview of blood samples and measurements in the test (Prince) vs. validation (SciTranslMed) data sets. Histograms depicting the number of TB and sPTB samples with mass cytometry data and proteomics over the course of gestation. Bars are colored according to time periods corresponding to Prince sample timepoints T1, T2, and T3. (**f**) Results of the multi-omic model predicting sPTB in sPTB with mass cytometry (frequencies) and proteomics data. ROC curve and AUC. (**g**) Prediction scores, median (IQR) for each participant in TB vs. sPTB groups.

Results:
Finally, we successfully validated this model in an external, publicly available data set of whole blood samples serially collected from sPTB and TB pregnancies [19], reaching an AUC of 0.92 in the integrated model (Supplementary Fig. 4e-g, Supplementary Table 4,5). While the sample size limits formal statistical power, the magnitude, direction and consistency of the effect sizes observed in the model scores on the test set provide supportive evidence for the robustness and external validity of our multi-omic model.

Methods:
Validation of multi-omic features
To assess the generalizability of the multiomic model, we leveraged an independently generated longitudinal multiomic cohort from a previous study that collected integrated mass cytometry and aptamer-based proteomic data during pregnancy ("SciTranslMed" study) [19]. Gestational age at sample collection was aligned between the two studies by binning into matching time intervals, restricting the analysis to T2 and T3 timepoints. When multiple samples from the same patient fell within a single time interval, the earliest sample was retained. Only patients with samples at both T2 and T3 were included, yielding a test set of 6 sPTB samples (N=3) and 8 term samples (N=4). The analysis was restricted to overlapping features, i.e., the same immune cell frequency features and proteins measured by both the training and test cohort platforms (matched by UniProt identifiers) (Supplementary Table 5,6).

To account for batch effects between cohorts, features in the SciTranslMed data set were standardized (z-scored) within the cohort rather than applying the training cohort's preprocessing parameters. This approach removes cohort-level location and scale effects while preserving within-cohort relative differences, enabling evaluation of whether the learned model weights maintain discriminative ability in an independent data set. For the frequency and proteomic submodels, model scores were computed as the linear combination of standardized feature values weighted by the corresponding Lasso coefficients from the training

study. Submodel scores were then standardized and combined using the stacked generalization weights to produce a final ensemble score. Model discrimination in the cohort was evaluated using the area under the ROC curve (AUROC) with 95% confidence intervals, and group differences were assessed with the Wilcoxon rank-sum test.

To additionally validate the single-omic immunome/CyTOF model, we leveraged second-trimester blood transcriptome data from pregnancies that developed sPTB measured in the publicly available DREAM study [20]. The study performed bulk whole-blood RNA sequencing in the second and third trimesters in sPTB vs. sTB pregnancies. The sampling timepoints in the Dream study overlapped with those in our cohort.
Using blood transcription modules [92], we identified patterns that reflect signatures we report in our CyTOF/immunome data set (Fig. 3, amended Supplementary Fig. 9b,c). Specifically, granulocyte, T cell activation, and monocyte inflammation modules confirm our cytometric findings of increasing monocyte, granulocyte, and CD8 effector frequencies.

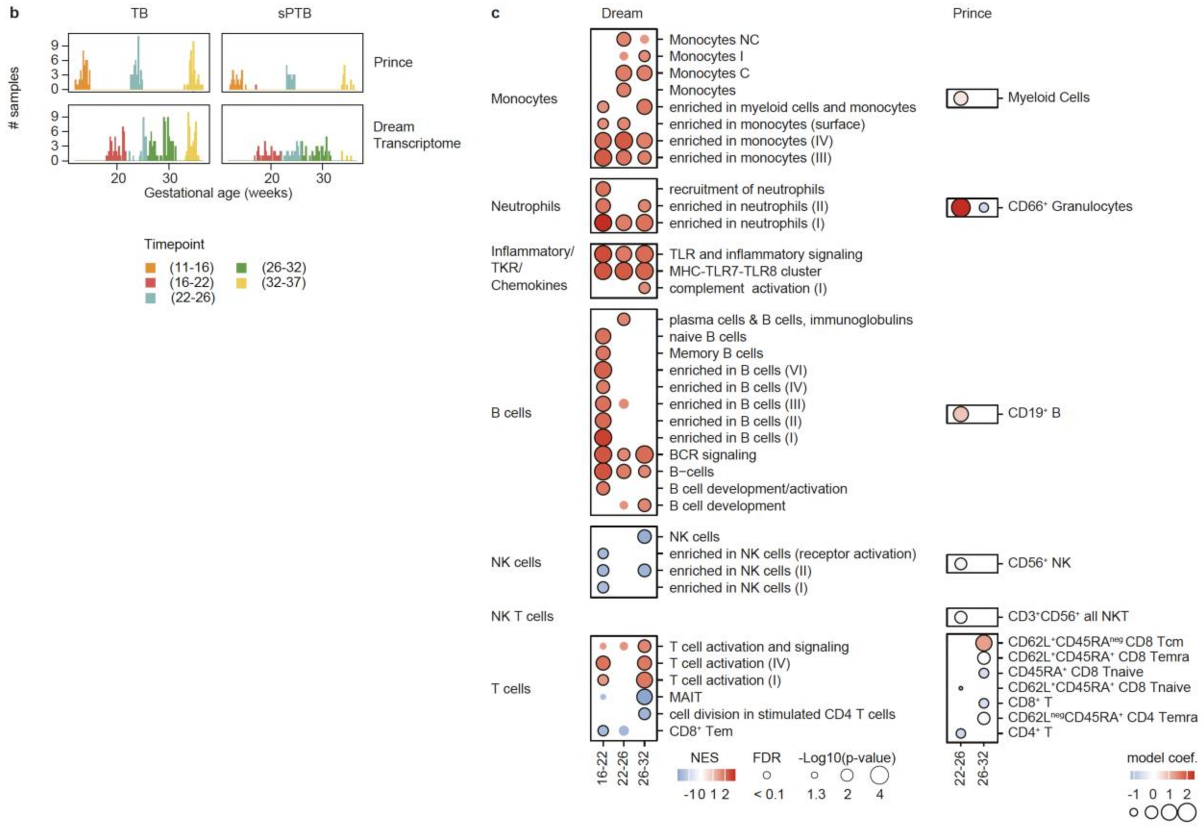

Supplementary Fig. 9: (**b**) Blood transcription modules derived from whole blood microarray re-analysis (Dream study) recapitulate top immune features predictive of sPTB, identified using single-cell proteomics in this study (Prince). (**c**) Left: Bubble plot of gene set enrichment analyses (left y-axis) in sPTB vs. term pregnancies within transcription modules corresponding to immune cell populations. On the right y-axis, blood transcription modules are shown for different immune cell populations, each bubble corresponds to a normalised enrichment score (NES) for a module at a timepoint on the x-axis. Right: Bubble plot of the PRINCE model coefficients in the frequency submodel, i.e., each frequency feature's contribution to the prediction model.

We amend the result section as follows:

To externally validate the findings within the single-immunome submodels, we identified a study among publicly available data sets of sPTB prediction that measured the bulk whole-blood transcriptome in the second and third trimester explicitly in sPTB vs. sTB ("Dream" study) [20]. This study analyzed blood at timepoints that overlapped with our study (Supplementary Figure 9b). Using blood transcription modules (Methods), we identified patterns that reflect signatures we report in the top-predictive Freq and ICP single-omic models (Fig. 3g, m, Supplementary Figure 9c). Specifically, granulocyte, T-cell activation and monocyte

inflammation modules confirmed our cytometric findings of increased monocyte, granulocyte, and CD8 effector frequencies and/or cytokine production.

We further updated the methods section:

For microarray validation analyses, differential expression was performed using limma, with duplicateCorrelation to account for repeated samples from the same individual. Genes were ranked by log fold change from the limma model for GSEA input. For microarray analyses, blood transcription modules [92] and the NeuroImmune set above were analyzed by GSEA.

Reviewer 1's comment #7 regarding analytical approach: Reporting only AUC is insufficient to fully evaluate predictive performance. Complementary metrics (e.g., sensitivity/specificity or calibration) and clear details on model validation would strengthen the authors' claim of predictive value.

Reviewer 1's comment #8 regarding analytical approach: The multivariate SG model is presented as the main predictive result, but based on the AUC plots, three of the single-omic models (ICP RF, Freq L and Freq RF) were reported to have better performance (Figure 2e). Clarification is needed regarding how the authors determined the multivariate model to be optimal and whether formal comparisons of model performance were conducted. Additionally, a comment on the predictive value of ICP or cell frequency may be warranted.

Authors' response:
We are combining our response to comments #7 and #8, as both address analytical approaches. We have addressed the comments regarding additional metrics, model comparison, and the selection of the multivariate model as follows:

Performance metrics: We now provide a summary table (Supplementary Table 3) including Sensitivity and Specificity based on the optimal Youden's J threshold for each model. To address the concern about "predictive value, we now also include newly calculated calibration curves for the SG model and submodels with higher nominal AUROC (revised, extended Supplementary Fig. 4c). Our calibration analysis reveals that ICP (RF) is indeed well-calibrated, whereas the SG (Lasso) model displays a slight tendency toward overconfidence. We have added the following to Supplementary figure 4:

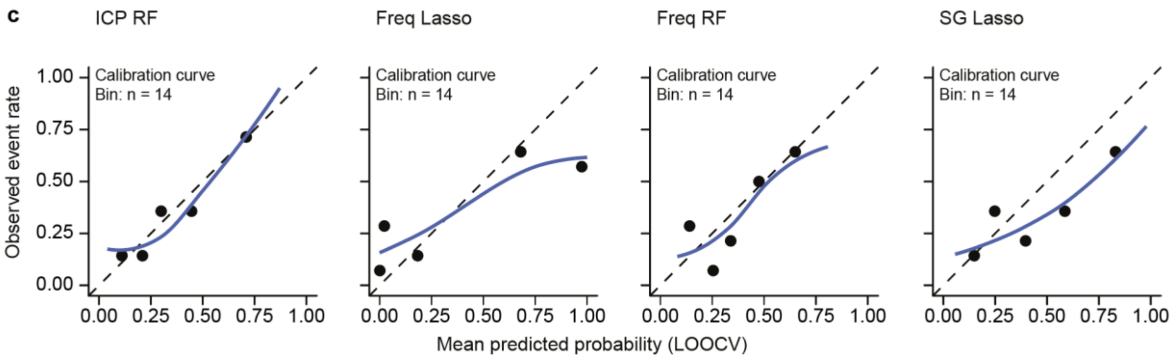

Supplementary Fig. 4: (**c**) Calibration curves of the top predictive single-omic models and the multi-omic SG model to assess how well predicted risk corresponds to the observed outcomes. Circles on the diagonal indicate a well-calibrated model; circles above or below indicate under- or overconfidence in the estimated risk, respectively.

Statistical comparison of models by Delong Test: we performed formal statistical comparisons using the DeLong test. While ICP (RF) achieved a nominally higher AUROC (0.75) compared to the multivariate SG (Lasso) (0.70), this difference was not statistically significant ($p = 0.363$). In fact, no single-omic model significantly outperformed the multiomic model (all $p > 0.72$).

Selection of the multi-omic SG model: Unlike the single-omic models, the SG (Lasso) model captures cross-comic interactions and provides a systems-level view of sPTB, which in our view is more biologically relevant. In addition, the SG (Lasso) model demonstrated the highest specificity (84.8%) among all tested models, making it the most robust choice for minimizing false positives.

Additional details on model validation also include that the ensemble layer and individual submodels were protected from data leakage (discussed in response to comment #5) and that the multi-omic model weights were externally validated in a test cohort (discussed above and in our response to Reviewer 2's comment on external validation of the multi-omic model).

We have amended the methods and results sections of our revised manuscript to address the comments #7 and #8 raised by Reviewer 1 as follows:

Results:
While the model built from cytokine production (ICP) alone reached a nominally higher AUROC (0.75) compared to the multivariate SG (Lasso) (0.7), these differences were not statistically significant ($p = 0.36$). In fact, no single-omic model significantly outperformed the multi-omic model (all $p > 0.72$) (Supplementary Table 3). The SG (Lasso) model demonstrated the highest specificity (84.8%) among all tested models, making it the most robust choice for minimizing false positives, while capturing cross-comic interactions and providing a systems-level view of sPTB. We also assessed how well the predicted risk corresponded to the observed outcomes for the top four models using calibration curves, revealing that ICP (RF) is indeed well-calibrated, whereas the SG (Lasso) model displays a slight tendency toward overconfidence (Supplementary Fig. 4c).

Methods:
<u>Model comparison and calibration</u>
To compare predictive performance across models, we computed the area under the receiver operating characteristic curve (AUROC) with 95% confidence intervals, along with sensitivity and specificity at the optimal classification threshold determined by Youden's J index. Formal pairwise comparisons between the stacked generalization model and individual submodels were performed using the DeLong test for correlated ROC curves. Model calibration was assessed using reliability diagrams, in which predicted probabilities were grouped into five quantile-based bins and plotted against observed event rates, with a LOESS calibration curve fitted to the unbinned predictions.

Lastly, the predictive value of ICP and cell frequency will be useful once translating features identified here into clinical applications, i.e., an sPTB risk test. Clinical labs can readily determine Complete Blood Counts including frequencies. Cytokine production assays, while currently not universally available in clinical labs, are attainable to establish in dedicated facilities, e.g., Quest Diagnostics and LabCorp.

We have added this notion of the potential for future clinical usefulness of our predictive analytes to the discussion:

The predictive value of cytokine production capacity, cell frequency, and adrenergic response identified here as predictive of sPTB will become apparent once translating these features into clinical applications, i.e., a sPTB risk test. Clinical labs can readily determine Complete Blood Counts including frequencies. Cytokine production and adrenergic response assays, while currently not universally available in clinical labs, are attainable to establish in dedicated facilities.

Reviewer 1's comment #9 regarding clarity and context: The authors have sufficiently placed this work within the context of previous work, and the manuscript, while reasonably dense, was clear overall. The interpretation of Figure 3e/f was difficult to follow. The text refers

to two groups (cytokine production group and ISO response/proteome group), but it was not clear how these groups were defined or represented in the figure. Providing clearer labelling in the figure and/or a more explicit description of the groups in the legend or main text would help readers understand the comparison being made.

Authors' response:
This comment helped us streamline the figure and its description. We have included cluster labels for the two groups in Fig. 3e, and have edited the text, now reading:

To understand the relationships between the set of informative features, a correlation network was calculated that qualitatively segregated the features into two groups (Fig. 3e): in the group on the lower right, cytokine production (ICP) features clustered with frequency features of naïve T cells, and in the group on the upper left, ISO response and proteome features grouped myeloid and T cell responses with neuroactive proteins (Fig. 3e, f).

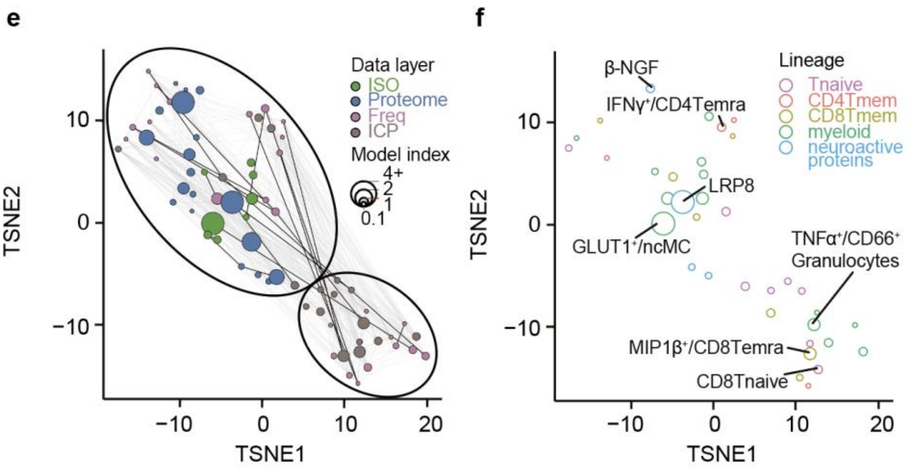

Fig. 3: (**e**) … Circles denote manually assigned clusters. (f) Immune cell lineage and protein mask overlaying …, highlighting selected …

Reviewer 1's comment #10 regarding clarity and context: As a minor comment, a few of the acronyms used were not defined on their first use (e.g. VDR, RMSE).

Authors' response:
We have checked the manuscript according to the reviewer's comment and now introduce all abbreviations.

Reviewer #2:

General comment from Reviewer 2 about key results: This manuscript describes a multi-omics study aimed at predicting spontaneous preterm birth (sPTB). The objective is highly valuable from a clinical perspective, as a reliable predictive test would have significant impact. However, the study in its current form has several fundamental limitations that prevent it from achieving this goal.

Authors' response:
We appreciate Reviewer 2's spotlight on the shortcomings of our manuscript. We have addressed these concerns in our responses below.

General comment from Reviewer 2 about definition of the study phenotype: The manuscript uses the terms PTB and sPTB. It is crucial to explicitly define the study population and consistently use the correct terminology. Spontaneous PTB (driven by labor or premature rupture of membranes) has different underlying mechanisms than medically-indicated PTB. The validity of the conclusions depends on a precise and well-defined cohort.

Authors' response:
Our cohort exclusively includes spontaneous preterm birth (sPTB) cases, as described in the methods and first results paragraph. We thank Reviewer 2 for highlighting these inaccuracies and have ensured that sPTB is now used consistently throughout the revised version of our manuscript.

General comment from Reviewer 2 about concerns regarding statistical power and analysis cohort: Although the overall cohort includes 761 pregnancies, the multi-omics data generation was performed on a much smaller subset. The primary analysis for the omics context uses only 24 sPTB and 46 TB cases, and the transcriptomics analysis is limited to an even smaller, carefully selected subcohort of 10 vs. 10. This severely limits the statistical power of the study and raises concerns about the reproducibility and reliability of the discovered signals. The authors should justify this sample selection and acknowledge the high risk of overfitting.

Authors' response:
We appreciate Reviewer 2's concern about sample selection and limitations regarding the statistical power. We would like to highlight that the PRINCE study is a population-based, prospective, longitudinal (repeat sampling) cohort of women who became pregnant spontaneously. During the study visits, a trained obstetrician examined these women in each trimester. As a result, the medical data is both highly detailed and of excellent quality. Based on the overall study design, the PRINCE participants can be considered a low-risk population for pregnancy complications. Among the 761 women participating in the total study, 40 women gave birth before 37 completed weeks of gestation and were defined as PTB (5.5%). Among these total PTB cases, an onset of labor resulting in delivery occurred in 24 women, whereby onset of labor was defined as contractions and preterm premature rupture of membranes (PPROM). These cases were classified as spontaneous PTB (60% sPTB of all PTB cases) and used for the analysis presented in our current study. Medically-indicated, iatrogenic cases of PTB, e.g., induced delivery before gestation week 37 due to preeclampsia, fetal distress or growth restriction, vaginal bleeding, or uterine infections (N=16) were not included in the analyses of the present study. We concur with Reviewer 2 that having N=24 women with sPTB might seem limited. However, based on the prospective study design and repeated longitudinal sampling of blood and clinical data before the onset of sPTB, we consider our data set highly distinctive and suitable for identifying immunopathology pathways preceding sPTB onset in a low-risk population.

To address Reviewer 2's main concern about whether a significant discriminative effect can be detected with our available sample sizes, we conducted a post-hoc power analysis using

the Obuchowski et al. method, as implemented in the pROC R package [29,30]. This framework evaluates whether a given sample size is sufficient to distinguish a true AUC from chance (0.5). At our sample sizes and $\alpha = 0.05$ (two-sided), the study is powered at 80% to detect an AUC of 0.697, consistent with our observed LOOCV AUC of 0.70.

We amended the results as follows:

Lastly, we performed a post-hoc power analysis using the method of Obuchowski et al. as implemented in the pROC R package [29,30]. With 24 cases and 46 controls, our study had 80% power ($\alpha = 0.05$, two-sided) to detect an AUC of 0.697 as significantly different from chance (AUC = 0.5). Our observed leave-one-out cross-validated AUC of 0.70 corresponds to a power of 81.2%, exceeding the conventional 80% threshold.

Further, Reviewer 2 pointed out the even lower sample size of the subcohort used for transcriptomic analysis (10 vs. 10 pregnancies). We acknowledge that this sample size limits the power to detect individual gene-level effects. However, this subcohort is a unique opportunity as it includes 10 women who participated with two consecutive pregnancies, allowing us to utilize this unique setting to control for inter- and intra-individual variability in T cell profiles across consecutive pregnancies. Notably, our analyses showed that there were more differentially expressed genes when comparing sPTB to TB than when comparing a prior pregnancy to a subsequent one (see response to Reviewer 1 #4 above and new Supplementary Fig. 11d), supporting the observed sPTB signal rather than confounding by temporal or individual variation.

In order to describe the subcohort used for transcriptomic analysis more comprehensively, we amended the method section as follows:

Cohort description
Within our selective cohort, we identified 10 women who participated in the PRINCE study with two consecutive pregnancies. Of these participants, two women experienced two consecutive TB and two women experienced two sPTB. The remaining six women had one TB and one sPTB. Of these six, three women first had a TB and a subsequent sPTB, while the other three women first had a sPTB and then a TB pregnancy (Supplementary Fig. 11b).

We wish to clarify that this transcriptomic analysis was designed as an exploratory investigation to identify candidate biological pathways, not as a definitive gene-level discovery effort. Our primary biological interpretation was based on gene set enrichment analysis (GSEA), which leverages the full ranked gene list to detect coordinated pathway-level changes rather than relying on individually significant genes. Importantly, we externally validated our GSEA findings in T-cells using an independent data set of whole blood transcriptomic profiles (Dream study) [20], which demonstrated convergent pathway-level results when enriching for our custom neuroimmune gene set. We had found that a set of these neuroimmune genes were significantly enriched in sPTB vs. TB (Fig. 4d,e). In accordance, in the Dream study, these genes were similarly positively enriched (increased expression, trending towards significance) at two timepoints in the second trimester (GA weeks 16-22 and 22-26) in sPTB cases (new Supplementary Fig. 15).

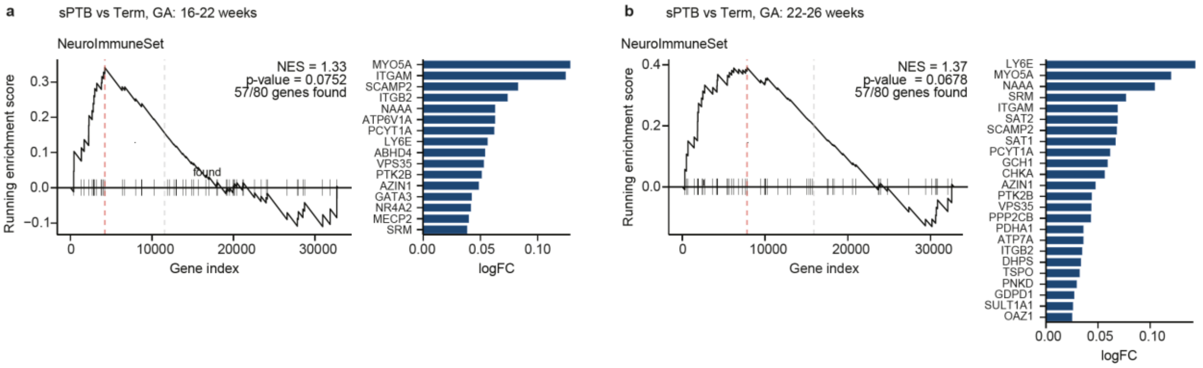

Supplementary Fig. 15: External validation of neuroimmune transcriptome signatures in sPTB vs. TB. Whole blood microarray (bulk transcriptome) re-analysis (Dream study) trends towards recapitulating single-T cell transcriptional profiles of neuroimmune gene set expression in second trimester sPTB vs. TB. (a) Gestational week 16-22 and (b) 22-26. Left: Gene set enrichment plots for the neuroimmune gene set. NES, normalized enrichment score. The nominal p-value of the enrichment is shown, together with the total number of genes in the pathway with detectable expression in bulk transcriptome (genes found). Right panel: Log foldchange (logFC) of leading-edge genes differentially expressed in sPTB vs. TB.

We have updated the results to more clearly frame the transcriptomic analysis as exploratory:

This analysis was designed as an exploratory investigation to identify candidate biological pathways, not as a definitive gene-level discovery effort.

Further, the analysis shown above has been added as new Supplementary Fig. 15:

Results:
We externally validated these single-cell transcriptomic findings, testing for enrichment of our custom neuroimmune gene set, used in the scRNAseq analysis in Fig. 4d,e, at each timepoint in the “Dream study, a whole-blood bulk transcriptomic data set of sPTB vs. TB cases [20]. Demonstrating convergent pathway-level results (Fig. 3c, 4c, d), neuroimmune-related genes were positively enriched (increased expression, trending towards significance) in the second trimester (GA weeks 16-22 and 22-26) in sPTB vs. TB cases (Supplementary Fig. 15). Congruent with our finding of a lack of ISO feature contribution to sPTB risk prediction in late pregnancy, this association was lost in the third trimester (GA weeks 26-37) (not shown).

Methods:
For microarray validation analyses, differential expression was performed using limma, with duplicateCorrelation to account for repeated samples from the same individual. Genes were ranked by log fold change from the limma model for GSEA input. For microarray analyses, blood transcription modules [92] and the NeuroImmune set above were analyzed by GSEA.

Lastly, in the revised discussion, we acknowledge that sample sizes in discovery and validation cohorts generally require generalizability testing:

While this resulted in a rather homogenous population with limited number of patients, further studies are essential to ascertain the generalizability of biomarker candidates and neuroimmunoendocrine mechanisms implicated in the pathobiology of sPTB in other populations. Large international, multi-center studies with sizable, yet well-characterized patient groups are required to establish reproducibility and reliability of the discovered signals and to make relevant steps towards improved clinical management.

Noteworthy, we expand on the incidence of PTB cases of our study also in response to Reviewer 4. In brief, the PTB rate in the PRINCE study corresponds to the general rate in Hamburg (5.9%), which is lower than the national average (8.3%). Hamburg, one of Germany’s wealthiest and largest cities, has a population with higher socioeconomic status, such as higher average income, better access to education, and robust social support systems relative

to other German states. These factors are linked to improved nutrition, higher physical activity, earlier prenatal care, lower smoking rates during pregnancy, and fewer untreated infections, which could explain Hamburg's low PTB rate. We added this information in the results section as follows:

Within this study, a total of N=40 women experienced PTB (5.5%). While this corresponds to the general PTB rate in the city state of Hamburg (5.9%), it is lower than the national average in Germany (8.3%) and might be explained by Hamburg’s general high socioeconomic status relative to other German states.

General comment from Reviewer 2 about lack of robust validation: A major weakness is the absence of a strong validation step. Relying on public data sets for only a few biomarkers is insufficient. Most critically, the integrated multi-omics model itself was not validated on an independent internal or external data set. For a study with translational aspirations, this is a critical omission that undermines the potential utility of the proposed biomarkers.

Authors’ response:
We agree with Reviewer 2 that external validation is critical for establishing the generalizability of biomarker discovery efforts. Yet, our multi-omic data sets, which enable validation of single-cell proteomic (e.g., mass or flow cytometry-based) and plasma proteomic predictors of spontaneous preterm birth, are rare and thus unique. To our knowledge, only one prior longitudinal study in pregnancy generated integrated mass cytometry and proteomic profiling data sets: our previous study aimed to predict time to spontaneous onset of labor using a cohort of mainly term birth pregnancies (N>50) to demonstrate proof-of-concept, with a small N=5 of sPTB cases [19].
We used this independently generated multi-omic cohort as a test data set to rigorously evaluate the performance and generalizability of our model. Aligning GA at sample collection between the two studies restricted the analysis to a test set of n = 6 sPTB samples (N=3 sPTB) and n = 8 term samples (N=4 sTB) for whom longitudinal blood samples were collected at the T2 and T3 timepoints.
We standardized features within the test cohort to remove cohort-level location/scale effects and evaluated whether the learned linear combination in our current study preserves within-cohort discrimination. We restricted the analysis to the same proteins and immune cells measured in our training study (new Supplementary Tables 5,6). This analysis showed discrimination within this test data set, i.e., ranking sPTB vs sTB (AUCs: 0.83-0.92 in frequency, proteomics, and the stacked generalization/sg). The magnitude, direction and consistency of the effect sizes observed in the model scores on the test set provide supportive evidence for the robustness and external validity of our multi-omic model.

This analysis has been added in extended Supplementary Fig. 4e-g, and is explained in results and methods as follows:

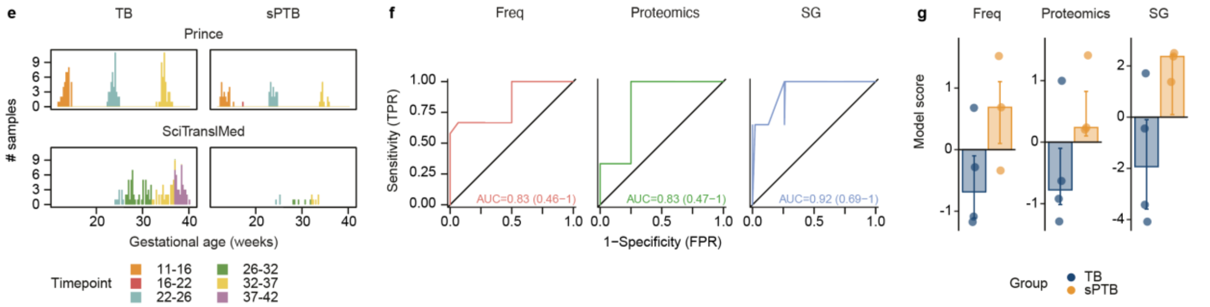

Supplementary Fig. 4: (**e**) Overview of blood samples and measurements in the test (Prince) vs. validation (SciTranslMed) data sets. Histograms depicting the number of TB and sPTB samples with mass cytometry data and proteomics over the course of gestation. Bars are colored according to time periods corresponding to Prince sample timepoints T1, T2, and T3. (**f**) Results of the multi-omic model predicting sPTB in sPTB with mass cytometry (frequencies) and proteomics data. ROC curve and AUC. (**g**) Prediction scores, median (IQR) for each participant in TB vs. sPTB groups, Wilcoxon test p-value.

Results:

Finally, we successfully validated this model in an external, publicly available data set of whole blood samples serially collected from sPTB and TB pregnancies [19], reaching an AUC of 0.92 in the integrated model (Supplementary Fig. 4e-g, Supplementary Table 4,5, Methods). While the sample size limits formal statistical power, the magnitude, direction and consistency of the effect sizes observed in the model scores on the test set provide supportive evidence for the robustness and external validity of our multi-omic model.

Methods:

Validation of multi-omic features

To assess the generalizability of the multiomic model, we leveraged an independently generated longitudinal multiomic cohort from a previous study that collected integrated mass cytometry and aptamer-based proteomic data during pregnancy [19]. Gestational age at sample collection was aligned between the two studies by binning into matching time intervals, restricting the analysis to T2 and T3 timepoints. When multiple samples from the same patient fell within a single time interval, the earliest sample was retained. Only patients with samples at both T2 and T3 were included, yielding a test set of 6 sPTB samples (N=3) and 8 term samples (N=4). The analysis was restricted to overlapping features, i.e., the same immune cell frequency features and proteins measured by both the training and test cohort platforms (matched by UniProt identifiers) (Supplementary Table 5,6).

To account for batch effects between cohorts, features in the SciTranslMed data set were standardized (z-scored) within the cohort rather than applying the training cohort's preprocessing parameters. This approach removes cohort-level location and scale effects while preserving within-cohort relative differences, enabling evaluation of whether the learned model weights maintain discriminative ability in an independent data set. For the frequency and proteomic submodels, model scores were computed as the linear combination of standardized feature values weighted by the corresponding Lasso coefficients from the training study. Submodel scores were then standardized and combined using the stacked generalization weights to produce a final ensemble score. Model discrimination in the cohort was evaluated using the area under the ROC curve (AUROC) with 95% confidence intervals, and group differences were assessed with the Wilcoxon rank-sum test.

Reviewer #3:

General comment from Reviewer 3 about key results: In this study the authors evaluate the immunological landscape in the important problem of spontaneous preterm birth. They describe features and cell responses using an impressive multi-omics approach. Some of the strengths of this work is the use of mixed models, the longitudinal matched data, and the specifications of a consistent preterm birth cohort (all spontaneous, no iatrogenic cases).

Authors' response:
We appreciate the reviewer's positive acknowledgement of our study design and data generation.

Reviewer 3's comment #1 regarding study population: Please specify how iatrogenic cases were excluded. Lines 110-112 suggest that both medically indicated iatrogenic PTB and risk factors (e.g. cervical insufficiencies) guided the inclusion/exclusion criteria. However, line 593 states exclusion as whoever experienced iatrogenic PTB. I find the sentence on results sections can be misleading.

Authors' response:
We apologize for the confusion regarding these two statements. Due to the frequent monitoring of the PRINCE participants, we were able to stratify all PTB cases (N=40) into medically indicated, iatrogenic PTB cases (N=16), and cases with spontaneous onset of PTB (N=24). Examples resulting in iatrogenic PTB include worsening maternal health due to severe preeclampsia, fetal distress or growth restriction, vaginal bleeding, or confirmed vaginal or ascending uterine infections. Notably, relevant and significant risk factors for PTB, such as twins, chronic maternal infections, or severe maternal disease, were exclusion criteria in the PRINCE study design and thus did not affect the selection of cases in the present study. However, we did not generally exclude cases with potential risk factors for PTB from our analysis, as these are multifaceted (obesity, lower income, high stress levels, pregnancy spacing) or routinely not screened for (genetic factors).

In order to improve clarity, we have revised the results section as follows:

Cases with clinical conditions associated with iatrogenic PTB due to e.g., worsening maternal health due to severe preeclampsia, vaginal bleeding, or confirmed vaginal or ascending uterine infections were excluded. Notably, relevant and significant risk factors for PTB, such as twins, chronic maternal infections, or severe maternal disease, were exclusion criteria in the PRINCE study design and thus redundant for sample selection in the present study.

Additionally, we have expanded the explanation in the M&M section as follows:

In total, N=40 pregnancies with PTB were identified among the N=761 participants of the PRINCE study (PTB rate: 5.5%). Women who experienced iatrogenic PTB (e.g., induced delivery before gestation week 37 due to preeclampsia, fetal distress or growth restriction, vaginal bleeding, or uterine infections were excluded from the present analyses (N=16), resulting in a cohort of 24 women with spontaneous PTB (sPTB). Notably, relevant and significant risk factors for PTB, such as twins, chronic maternal infections, or severe maternal disease, were exclusion criteria in the PRINCE study design. It is well known that risk factors for PTB can be multifaceted and include, e.g., obesity, lower income, high stress levels, and short pregnancy spacing. We controlled for these risks by a clear set of matching criteria when selecting the reference cases from the group of women with term pregnancies.

Reviewer 3's comment #2 regarding study population: If possible, authors should provide details of cohort description of validation cohorts (line 836), particularly if samples were collected from similar risk population, and if spontaneous/iatrogenic cases were included.

Authors' response:
We have now included additional information on the validation cohort characteristics, as detailed in the response to Reviewer 1, Comment #6 and added the following to the manuscript in methods and results:

Results:
In brief, the validation cohorts differ significantly in geographical range (six sites in Asia, Africa, and North America) and race (Asian, African, African American) from the test cohort, which is Middle European and Caucasian. These discrepancies might explain the lower model performance in these data sets. Despite these differences, β–NGF, IL-20Ra, and CD70 are predictive across all data sets, suggesting generalizability across demographic domains, which will require additional validation in future studies.

Methods:
Jama cohort [78]
The plasma samples in the Jama study were derived from five biorepository-supported cohorts in Matlab, Bangladesh; Lusaka, Zambia; Sylhet, Bangladesh; Karachi, Pakistan; and Pemba, Tanzania. A total of 81 pregnant women were included, of which 39 had PTBs (48.1%) and 42 had term pregnancies (51.9%). Medically-indicated preterm deliveries were excluded. The mean (SD) maternal age was 24.8 (5.3) years. The median GA at sampling was 13.6 weeks.

SciAdvances cohort [14]
Plasma samples in the SciAdv study were derived from five biorepository-supported cohorts in Matlab, Bangladesh; Lusaka, Zambia; Sylhet, Bangladesh; Karachi, Pakistan; and Pemba, Tanzania. This cohort was entirely separate from the Jama cohort [78]. 113 pregnant women with a PTB (48.9%) and 118 with a term delivery (51.1%) were included, of which N=224 (97.8%) occurred spontaneously. Median (IQR) maternal age was 25 (22-29). Samples were collected before gestational week 20.

Dream cohort [20]
Samples in the Dream cohort were derived from the Perinatology Research Branch, Wayne State University, and the Detroit Medical Center in the US. A bulk transcriptomic data set of N=65 controls and N=34 sPTB samples was available with 2 – 5 samples per participant. Mean maternal age (IQR) was 25 (21-28), 23.5 (21-27), and African American race 93.8%, 88.2%, respectively. Blood samples were collected at the time of prenatal visits, scheduled at four-week intervals from the first or early second trimester until delivery, during the following gestational-age intervals: 8-16 weeks, 16-24 weeks, 24-28 weeks, 28-32 weeks, 32-37 weeks, and > 37 weeks.

In the same study, a total of n=210 plasma samples (2 samples per participant) were available from N=39 controls, N=62 sPTB, and N=4 PPROM cases. Mean maternal age (IQR) was 25 (22-27), 24 (21-28), and 24 (21.8-26.2), and African American race 87.2%, 95.2%, and 75%, respectively. Blood samples were collected at the time of prenatal visits, scheduled at 17-22, and at 27-33 weeks gestation.

SciTranslMed cohort [19]
Samples in the STM study were collected from N=63 pregnancies with spontaneous onset of labor at term (N=58) or preterm (N=5) at a single North-American site. Median (IQR) maternal age in the training cohort (N=53) was 33 (30, 35) and White, East Asian, African American race 35.9%, 30.2%, 0%, respectively. Blood samples were collected in the last 100 days before delivery; time to labor at sampling was −36 (−71, −15).

Reviewer 3's comment #3 regarding data interpretation: I appreciate the effort detecting low-density granulocytes as this is such a small proportion of cells, and is impressive to detected features associated with this specific population. However, the link to NLR ratio (line 305) should be carefully interpreted, as NLR hospital data works with whole blood, which would

include in its majority normal-density granulocytes, while here is a small proportion from the PBMC fraction.

Authors' response:
We agree with the reviewer that we should be careful when comparing our measurements of low-density granulocytes to those of the entire population of granulocytes measured in clinical settings. We have revised our statement accordingly:

While low-density granulocytes within the PBMC fraction do not necessarily linearly reflect the proportion of total granulocytes, this finding is consistent with prior studies suggesting PTB is associated with a high neutrophil-to-lymphocyte ratio as determined using whole blood in clinical settings.

Of note, our new validation data further support our finding of increased granulocyte frequencies as a feature of sPTB. In both Dream (single-omic validation, Supplementary Fig. 9c) and SciTranslMed (multivariable model validation, Supplementary Fig. 4e-g, Supplementary Table 5), whole blood granulocyte frequencies were increased in sPTB compared to sTB.

Reviewer #4:

General comment from Reviewer 4 about key results: The goal of this paper was to characterize immune response in maternal blood preceding sPTB, for a better understanding of the pathophysiology and development of predictive biomarkers. This study is extremely well designed and uses rigorous statistical assessments including validation of their predictive test in a holdout data set. The authors extract significant biological meaning from their multi-omics data by applying sophisticated network approaches to understand underlying biology of PTB. They also identify maternal immune signals that predict PTB weeks/months before delivery, which is a very important contribution to this field. Their study design leverages the existing PRINCE cohort, which had collected longitudinal data throughout pregnancy, which addresses ongoing challenges in this research field. The manuscript is well written and does a great job contextualizing their work within the larger body of maternal-fetal immune signaling in pregnancy. I offer some suggestions to strengthen this paper further.

Authors' response:
We thank the Reviewer 4 for acknowledging the important contribution of our work to the field.

Reviewer 4's comment #1 regarding data analysis and interpretation: The authors highlight their own work and important literature surrounding the role of stress in the etiology of sPTB in the introduction (Lines 86-90), and have very robust measures of maternal stress in their population (Methods, lines 624-635). In the results, they identify a relationship between maternal stress in the third trimester and sPTB (Line 121). They this potential mechanism at a molecular level in immune cell sub-populations, as they investigate an adrenergic response to a catecholaminergic agonist, which was very innovative. However, I had a hard time following why the stress measures were not further integrated into the multi-omics assessment. It would be interesting to see if these stress markers were correlated with immune and protein analytes in T3 that were predictive of sPTB, or perhaps even statistically mediated these relationships. However, this would add additional analyses to an already highly complex manuscript. The authors could consider dropping the EPDS measures altogether to tighten the focus.

Authors' response:

We appreciate Reviewer 4's interest in this aspect of our study. During manuscript preparation, we extensively discussed whether to incorporate the psychometric data (EPDS score) into the multi-omics analysis. Ultimately, we chose not to include these survey scores or additional clinical and psychosocial information as a separate layer in the multi-omics model, as this would have increased the manuscript's complexity. We thank the reviewer for the suggestion to omit the EPDS and PSS measures to keep the narrative more focused, and we have removed these measures in the revised version.

Reviewer 4's comment #2 regarding study population and confounding factors: The authors consider potential confounders including maternal age, bmi, gravidity/parity, history of PTB, and fetal sex. Did the authors have data on the rates of maternal alcohol use or smoking? These could represent additional important confounders since these are also risk factors for PTB and are associated with molecular changes during pregnancy.

Authors' response:
We appreciate the reviewer's comment, which highlights the ongoing importance of smoking and alcohol use during pregnancy. However, both factors were designated as exclusion criteria in the PRINCE study. We did, however, inquire about alcohol intake and smoking habits during the initial visit using standardized questionnaires. This revealed that 10.4% of participants reported smoking until pregnancy was confirmed, after which they stopped once aware of the pregnancy. The questionnaires also asked about exposure to passive smoking, though the numbers were small. Additionally, none of the women reported alcohol consumption after

confirming their pregnancy. Therefore, we did not include smoking and alcohol as confounding variables. To address this issue, we have amended the methods section as follows:

Smoking and alcohol use, risk factors for PTB, were also designated as exclusion criteria in the PRINCE study. Still, we inquired about alcohol intake and smoking habits during the first study visit. In total, 10.4% of all participants (N=741) reported smoking during the early weeks of pregnancy with similar numbers between women with subsequent term or preterm birth, but they quit once pregnancy was confirmed. None of the study participants reported alcohol consumption after pregnancy confirmation. Therefore, we did not include smoking and alcohol as confounding variables.

Reviewer 4's comment #3 regarding reported PTB rate: The authors highlight in the discussion that this study is limited to a single site in western-Europe with a homogenous population. Can the authors also comment on the rate of PTB (5.5%) and especially spontaneous PTB (24/761= 3.1%), as these numbers are much lower than other populations?

Authors' response:
The overall PTB rate in Hamburg, Germany, is 5.9%, which is lower than the national rate in Germany (8.3%, European Perinatal Health Report, 2015–2019) and the rates across the European Union (7.2%, European Perinatal Health Report, 2015–2019) and the US (10.38%). These low numbers in Germany can be explained by the high-quality perinatal care, with universal health insurance, standardized prenatal care, and early ultrasound screening, as well as screening for infections and gestational diabetes during pregnancy. Germany's second-largest city, Hamburg, is one of Germany's wealthiest cities, its population has a higher than average income, good access to education, and strong social support systems. This high socioeconomic status is associated with lower smoking rates during pregnancy, better nutrition, earlier prenatal care, and lower rates of untreated infection, which serves as an explanation of the low overall PTB rate. These aspects are now included in the revised manuscript as follows:

The PTB rate of 5.5% in our study is lower than the national averages in Germany (8.3%), the European Union (7.2%), and the US (10.4%) [61,62]. This lower rate may be attributed to high-quality, standardized perinatal care and universal health insurance, as well as a high socioeconomic status, specifically in Hamburg. This socioeconomic advantage is linked to better nutrition, higher physical activity, earlier prenatal visits, reduced smoking during pregnancy, and fewer untreated infections, likely contributing to the low PTB rate observed in the PRINCE study.

Reviewer 4's comment #4 regarding methodology: Line 178-179- I initially interpreted this line that the authors were looking at co-expression of proteins that were statistically different between preterm and term groups, instead of looking at the differences in correlation coefficients between the groups. This is explained more clearly in the methods. Perhaps a slight rewording here could highlight this strength?

Authors' response:
We appreciate the reviewer's attention to this detail to improve clarity. We have reworded the sentence as follows:

Correlation coefficients significantly different between term and preterm pregnancies (FDR<0.05) were highlighted (Fig. 2b).

Reviewer 4's comment #5 regarding data presentation: The authors could consider reporting the overall accuracy (AUC) of their test for predicting PTB in the holdout data set in the abstract of the manuscript, as this is a main finding.

Authors' response:

We have added this main finding to the abstract and reworded the first sentences accordingly.

Dysregulated maternal immune adaptation during pregnancy is crucial in the development of spontaneous preterm birth (sPTB). Yet, predictive models using maternal immune markers, especially before clinical symptoms appear, are still limited. In a nested case-control study within a low-risk, population-based pregnancy cohort, we decoded a distinct abnormal immune response pattern in mothers' blood that occurs weeks to months before sPTB and can distinguish cases from controls (AUROC: 0.7).

Reviewer 4's comment #6 regarding data presentation: Can the authors clarify what the X axis is in Fig. 1 B? Is each dot representing a participant?

Authors' response:
We thank Reviewer 4 for highlighting this. While each row represents the gestational age at sampling (filled circles) or delivery (open circles), each column of the graph represents a participant, with term cases in blue and sPTB in yellow. We have added the x-axis label "Study Participants" to the graph in Fig. 1b, as shown in the following revised main figure 1b:

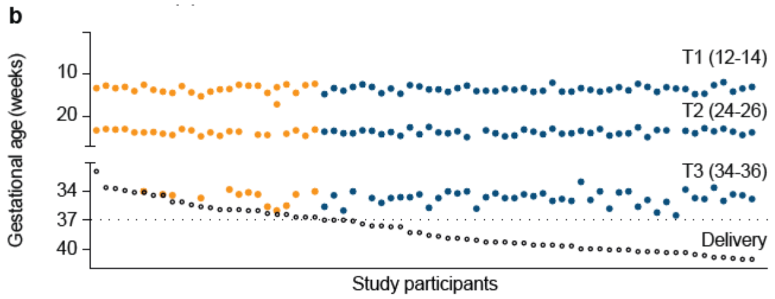

Point-by-point reply to reviewers' comments on manuscript NCOMMS-25-78183A

Reviewer #1: General remarks to the Author

I appreciate the author's detailed response and revisions to the comments. No further comments.

Reviewer #3: General remarks to the Author

Dear Editor and Authors,
Thanks for the opportunity to review the updated manuscript.
Authors have incorporated all feedback, in particular the addition of information about the validation cohorts, strengthening the comparisons findings. This reviewer has no further concerns with this manuscript.

Reviewer #4: General remarks to the Author

The authors revisions to the manuscript have addressed my concerns and have strengthened the manuscript. The addition of supplemental Figure 4 highlights the authors efforts to prevent data leakage and better explain the study design.

Authors' response to Reviewers 1, 3, and 4

We thank reviewers #1, 3, and 4 for their positive final evaluations. We have edited the manuscript in response to the comments remaining from Reviewer #2:

Reviewer #2: General remarks to the Author

The authors have made substantial efforts to address the concerns raised in the previous review, including improved methodological transparency and additional analyses. The longitudinal design and depth of multi-omics profiling remain important strengths of the study.

However, key concerns remain insufficiently resolved. In particular, the limited sample size, lack of robust independent validation, and continued positioning of the work as a predictive modeling study still constrain the strength of the conclusions.

Specific points raised by Reviewer 2

Reviewer 2's point # 1: Validation remains insufficient
The external validation relies on a very small cohort (N=3 sPTB cases), which precludes a meaningful assessment of predictive performance. While consistency in effect direction is noted, this does not constitute robust validation, and the continued presentation as "successful validation" is not supported. The authors should reframe this as preliminary evidence and avoid overinterpretation of performance metrics.

Authors' response
We have reframed the description of the validation efforts. To avoid overinterpretation of performance metrics, we now state that, while consistency in effect direction is evident, these analyses do not constitute robust validation.

We have updated the validation paragraph accordingly:

"*Finally, we* ~~successfully validated~~ *evaluated the performance of this model in an external, publicly available data set of whole blood samples serially collected from sPTB and TB pregnancies* [19]*, reaching an AUC of 0.92 in the integrated model (Supplementary Fig. 4e-g, Supplementary Table 5,6). While the results require additional validation in a larger patient cohort, the magnitude and consistency in effect direction observed in the model scores on the test set provide supportive evidence of the* ~~robustness and~~ *external validity of our multi-omic model.*" (lines 218-223)

Reviewer 2's point # 2: Study positioning

The manuscript continues to emphasize predictive performance despite limited cohort size and lack of validation. The study would be more appropriately framed as an exploratory longitudinal multi-omics analysis rather than a predictive biomarker model.

Authors' response

We have largely removed the framing of the study as predictive and replaced it with the aim to explore the immunopathology preceding preterm birth. The remainder is the description of the overall need for predictive markers, for which this study can provide a foundation.

Along this line, we have changed the manuscript title to "*Immunological Maladaptation Preceding Spontaneous Preterm Birth in Human Pregnancies*".

We have also re-written the abstract accordingly, it now reads as follows:

"The majority of spontaneous preterm births (sPTB) occurs without identifiable clinical indications or apparent risk factors. A dysregulated maternal immune adaptation at delivery has been associated with sPTB. Yet, a precise understanding of maternal immune dynamics preceding sPTB remains lacking. Here we show, in a nested case-control study within a low-risk, population-based pregnancy cohort, that an abnormal immune adaptation in mothers' blood precedes sPTB by weeks to months, and discriminates sPTB cases from full-term controls (AUROC: 0.7). Prominent immunoproteome features include heightened sensitivity to adrenergic signals within myeloid and T cell populations in early pregnancy, followed by increased production of pro-inflammatory cytokines in the third trimester. Transcriptome analysis of single-cell $CD4^+$ T cells reveals a Th17-skewed, neuroactive-protein responsive phenotype. Our study provides a multi-omics resource and a conceptual framework for early identification of individuals at increased risk for sPTB with broad translational implications for advancing targeted preventive measures."

We further introduce the study objective and long-term goal in the last paragraph of the introduction as:

"The main study objective is to model alterations of maternal immune adaptation to pregnancy across all trimesters before the onset of sPTB. Our study also offers a comprehensive multi-omics resource for use in frameworks designed to understand the dynamics of immune responses during pregnancy." (lines 114-118)

The section describing the classification/prediction model has the following title:

"*Integrative modeling reveals gestational timepoint-dependent immunopathobiology preceding sPTB.*" (lines 151-152)

Finally, we revised the last sentence of the discussion to re-emphasize the exploratory generation of a data set that aims at being a source for hypothesis generation and future study design. It now reads as follows:

"*In conclusion, our investigation elucidates several pathways associated with sPTB, offering a valuable resource for formulating hypotheses and informing future mechanistic studies. Our research underscores the significance of generating comprehensive phenotypic and functional cell atlases over a defined temporal framework to enhance our understanding of sPTB pathobiology.*" (lines 555-562)

Reviewer 2's point #3: Sample size and power
The post-hoc power analysis demonstrates detectability above chance but does not address model stability, overfitting, or reproducibility. These limitations should be more explicitly acknowledged.

Authors' response
We acknowledge that, while testing model stability, overfitting, and reproducibility are integral to the modeling approach itself, these considerations were not taken into account in the context of power analyses and to determine required patient numbers, due to the lack of prior data.

To explicitly acknowledge these limitations, we have amended the Discussion as follows:

"*Although our cohort is relatively small, as is common for discovery-stage modelling studies, we performed post-hoc power analyses to assess whether the study had sufficient sensitivity to detect discriminatory performance above chance (AUC = 0.5). These analyses indicated that the observed AUCs were detectable under the sample-size of our study. However, large international, multi-center studies with sizable, well-characterized patient groups are required to establish model stability, reduce overfitting, and establish the reproducibility and reliability of the identified signals.*" (lines 502-508)

Reviewer 2's point #4: Transcriptomic subcohort
The clarification of the paired design is helpful; however, given the small sample size (10 vs. 10), conclusions should remain at the pathway level and be interpreted as hypothesis-generating.

Authors' response
We have ensured that any language implying a definite association between transcriptomic profiles and preterm birth is avoided. We now explicitly state that the transcriptome analyses are hypothesis-generating. This section of the manuscript begins and ends, respectively, as follows:

"*This analysis was designed as an exploratory investigation to identify candidate biological pathways, not as a definitive gene-level discovery effort.*" (lines 332-334)
"*Overall, we report pathway-level associations of second-trimester single-T cell transcriptomic profiles with sPTB, providing a foundation for hypothesis generation to test causal links in future studies.*" (lines 418-420)

Minor points raised by Reviewer 2

The revisions improve clarity and methodological transparency. Minor wording adjustments could further reduce overstatement (e.g., replacing “validated” with “evaluated”).

Authors’ response

We have carefully combed the entire manuscript for overstatements. We have replaced “validated” with “evaluated”, “demonstrating” with “suggesting”, “might” with “may”, etc.

Examples include:

“*Finally, we ~~successfully validated~~ evaluated the performance of this model in an external, publicly available data set of whole blood samples serially collected from sPTB and TB pregnancies* [19], …” (lines 218-219)

“*Collectively, along with the enrichment of βNGF, orexin, and other signaling cascades related to neuroactive proteins (Fig. 4b), these findings ~~underscore~~ suggest the presence of a dysregulated peripheral neuroimmune synapse in the immunopathobiology of sPTB.*” (lines 415-418)

“*Increased receptor expression ~~might~~ may underlie the increased adrenergic responsiveness observed on protein level.*” (lines 406-407)
